# Saving millions of lives but some resources squandered: emerging lessons from health research system pandemic achievements and challenges

**DOI:** 10.1186/s12961-022-00883-6

**Published:** 2022-09-10

**Authors:** Stephen R. Hanney, Sharon E. Straus, Bev J. Holmes

**Affiliations:** 1 Health Economics Research Group, Department of Health Sciences, Brunel University London, London, United Kingdom; 2 St Michael’s Hospital, Unity Health Toronto, Toronto, ON Canada; 3 Michael Smith Health Research BC, Vancouver, BC Canada

**Keywords:** COVID-19, Equity, Health research systems, Knowledge production, Knowledge utilization, Pandemic, Priority-setting, Research capacity, Research waste, Vaccines

## Abstract

During the SARS-CoV-2 pandemic, astonishingly rapid research averted millions of deaths worldwide through new vaccines and repurposed and new drugs. Evidence use informed life-saving national policies including non-pharmaceutical interventions. Simultaneously, there was unprecedented waste, with many underpowered trials on the same drugs. We identified lessons from COVID-19 research responses by applying WHO’s framework for research systems. It has four functions—governance, securing finance, capacity-building, and production and use of research—and nine components. Two linked questions focused the analysis. First, to what extent have achievements in knowledge production and evidence use built on existing structures and capacity in national health research systems? Second, did the features of such systems mitigate waste? We collated evidence on seven countries, Australia, Brazil, Canada, Germany, New Zealand, the United Kingdom and the United States, to identify examples of achievements and challenges.

We used the data to develop lessons for each framework component. Research coordination, prioritization and expedited ethics approval contributed to rapid identification of new therapies, including dexamethasone in the United Kingdom and Brazil. Accelerated vaccines depended on extensive funding, especially through the Operation Warp Speed initiative in the United States, and new platforms created through long-term biomedical research capacity in the United Kingdom and, for messenger ribonucleic acid (mRNA) vaccines, in Canada, Germany and the United States. Research capacity embedded in the United Kingdom’s healthcare system resulted in trial acceleration and waste avoidance. Faster publication of research saved lives, but raised challenges. Public/private collaborations made major contributions to vastly accelerating new products, available worldwide, though unequally. Effective developments of living (i.e. regularly updated) reviews and guidelines, especially in Australia and Canada, extended existing expertise in meeting users’ needs. Despite complexities, effective national policy responses (less evident in Brazil, the United Kingdom and the United States) also saved lives by drawing on health research system features, including collaboration among politicians, civil servants and researchers; good communications; and willingness to use evidence. Comprehensive health research strategies contributed to success in research production in the United Kingdom and in evidence use by political leadership in New Zealand. In addition to waste, challenges included equity issues, public involvement and non-COVID research. We developed recommendations, but advocate studies of further countries.

**Supplementary Information:**

The online version contains supplementary material available at 10.1186/s12961-022-00883-6.

## Background

The COVID-19 pandemic yielded astonishing and often rapid [1] research achievements that helped avert the deaths of millions of people worldwide [2–8]. The Randomised Evaluation of COVID-19 Therapy (RECOVERY) trial was set up in 9 days in the United Kingdom (UK), and 3 months later it identified dexamethasone as the first treatment proven to reduce mortality in critically ill patients, enabling its immediate implementation globally [1–4, 9, 10].

Years of research on messenger ribonucleic acid (mRNA) in the United States (US) and Germany, plus other vital research including at the University of British Columbia (UBC), Canada, provided a new platform on which vaccines were developed in record time [11–17]. Similarly, years of research at the Jenner Institute, Oxford University, UK, provided a different new platform on which the Oxford/AstraZeneca vaccine was developed equally rapidly [18, 19]. Animal trials of the Oxford vaccine were conducted in long-established publicly funded facilities in Australia and the United States [17]. Trials in Brazil made key contributions to, first, the Oxford vaccine [18–20], and second, the pool of evidence used in both the United Kingdom-led living (i.e. regularly updated) systematic review confirming that corticosteroids were effective against COVID-19, and the Canadian-led WHO living guideline [21–23]. These examples of knowledge production and product development were almost immediately available beyond the country conducting the research and development (R&D), but for a variety of reasons, access to them varied greatly.

Facilitating evidence use for improving practice and policy, especially within countries, also evolved quickly during the pandemic, including through public health legislation. In March 2020, a team of researchers and clinical stakeholders in Australia collaborated to produce national, living evidence-based guidelines for COVID-19—they were updated weekly and widely used [24–26]. New South Wales (NSW) Health, the public healthcare system in the most populous Australian state, funded a programme of rapidly co-produced research that informed the state’s COVID-19 policies [27]. In New Zealand, the political leaders worked well with both the science community, including in co-producing local evidence, and science communication experts [28–30], so that the country was able to take full advantage of its relatively isolated location and ability to close its borders, and consequently benefited by having a COVID-19 death rate much lower than most other countries. New Zealand became one of the few global exceptions by having a negative excess deaths figure, that is, fewer deaths than the usual number in a pre-pandemic year [31, 32], and a lower level of years of life lost than expected [33].

Alongside the rapid achievements, research resources appear to have been wasted on an unprecedented scale [34]. Especially in the early months of the pandemic, many primary studies were too small or poorly designed to produce useful findings, many studies were not completed, and there was duplication with multiple primary studies, systematic reviews and guidelines on the same topic conducted in the same context [1, 34–42]. From an assessment of worldwide registered COVID-19 trials, the US Food and Drug Administration (FDA) concluded, “the vast majority of trials of therapeutics for COVID-19 are not designed to yield actionable information” [35]. A further source of waste occurred in countries such as Brazil and the United States, in particular, where despite their scientific achievements, scientific evidence has sometimes been spurned by policy-makers in favour of populist rhetoric that has impaired the countries’ ability to manage the crisis [43]. Even when evidence has been welcomed by policy-makers, sometimes the complexities of equitable and effective decision-making have resulted in frustration and delays.

In the context of such achievements and challenges, questions arise about the role of a nation’s health research system (HRS) [44]. Such a system is defined by WHO as “the people, institutions, and activities whose primary purpose in relation to research is to generate high-quality knowledge that can be used to promote, restore, and/or maintain the health status of populations; it should include the mechanisms adopted to encourage the utilization of research” [45]. In launching a collection of papers in *Health Research Policy and Systems* on the response of HRSs to the pandemic, Yazdizadeh et al. (2020) argued it was important to learn lessons from the achievements and challenges [44]. Discussion of how a nation’s HRS has responded to the pandemic is best informed by a formal systems approach [44], and when identifying lessons and recommendations it is useful to draw on the framework for HRSs developed by WHO [45]. This framework identified four main functions and nine components of HRSs. These are presented below, although to assist our analysis we have swapped the order of the final two components:

1. *Governance/stewardship* [components: (1) vision [now coordination]; (2) priority-setting and coordinating adherence to them; (3) ethics; (4) monitoring and evaluation]
2. *Financing* [(5) securing and allocating accountably]
3. *Capacity-building* [(6) human and physical capacity to conduct, absorb and utilize health research]
4. *Producing and using research* [(7) produce outputs; (8) promote use of research to develop new tools—drugs, vaccines, devices, etc., to improve health; (9) translate and communicate research to inform health policies, strategies, practices, public opinion]

Transposing the final two components helped facilitate the construction of two questions—the first with two parts—to guide our analysis. Addressing the two questions helped focus the lessons by encouraging analysis of how each component of an HRS, and an overall strategy, might have contributed to the saving of lives and the avoidance of waste. The questions are as follows:

- A(i) How far have the structures and capacities of the HRS contributed to generating life-saving knowledge available globally, and its translation into products?
- A(ii) How far have the structures and capacities of the HRS contributed to the utilization of evidence to inform the development of healthcare practice and policies to save lives, including non-pharmaceutical interventions (NPIs) primarily within the relevant jurisdiction?
- B How far have the structures of the national, or subnational, HRS prevented, or reduced, the squandering of research resources?

Question A(i) relating to the production of life-saving knowledge and its translation into products is not straightforward, and in practice there were also limitations on the global access to the products such as vaccines. However, addressing question A(ii) is even more challenging. It involves examining, for each jurisdiction considered, to what extent the HRS contributed to the use of locally and/or globally produced relevant evidence to inform healthcare practice and policies, especially NPIs, that reduced the death rate. Different factors could come into play for policies than for practice, and the death rates from COVID-19 varied greatly between different jurisdictions. In terms of total numbers of recorded COVID-19 deaths reported by each nation, as of 31 December 2021 the United States was highest with 846,905 and Brazil second highest with 619,109, while New Zealand had just 51 [46]. Even allowing for New Zealand’s low population of just 5 million, this still represented a much lower death rate than most other countries. But while New Zealand’s relatively isolated location and ability to close its borders were also noted earlier, during a global pandemic it still required appropriate policies to be adopted to take advantage of such opportunities.

Analysis of A(ii) will, therefore, involve drawing on several streams of pre-pandemic thinking. First, this includes a recognition that consideration of the political and situational contexts around the extent of evidence use is critical and that evidence is only one input into policy-making [47, 48]. A second strand of thinking involves an understanding of the ways in which HRSs can facilitate evidence use through approaches such as promoting a culture of evidence use and encouraging collaboration between researchers and research users [49]. There is a need, therefore, in considering question A(ii) to further explore the generation and linking of evidence in public health emergencies [50–53], including the political and social aspects, and how these aspects might relate to HRSs more generally.

During the pandemic, irrespective of the success of their research, health researchers globally have responded vigorously, with Turner and El-Jardali noting, “It turns out that, when the going gets tough, researchers get productive, collaborative and impact focused” [54]. Kim and Chou identified contributions to 2020 COVID-19 publications from authors from 158 countries across the income spectrum [55]. Nevertheless, there was considerable disparity between nations in the extent of both their production of research/development of products, and their use of evidence in policy-making. Furthermore, there were concerns in many nations about the disparity of the pandemic’s impact on populations within the country, especially on the health of diverse ethnic minority populations, and whether there was sufficient research capacity to analyse such issues adequately [56]. Therefore, to facilitate progress in addressing our two questions in the face of such an enormous potential agenda, we chose to focus on seven countries where the HRS made differing contributions, and from which we hoped to draw lessons. But we also encourage a robust analysis of HRSs in other contexts and expect our approach and some of our lessons could inform such studies.

### Selecting seven systems

This opinion paper attempts to identify some major emerging lessons from a focused analysis of key articles and commentaries related to one or more of seven countries: Australia, Brazil, Canada, Germany, New Zealand, United Kingdom and United States. These were selected because of major, but diverse, contributions they have made to the research response to the pandemic—they are similar in key ways, but differ markedly in others. All seven are democracies with an established focus on producing health research and have reasonably accessible information about how their respective HRSs contributed to the science. With the exception of the New Zealand system, which is inevitably small given the country’s population, all our selected countries were in the top 12 in terms of number of 2020 COVID publications (the other six in the top 12 were, in descending order, China, Italy, India, Spain, France and Iran) [55]. The seven selected countries also all featured in the top 20% of the 195 countries ranked on the 2019 Global Health Security (GHS) Index, which assessed factors such as a nation’s capability to address infectious disease outbreaks [57, 58].

While Brazil was the only one of the seven countries classified as a middle- rather than high-income country, over the years it had built up a strong HRS seen as being “in line with the constitutional right of universal health access and universal health coverage” [20]. Therefore, when identifying lessons from the COVID-19 response of the seven HRSs, one issue will be the extent to which Brazil might have lessons for other middle-income countries in terms of what could be achieved.

The seven countries differ in the nature of their government structures: five are federal states, New Zealand has a unitary government, and the United Kingdom has a more complicated pattern in which England has about 85% of the population, and the UK Government makes decisions for England that in the other three nations (Scotland, Wales and Northern Ireland) are made by their own administrations. There are associated differences amongst our seven countries on how healthcare is funded (e.g. at the national level) and delivered (e.g. at the provincial/state level) and, similarly, how research is funded.

In most federal countries the major health or medical research council operates at a national level (e.g. in Australia, the National Health and Medical Research Council [NHMRC]; in Canada, the Canadian Institutes of Health Research [CIHR]; and in the United States, the National Institutes of Health [NIH]), with varying governance arrangements. Even though in federal countries most healthcare is usually not delivered at a national level, some national health departments still have major research funding programmes; for example, the US Department of Health and Human Services is the sponsoring department not only for the NIH, but also for the Biomedical Advanced Research and Development Authority (BARDA) [59]. In federal countries, healthcare is often delivered at a subnational level, and there are usually some research funding organizations at that level. There is not space in this paper to give examples from every subnational organization in the seven countries, but some examples will be given to illustrate key points.

The exact nature of the concern about the impact of COVID-19 on minority communities also varied across our selected countries. For example, in the five countries outside Europe there were particular fears that health inequities already suffered by Indigenous populations might be exacerbated during the pandemic, and result in higher death rates.

Finally, the seven countries are also in very different positions on the spectrum of level of success in controlling the pandemic. As noted, New Zealand had very low death figures, the United States and Brazil the highest recorded anywhere, and our other four countries were spaced out between them across the spectrum of success in averting deaths from COVID-19.

A complication is that countries vary in the way they record and publish the number of COVID-19-related deaths. Therefore, Table 1 presents both the COVID-19 deaths per million population using figures stated by each of our seven countries [46], and, in the first column, figures per million population in the respective countries for excess deaths as independently estimated by experts from *The Economist* (i.e. “the number of deaths from all causes during a crisis above and beyond what we would have expected to see under ‘normal’ conditions”) [31]. This more independent analysis shows that at the global level, the United States and Brazil, while having high figures, and higher than self-reported COVID-19 deaths, were far from the worst. Russia, Mexico, Turkey, India and many other countries in Eastern Europe and Latin America had higher numbers for excess deaths per million population than even the United States and Brazil. The independent analysis also confirms that countries such as New Zealand had fewer deaths than expected in a normal year. While some individual countries also publish figures for excess deaths which do not always quite match the ones presented on Table 1, and there are continuing disputes about the most rigorous way to assess excess deaths [60], the figures presented on the Table are produced using a standard cross-country methodology. The table also includes the percentage of the total population who had been fully vaccinated (i.e. had the required number of a two-dose, or single-dose, vaccine) as at the end of 2021. Finally, Table 1 presents each nation’s ranking on the 2019 GHS Index—which is a composite index with many elements (for example, the United States, despite being ranked highest overall, was 175th out of 195 countries on the item for healthcare access [61]).

**Table 1:** Seven selected countries: excess deaths per million population as at 27/12/2021; COVID-19 deaths per million population as at 31/12/2021; % of population fully vaccinated as at 31/12/2021 and Global Health Security Index rank as at October 2019

Country	Estimated excess deaths during COVID-19 per million population	COVID-19 deaths per million population	% of total population fully vaccinated	2019 Global Health Security Index rank/195 countries
New Zealand	−500	10	75.2	35
Australia	10	86	76.2	4
Canada	680	793	77.2	5
Germany	1350	1339	70.9	14
United Kingdom	2210	2172	70.5	2
United States of America	3010	2536	63.0	1
Brazil	3150	2882	66.9	22

Sources: Giattino et al., 2022: https://ourworldindata.org/excess-mortality-covid (adapted from table showing estimated cumulative excess deaths during COVID-19 per 100,000 people using data from *The Economist* for 27/12/2022) (accessed 10/8/2022) [31, 32]; Worldometer: COVID Live—Coronavirus Statistics—Worldometer (worldometers.info) (accessed: 1/1/2022) [46]; Mathieu et al., A global database of COVID-19 vaccinations. Nat Hum Behav. 2021;5:947–953. Web update for 31/12/2022: Coronavirus (COVID-19) Vaccinations—Our World in Data (accessed 10/8/2022) [62]; Global Health Security Index (2019): https://www.ghsindex.org/wp-content/uploads/2021/11/2019-Global-Health-Security-Index.pdf (accessed 6/11/21) [57]

We recognize that not only have many other countries contributed significantly to the science, but also developments have often relied on contributions from more than one country, and therefore, in some key areas the science in our seven countries overlaps with that of the wider global scientific community. One example discussed in more detail later is WHO’s Solidarity trial in which sites in Brazil and Canada participated along with those in many other countries [63, 64]. Nevertheless, we believe focusing on our seven countries provides considerable variation across diverse issues, while also enabling us to delve into the evidence about each nation in sufficient depth to begin to understand developments in all seven.

We start the main section of the opinion paper by briefly outlining the sources on which we drew to create a series of accounts illustrating examples of key pandemic achievements and challenges for each of the seven HRSs in turn. Then we confirm the most appropriate framework to use to organize the analysis of this material. Drawing on our accounts of the national responses, we next identify a series of lessons and attempt wherever possible to link them to features of the respective HRSs. Before making recommendations, we acknowledge that the circumstances of each country will vary in ways beyond their respective HRSs, such as location, and we also recognize that many other factors have impinged on the production and, especially the utilization, of evidence during the pandemic [65]. We also note how the pandemic has emphasized that systems have porous boundaries, and the concept of an HRS could be expanded to include items like public trust, and the analysis expanded to consider the relationship with political leadership. Finally, it has been claimed that the response of different countries to the pandemic in some ways resembles a natural experiment [43]. Our analysis sheds light on the extent to which this applies to HRSs.

## Identifying lessons and recommendations from HRS achievements and challenges during the pandemic

We mainly relied on publications to gather information about how each of our seven HRSs responded to the pandemic. We identified relevant publications in three main ways for our paper, which is the first, as far as we are aware, to undertake a comparative analysis of the response of different HRSs to the pandemic in an attempt to draw lessons for HRS improvement. The paper primarily covers 2020 and 2021, giving particular emphasis to the response in 2020 when the readiness of the HRS was especially relevant. The major source was leading weekly scientific journals. Full details about the sources of publications, and each author’s relevant experiences, are presented at the start of a separate file, “Examples of responses to the pandemic in seven health research systems” (Additional file 1), which describes the response from each HRS in turn.

### Framework for analysis and table of lessons

We required a framework to structure the analysis of the evidence from Additional file 1 in order to identify a series of lessons from the COVID-19 response. Just prior to the pandemic, WHO commissioned a study in which the WHO framework for national HRSs was used to organize an evidence synthesis of approaches for strengthening HRSs [66, 67]. The WHO review was conducted amid earlier concerns about research waste [68] and provided a useful account of the pre-pandemic state of various HRSs. In particular, it identified a key step for strengthening the HRS in a country (or subnational jurisdiction) as being the development and application of a comprehensive and coherent health research strategy covering as many HRS functions and components as possible. Examples of progress at building a well-organized HRS informed by a comprehensive and coherent strategy were reported in England^1^ and New Zealand [66, 69, 70].

In England, the National Institute for Health Research (NIHR) covers all functions of the HRS, with the intention that the HRS should be embedded into the healthcare system, namely, the National Health Service (NHS) [69, 71]. Therefore, even though its strategy did not include other major funders, the NIHR has “a central role in England’s health and care research landscape” [72]. The governance of the NIHR, especially through the incorporation of stakeholder engagement and patient and public involvement (PPI) in priority-setting and other research processes, was aimed at ensuring it met the needs of the NHS and patients. There was also increasing focus on the importance of assessing research impact on policy, healthcare and the economy [66, 67, 73, 74]. Large investments over the years built NIHR research capacity and infrastructure throughout the NHS. Biomedical Research Centres aimed to boost translational research through the co-location of leading clinical academics in major healthcare centres. The Clinical Research Network (CRN) developed and coordinated capacity to conduct clinical trials throughout the health service. This capacity contributed to the production of relevant research, thus reducing waste [66, 69, 71, 73–75].

While the many other elements of the NIHR included the funding of systematic reviews to meet healthcare system needs, and increased co-production [66], many of the NIHR’s components also occur elsewhere. For example, an analysis of the NIHR suggested more attention should be given to mechanisms to meet the needs of policy-makers—an area where Canada had gone further [73]. An NIHR review found that research-active healthcare organizations were more likely to use evidence and provide improved healthcare than non-research-active ones, but the best examples were networks in countries including Germany and especially the United States [76]. However, later, the most significant evidence for this occurring across an entire health system came from the United Kingdom’s NHS/NIHR [77, 78], thus illustrating the particularly comprehensive nature of the NIHR system and strategy.

The WHO evidence synthesis noted that the New Zealand health research strategy similarly consisted of a combination of components covering many HRS functions, and included statements about the respective responsibilities of the Health Research Council (HRC) and the two relevant ministries, Health, and Business, Employment and Innovation [70]. The strategy stated as one of four priorities, “build and strengthen pathways for translating research findings into policy and practice. The Ministry of Health will lead this work”, but with support from the other two [70]. Another priority, “invest in excellent health research that addresses the health needs of all New Zealanders”, linked to a guiding principle, partnership with “Māori communities to improve Māori health and wellbeing through research”, and the strategy’s vision to support “a vibrant Māori health research sector” [70].

The analysis in the WHO evidence synthesis was almost entirely at the national level. But one exception was identification of the comprehensive and coherent health research strategy proposed for the Canadian province of British Columbia (BC) [79]. The strategy was not fully implemented, but in addition to the continuation of bodies such as the BC Centre for Disease Control, with its role of conducting and coordinating research for public health and disease control [80], various gaps and opportunities referred to in the strategy were subsequently addressed. This was achieved through developments in research ethics, clinical trials and creation of the BC SUPPORT Unit to increase patient-oriented research, as well as by the health ministry through a provincial health data platform and research and knowledge management strategy [81, 82].

Other examples of coherent health research strategies at the subnational level include the *Population Health Research Strategy 2018–2022* from NSW Health, published after the main search for the WHO evidence synthesis was conducted [83]. While not covering the full range of health research fields, it aimed to improve health outcomes through research, and included many components of WHO’s framework. It prioritized the importance of embedding consideration of the needs of Aboriginal people, including through ethics reviews of research proposals. The strategy promoted the development and maintenance of existing research capacity, including through the Prevention Research Support Program (with its research capacity-building for NSW Health staff) and the Sax Institute in Sydney, NSW, with its role in driving research use. According to the strategy, “long-term programmatic engagement between researchers and policy makers and practitioners has the greatest potential for enhancing the quality and relevance of population health research in NSW” [83]. Translation was promoted with strategies to “develop policy and practice environments that value research”, and foster “research environments that promote the use of research evidence”.

These four examples show that the various components of the WHO framework, plus consideration of the value of an overall strategy, provide a solid basis for our analysis of HRSs. The lessons and recommendations we identify are, therefore, primarily structured around the WHO framework for analysing HRSs. Additionally, pre-pandemic analysis of how an HRS might attempt to accelerate the translation of early research into new drugs and vaccines was available to draw on, including an analysis funded by the United Kingdom’s MRC [84].

In the current paper we examine how existing strengths or gaps in specific HRS functions sometimes respectively reinforced achievements by enabling researchers to mobilize more effectively, or exacerbated the challenges in responding to the pandemic. Inevitably, there are some overlaps where achievements are linked to several functions. For example, consideration of lessons about the first component of the governance function, coordination (now used to incorporate the original narrower term “vision”), inevitably overlaps with the second component, prioritization, which became more effective at a systems level if there were coordination mechanisms for encouraging adherence to the priorities.

There are also inevitably overlaps, especially in the case of the United Kingdom and New Zealand, when we collate the analysis about each individual function and consider the value of having a well-organized HRS with an overall strategy. While this might appear somewhat repetitive, it only serves to reinforce the point that in a comprehensive and coherent HRS, the various functions and their components would often be mutually supportive. Finally, we discuss the separate lesson related to how the pandemic, and the research response to it, had some negative implications for the HRS itself. The 11 lessons are outlined in Table 2, and then elaborated.

**Table 2:** Outline of lessons for HRSs from the pandemic, organized using the WHO framework for HRSs

HRS functions/components	Lessons related to each HRS component, comprehensive strategies and negative impact
*Governance*	*Governance*
1. Coordination	1. Existing or rapidly established coordination was often the key, especially for clinical research, to effective responses and reduced risk of wasted resources
2. Priority-setting	2. Effective priority-setting was important in: rapidly testing new therapies, reducing waste of resources, considering the needs of diverse communities
3. Ethical approval	3. The ability to accelerate ethics and protocol approvals and to enhance data access and sharing increased the speed and efficiency of research production
4. Evaluation	4. The substantial and immediate benefits from rapid (but expensive) research progress provide enhanced opportunities and need for impact assessment
*Financing*	*Financing*
5. Securing finance	5. Unprecedented (but uneven) funding; public, for many pandemic topics; private, for development of vaccines and therapies; collaborative, to help achieve major successes; but widespread concerns about wasted resources
*Capacity*	*Capacity*
6. Capacity-building	6. Important contributions came from: mobilization of capacity developed over years to conduct primary and secondary research, enhanced interdisciplinary cooperation and clinical research integrated in healthcare systems
*Production and use*	*Production and use of research knowledge*
7. Knowledge production	7. Accelerating research production (new vaccine platforms, mobilized capacity, adaptive platform trials) produced results—but problems for policy research; rapid publication of findings became essential but led to dangers
8. Promote use in new products	8. Translation of research into new products to reduce mortality and morbidity often occurred at unprecedented speed and often reflected unprecedented levels of both public funding and public/private collaboration tackling the crisis
9. Translate to inform policies, practice and opinion	9. The considerable divergence in the use of evidence to inform NPI policies, etc., and to promote equity in policies, partly reflected established structures and cultures; collaborative living guidelines and good communications mattered
*Comprehensive strategies for health research*	10. Pre-existing comprehensive health research strategies and vision enhanced the effectiveness of specific steps and opportunities for producing research to improve policies, practice and health, but did not ensure informed action
*Negative impacts on HRSs*	11. The pandemic damaged aspects of HRSs: reduced resources/opportunities especially for non-COVID-19, early-career, female and minority researchers; problems completing projects in lockdowns; reductions in public involvement

Source: Extensively adapted from Pang et al. (2003) [45] and Hanney et al. (2020) [66, 67]

### Lesson 1—Coordination: existing or rapidly established coordination was often the key, especially for clinical research, to effective responses and reduced risk of wasted resources

From examples across our seven countries we can identify at least three broad categories of research coordination: first, pre-pandemic coordination of attempts to enhance research preparedness for a pandemic and to develop a readiness to respond; second, attempts during the pandemic to build mechanisms for greater coordination in the research response once the need was realized; third, the mobilization during the pandemic of coordination mechanisms that already existed across the HRS in line with the existing health research strategies, such as in those three described above in the United Kingdom, New Zealand and NSW.

Most of the seven HRSs we are considering had some elements of these categories, but we shall focus on aspects of the strongest examples that are most relevant for our analysis. Pre-pandemic building of coordinated research preparedness had occurred in Australia through the 13 organizations which were members of the Australian Partnership for Preparedness Research on Infectious Disease Emergencies (APPRISE). It responded to COVID-19 by activating a preplanned research platform on 13 January 2020 [85]. Sharon Lewin, Director of the Peter Doherty Institute for Infection and Immunity, Melbourne Hospital, Victoria, and chief investigator for APPRISE, explained how work conducted since 2016 to prepare for a pandemic “enabled us to fast track our COVID-19 related research” [86]. An early study of concomitant immune responses prior to recovery conducted at the Doherty Institute attracted global attention when published in *Nature Medicine* on 16 March 2020, and the authors acknowledged the role of the platform activated by APPRISE [87].

Once the pandemic started there was a realization in many countries that greater coordination of research activities would be valuable. In Brazil, physician scientists collaborated to create the Coalition COVID-19 Brazil initiative that, it was claimed in December 2020 by Zimerman et al., “encompasses more than 70 centres around the country and has been leading 11 randomised clinical trials with more than 5000 participants. Brazil is a developing country….for which this level of coordination is unprecedented. In fact, rarely has it been achieved in developed countries” [88]. The coalition’s trials made important contributions regarding various potential therapies. These included a trial showing the effectiveness of dexamethasone [21], which was published in the *Journal of the American Medical Association* (*JAMA*), cited almost 400 times, and contributed 19% weight (the second-highest contribution after RECOVERY) to an early WHO review supporting the use of corticosteroids [22].

In April 2020, Innovation, Science and Economic Development Canada, along with the support of the Chief Science Advisor, created CanCOVID as a Canada-wide network of health, science and policy researchers to facilitate COVID-19 research collaboration [89]. Also, in relation to a coordinated approach to resources, on 23 April Prime Minister (PM) Trudeau announced a large investment in “a national medical research strategy to fight COVID-19” through various new and existing programmes covering the full range of research [90]. But the need for further coordination was identified in late 2020 as CIHR drew on some analysis from early in the pandemic [91] to state, “At this stage of the pandemic, the gap in Canada’s clinical trials coordination infrastructure has once again been noted as an area in need of improvement” [92]. While the role of some individual triallists and groups was noted, “collaboration and coordination mechanisms across these groups have not been specifically funded” [92]. CIHR, therefore, launched a call for proposals for greater coordination of research capacity and activities, possibly through a “network of networks” [92].

Within a province such as BC, the pre-pandemic steps towards greater coordination of the HRS were built on in various ways that strengthened coordination of the response to COVID-19 [93]. For example, in BC, stakeholders came together to establish the COVID-19 Clinical Research Coordination Initiative as well as a COVID-19 Strategic Research Advisory Committee to bridge the Provincial Health Officer, government decision-makers and the BC health research community [93].

In spring 2020, the German Federal Ministry of Education and Research announced the creation of the "Network University Medicine" to include all 34 university hospitals in Germany working in various combinations on COVID-19 projects. However, it was not until the beginning of October that the first main tranche of funded projects was announced. In addition to addressing a range of COVID-19 issues, one network goal was “generation of findings also for better preparation for future epidemiological events” [94].

In the United States, research leaders such as the Director of the NIH, Francis Collins, quickly sought to take action once it was realized that “much-needed coordination among important constituencies was lacking” [95]. This led to the NIH working with the private sector to create the Accelerating COVID-19 Therapeutic Interventions and Vaccines (ACTIV) public–private partnership platform launched in April 2020. Its main goals were “to establish a collaborative framework for prioritizing vaccine and therapeutic candidates, to streamline clinical trials and tap into existing clinical trial networks” [95]. Then, in turn, in May 2020 the United States Government created Operation Warp Speed (OWS), with a very large budget, to coordinate work of the Health and Human Services Department’s organizations, including the NIH, ACTIV and BARDA, and others including the Defense Advanced Research Projects Agency (DARPA) programme of the Department of Defense [59, 96, 97]. DARPA already had a Pandemic Prevention Platform that funded a coordinated programme of research, including from 2018 at AbCellera, a spin-off company from UBC in Vancouver, Canada, “to establish a robust technology platform for pandemic response capable of developing field-ready medical countermeasures within 60 days of isolation of an unknown viral pathogen” [98]. As described in more detail below, OWS had some notable successes in terms of vaccines in particular, but also in contributing to the development, testing and rapid deployment of some new therapies.

Despite the efforts above, leading researchers in various systems suggested there were problems with insufficient coordination and prioritization. Such analysis was published in leading national medical journals about Canada, online in December 2020 [99], the United States in March 2021 [100], Australia in July 2021 [101], and across Europe in December 2021 [39]. Where appropriate, all four papers will be described in more detail later, but they all also highlighted the greater progress that had been made in the United Kingdom, with its more extensive collaboration and prioritization. Similar points about the greater success of the UK system had already been made by various commentators following publication of the findings of the RECOVERY trial in mid-2020 [36, 40, 41].

The way the pre-existing research strategies in NSW, New Zealand and the United Kingdom assisted the pandemic response is highlighted in various subsections below. Therefore, in most countries, to varying degrees, there was some coordination and priority-setting beyond the level of the individual researchers, particularly for clinical research. However, the way these first two components of the governance function operated in the United Kingdom stood out in terms of providing lessons on how to achieve the comparatively successful results described in Additional file 1 and below.

### Lesson 2—Priority-setting: effective priority-setting was important in: rapidly testing new therapies, reducing waste of resources and considering the needs of diverse communities

#### Implementing priority-setting for rapid progress in the United Kingdom

Researchers in the United Kingdom had become accustomed to a system in which there was a focus on clinical research priorities relevant to the needs of the healthcare system being set by recognized stakeholders, including the researchers, because it was one of the key aspects of the NIHR in the United Kingdom [69, 71, 73]. Therefore, perhaps it was not surprising that of the various countries where emergency prioritization procedures were introduced to address the initial crisis, the United Kingdom was seen as having gone further in identifying a small number of top priorities. These were designated as Urgent Public Health priorities, and included the RECOVERY, Randomised, Embedded, Multifactorial Adaptive Platform trial for Community-Acquired Pneumonia (REMAP-CAP) and Oxford vaccine trials [1, 10, 102]. As discussed below in Lesson 7 on knowledge production, the type of trial design used—i.e. the focus on including selected adaptive platform studies among the priorities—was also seen as being very important, and proposed as a reform in other countries. In Australia, researchers leading the AustralaSian COVID-19 Trial (ASCOT) referred to recruitment challenges and called, during a pandemic, for “a small number of national platforms in Australia, similar to RECOVERY in the UK” [101].

While the concept of prioritizing research on the needs of the healthcare system was not new in the United Kingdom, the mechanisms through which it was undertaken at the start of the pandemic were very different from usual. It was undertaken by a small group of central players who made the prioritization choices between projects proposed by researchers within the system, but without the usual level of consultations with the wider practitioner, patient or research communities [10, 102]. As emphasized in the original WHO HRS framework, mechanisms for coordinating effective implementation of prioritization are also important [45]. In the United Kingdom, the pre-existing NIHR’s CRN—a network covering clinical researchers in all acute hospital trusts—was crucial for the effective focusing of resources, not only on selected topics, but also on the specific studies within the key topics that could access NIHR/CRN resources [1, 3, 10, 102–106].

The ability of the UK system to enforce the prioritization was linked to the wider governance structures of the HRS embedded in the healthcare system. This meant that the chief medical officers of the UK nations, including Chris Whitty in England who also headed the NIHR, could take decisive action. In the exceptional circumstances at the start of the pandemic response, they promoted and coordinated adherence to the priorities by stipulating that only projects designated as Urgent Public Health studies would be eligible for NIHR support. While other studies were not forbidden, this facilitated a concentration of resources, including redeployment of research staff who had previously worked in other areas, which was one of the reasons that the RECOVERY trial recruited its first 1000 patients within 16 days [3, 39]. As Wyatt et al. noted, “Supporting and facilitating such research has been made possible by the widespread reorganization of the NHS’s existing embedded research infrastructure” [102].

#### Priority-setting and reducing waste

The intense prioritization in the United Kingdom, especially in the early months of the pandemic, led to concerns from some researchers and patient groups that important topics were excluded, and that the prioritization process was not entirely transparent [10, 102]. Nevertheless, a key lesson is the recognition that the centralized prioritization meant that the United Kingdom avoided much of the duplication and waste noted in the Background section, and which occurred in many systems across the globe [1, 34–42]. Such waste occurred in Australia, Canada, the United States and many countries in Europe, where there were many small, underpowered trials that were sometimes not even completed. For example, despite the progress in coordination, it still proved difficult in a federal country such as Canada to achieve a national prioritization of research gaps that brought together the subnational units and that consistently utilized the existing research entities [107]. The issues of prioritization and coordination inevitably involved the health and HRSs working together, which was more feasible in the United Kingdom.

A major challenge developed that became labelled “hype-based medicine”, that is, the demand for unproven treatments, driven especially by ill-informed parts of the media and political leadership [38, 39]. One aspect of this was the unstructured but widespread patient demand for treatments such as hydroxychloroquine (HCQ), with which some clinicians acquiesced, and another was the explosion of small, duplicate trials of such drugs as certain clinicians, sometimes with very limited experience of research, desperately searched for an effective cure whilst coping with patient pressure [1, 36–39]. Particularly across Latin America, including Brazil, another version of the problem was called “populist treatment, instead of an evidence-based treatment” and involved ivermectin [108]. The United Kingdom was better equipped than most countries to resist these pressures because of the unified national healthcare system and the centralized research prioritization that could be strongly implemented through the integration of the NIHR into that healthcare system. There were overlapping advantages in terms of both the provision of care and conducting research to identify treatments that might be effective. For example, in their letter to clinicians in the NHS in April 2020, the chief medical officers of the United Kingdom not only encouraged them to include patients in the priority trials of repurposed drugs, but also strongly discouraged the use of off-licence treatments such as HCQ outside of trials [10, 41, 109].

#### Wider prioritization issues including considering the needs of diverse communities

Prioritization played a role in New Zealand’s broadly effective COVID-19 research response. Two of the three clinical trials of possible therapies for COVID-19 initially funded by the HRC and the Ministry of Health were international platform studies [110]. This perhaps best reflected the inevitably small size of the New Zealand health research sector, even before the policy successes in controlling the virus meant that there were very few cases. Other COVID-19 studies prioritized for funding from various programmes in New Zealand often focused on issues of particular local relevance, such as the needs of the Indigenous Māori community during the pandemic, and the use of genomic data to understand the spread of the disease through the population, to the small extent it occurred [110]. The New Zealand section of Additional file 1 also describes how key priorities consistent with the HRS vision and guiding principles [70] not only included topics of most immediate concern, but also increasingly covered research on long-term issues of reducing health inequity that COVID amplified for Indigenous peoples, and ensuring such research was Māori-led [110–112].

The COVID-19 research programme in NSW displayed somewhat similar priorities, including reflecting the 2018 strategy’s focus on meeting the needs of the First Nation communities [83]. The COVID-19 programme selected several projects related to the needs of Aboriginal communities and other issues of local relevance including working with long-established partners on projects such as genomic tracking, sewage surveillance and COVID-19 transmission in schools [113].

Across countries addressing COVID-19 issues was the prime priority-setting focus during the pandemic. However, some researchers who made rapid progress, such as those in Germany who developed the BioNTech mRNA vaccine, soon started considering how they could use the advances to make breakthroughs in other areas [114]. Furthermore, in relation to addressing the concerns of Indigenous and other minority populations, recognition of the historical factors explaining why such populations were potentially at greater risk during the pandemic helped inform the need for prioritization to address concerns of the communities. In addition to the examples above from New Zealand and NSW, some prioritization of the needs of such communities was included in general rapid COVID-19 calls including by the CIHR [115]. There were also specific calls, including as part of the APPRISE initiative in Australia in March 2020 [116, 117], and a CIHR call in September 2020 [118].

The prioritization of this issue in the United States was particularly complex. For example, the need to address the early failures in testing in the United States led to Congress authorizing a very large budget for the NIH to prioritize the Rapid Acceleration of Diagnostics (RADx) Initiative. It was set up initially in 5 days, but it took longer to implement [119]. One of its large programmes, the RADx Underserved Population Initiative, announced its first funding support for 32 institutions in September. It focused on a wide range of groups disproportionally affected by the pandemic. These included American Indians/Alaskan Natives, but also African Americans, older adults, pregnant women and the homeless and imprisoned. The programme aimed to “understand COVID-19 testing patterns better among underserved and vulnerable populations; strengthen the data on disparities in infection rates, disease progression and outcomes; and develop strategies to reduce these disparities in COVID-19 testing” [120].

In countries such as the United Kingdom, attempts to understand the nature of the disproportionate impact of COVID-19 on ethnic populations that nevertheless arose highlighted some of the detailed issues that still needed to be explored: “Prioritising linkage between health, social and employment data will be essential in building a complete picture of ethnic differences in COVID-19 risk and outcomes” [121].

Across countries, most major issues around the achievements when prioritization was successfully applied, or the waste arising from the lack of prioritization, related to the question of clinical trials testing drugs that could possibly be repurposed as therapies. Separately from the national prioritization of clinical research, Additional file 1 reports examples of researchers who did not wait for formal prioritization exercises but used their own previous research (and sometimes existing funding) to rapidly identify COVID-19-related topics and conduct extremely valuable research.

Researchers who had been developing new approaches to vaccine development for years immediately began to develop COVID-19 vaccines in January 2020 [12, 13, 18, 103, 114]. In Lesson 6, Capacity, we elaborate on the role of such researchers who were ready to respond to an emergency by immediately prioritizing the aspect of the impending crisis where they could potentially make a difference based on extensive previous work, which often reflected their own creative thinking (and that of many others). But here it is useful to note that the trials of the Oxford vaccine were designated as one of the first six Urgent Public Health studies in the United Kingdom, and thus prioritized to receive NIHR support [106]. OWS in the United States always planned to adopt a prioritization approach to identify the vaccine candidates it would support through late-stage development, advanced purchases and boosting manufacturing capacity [59]. It chose Moderna as one to support in development and manufacturing, and BioNTech/Pfizer with manufacturing [97]. Therefore, the pandemic response provided powerful lessons about the value of combining long-term space for researchers to develop their own priorities with mechanisms to allow various national-level stakeholders to select priorities on which to target their funding support—especially during a crisis.

### Lesson 3—Ethical approval: the ability to accelerate ethics and protocol approvals and to enhance data access and sharing increased the speed and efficiency of research production

According to Glasziou et al. (2020), one of the positives for research during the pandemic was the “expedited governance and ethics approvals of new clinical studies” [34]. There were various examples of this, but working with relevant authorities to ensure that ethical and regulatory approval was secured in good time was a particular feature of the acceleration of the development of vaccines such as Oxford/AstraZeneca. Such acceleration was facilitated because the urgency of COVID-19 research meant these studies went to the head of any of the queues that usually existed in research processes [17, 18, 121]. Similarly, for RECOVERY it took just 7 days from protocol finalization to approval, and just 2 more days to start patient recruitment [3, 39]. Furthermore, a very short protocol was used, which was markedly different from what had become the norm, especially in the United States. This assisted recruitment during a pandemic crisis [1, 40, 41]. REMAP-CAP had equally rapid approval in the United Kingdom, but a mean of about 3 months across European Union (EU) countries [39].

In the United Kingdom, and to some extent in BC, aspects of health data access, linkage and sharing for COVID-19 trials were also accelerated, with again a lesson that these processes can be accelerated where the situation and/or resources permit [1, 3, 93, 123]. RECOVERY, for example, was able to harness over 25 different datasets, including through repurposing of the recently created NHS DigiTrials, which minimized the burden for patients and staff [123]. Having the NIHR embedded in the healthcare system greatly assisted the data access and linkages [1, 3]. By contrast, despite some progress, difficulties remained in BC [93], and in countries such as Australia where Bowen et al. reported that the challenges facing platform trials there included the requirement for governance approvals at each site [101].

In the Australian state of NSW, the evaluation report of the NSW COVID-19 research programme also described how delays had occurred in site-specific approvals. However, the work stream on such issues within the comprehensive COVID-19 research programme (see Additional file 1) addressed the delays within NSW. Various other steps were also taken in the work stream on administrative processes to facilitate progress, and the evaluation reported that a pathway had been created “for efficiencies in future emergencies and business-as-usual procedures” [113].

In Brazil, there was recognition that the urgency of the pandemic meant that the processes of research ethics had to be accelerated. COVID-19 protocols received priority from the National Commission for Research Ethics to which they were sent, if necessary, from local levels within the system. The commission gave approval to 501 COVID-19-related study protocols by 25 June 2020 [124].

### Lesson 4—Evaluation: the substantial and immediate benefits from rapid (but expensive) research progress provide enhanced opportunities and need for impact assessment

The impact of the research on repurposed drugs, vaccines and other issues often occurred much more rapidly than usual [17], and assessments of the lives saved have been undertaken within months of the research being conducted, or even concurrently [2–8, 125]. Researchers from the UK RECOVERY team were some of the first to start identifying ways to assess the impact of their research. Perhaps this was because not only had their findings rapidly made an impact in terms of reduced mortality [2–4], but also the researchers might have been comfortable with the idea of considering the impact of their health research in such terms because the UK NIHR had pioneered research impact assessment as part of the overall strategy [73, 74]. Building on an initial detailed estimate with wide margins made by members of the RECOVERY team and others for the period from July to December 2020 [2], the NHS estimated by March 2021 that the use of dexamethasone had saved 22,000 lives in the United Kingdom and possibly a million worldwide [4].

Estimates of the deaths averted by vaccines included one by an international research leader from Brazil, Cesar Victora, and colleagues, of over 40,000 people aged 80+ in Brazil by May 2021 [8]. Other studies with a wider perspective estimated even larger numbers, including about 470,000 people aged 60+ across 33 countries in WHO’s European Region by November 2021 [7], and 140,000 in the United States in the first 4 months [5] and over 1 million by December 2021 [6].

While these vaccine studies did not conduct full research impact assessment, for example, in terms of identifying the specific cost of the research linked to the impacts, the relatively small number of successful vaccine development programmes would mean that some attribution to particular research programmes could be made in very broad terms. Looking forward, the much more rapid research translation during the pandemic would assist impact assessments and enhance the rate of return from research expenditure by greatly reducing the usual years of delay [17]. Such usual delays not only complicate assessments but also depress the rate of return, because delayed benefits, occurring for example in the oft-quoted 17 years after the research investment, are much less valuable than almost instant benefits [84].

The evaluation of the NSW COVID-19 research programme included the early stages of a formal research impact assessment [113]. In part, this assessment was organized using the Framework for Assessing the Impact of Translational health research, itself developed earlier in NSW [126], and in part informed by NIHR development of impact assessment [73]. The evaluation described early impact and a plan for a longer-term assessment, in particular of the sewage surveillance programme. That study validated the methods used by Sydney Water, with which there was a long-standing research partnership. The findings enabled NSW Health “to target messaging and testing to high-risk areas” and were also used to manage border restrictions [27, 113]. Longer-term assessment was planned of the benefits to health and the economy in NSW of being able to perform the testing in high-risk areas and translate findings to policy-makers and the public [113] (see Additional file 1).

UK studies are beginning to attempt to consider the impact of the research from specific institutions. One study of the Oxford Biomedical Research Centre, which contributed to both the RECOVERY trial and the Oxford AstraZeneca vaccine, adopted an approach that was particularly relevant for the pandemic [127]. First, it considered the benefits (or “option value”) resulting from having research capacity in such centres that could be rapidly deployed to address the crisis [127]—the role of such capacity was also considered in a later paper [128]. Second, it proposed that any resulting contribution to accelerating research that was used to reduce the need for economically damaging pandemic countermeasures, would provide a high rate of return [127]. The latter point was also due to be adopted in the planned NSW evaluation [113]. Another UK study involved a detailed analysis of the impact of having a largely NIHR-coordinated research infrastructure embedded in a major London hospital trust. This coordinated infrastructure was able to facilitate the rapid redeployment of research staff—some to provide extra clinical staff in the COVID-19 crisis, and some to contribute to the COVID-19 research that made such rapid progress in the United Kingdom [129]. This paper, therefore, highlighted the degree of effort and reorganization that was required to achieve the benefits from having the research infrastructure that could respond flexibly to the pandemic.

Despite widespread public admiration for the achievements of science during the pandemic, the previously noted concerns about the level of waste [1, 34–42] were exacerbated by recognition of the damage the pandemic wrought on many economies. These factors coincided with the considerably increased resources provided for COVID-19 research, as elaborated below, and might lead to increased recognition of the desirability of conducting COVID-19 research impact assessment in order to defend or justify the research budget and sustain HSRs going forward [127].

### Lesson 5—Finance: unprecedented (but uneven) funding; public, for many pandemic topics; private, for development of vaccines and therapies; collaborative, to help achieve major successes; but widespread concerns about wasted resources

In contrast to capacity-building, securing funding is probably the HRS function where it is possible to make most changes to pre-existing systems and approaches in a short timescale—both positively and negatively. But analysing the unprecedented rapid expansion, and/or concentration, of funding on one disease presents various challenges given the many different types of funders, the different ways the funding could be allocated, the timescales involved and the levels of collaboration across funders and researchers.

One approach has been to focus on funders and attempt to identify the total number of research projects they funded and the amounts they allocated. In an inevitably challenging exercise, with Chinese data particularly difficult to secure, a UK and Canadian team created a global living mapping review of COVID-19 funded research projects [130]. Focusing on public and philanthropic funding, it found that the United States, the United Kingdom and Canada had the largest numbers of funded projects, with the same order for the amount of funding, except that in some editions Germany provided the third highest level. In the edition published in July 2021, the database contained “10,608 projects, funded by 201 funders, taking place across 142 countries representing an investment of at least $4.7 billion” [130]. It included newly funded research projects, and repurposed ones, across all disciplines in what it described as “an unprecedented research response, demonstrating exceptional examples of rapid research and collaboration”.

Also, in July 2021, a different team published a larger figure for total expenditure, despite focusing solely on funding for vaccine R&D. It reported: “The US and Germany are by far the largest investors in vaccine R&D, followed by a relatively small number of other (mostly) high-income countries, with China being the exception. Public funding represents the vast majority of the data collected (90.69% of the USD 6.6bn tracked)” [131]. It found that pharmaceutical companies in general had not disclosed specific figures for R&D spending related to COVID-19. Nevertheless, the recipients of this R&D vaccine expenditure were overwhelmingly private companies, though the figures did include the AstraZeneca/Oxford University partnership [131]. The Coalition for Epidemic Preparedness Innovations (CEPI) represented a further example of collaboration in that this global funder of vaccine R&D received its resources from national governments and philanthropic sources [103]. It was also argued that advanced purchase agreements signed by countries to purchase vaccines prior to regulatory approval also represented a type of R&D contribution because they “could be understood as an additional incentive that reduces business risk in the R&D stage” [131]. The expenditure on the advanced purchases was far larger than for the standard R&D.

To illustrate various points most relevant for our lessons, on Table 3 we list examples of types of specific COVID-19 research funding announcements made within different HRSs. The increased funding was uneven, both between countries and over time. Canada created a large federal government funding pool for COVID-19 research which PM Trudeau announced in March and April 2020 [90, 132]. As noted in Lesson 1, these funding statements provided an element of coordination because they brought together the public funding being provided to new organizations, such as the Canadian COVID-19 Genomics Network, and established organizations including CIHR, the Canadian Immunization Research Network, and the Strategic Innovation Fund, which collaborates with the private sector. In Canada, as in all federal countries, there was also funding from the subnational governments. The combined effect of the federal and provincial funding in BC was noted in the evaluation of the province’s research response to COVID-19: “The rapid and substantial infusion of research funding to established institutions and researchers was perceived by many stakeholders as the most important contributor to success of the research response” [93].

**Table 3:** Examples of types of funding announcements illustrating various points most relevant for the lessons (e.g. rapid call, minorities, other topics)

Date	Country/funder	Topic/title of programme	Amount in local currency	USD	GBP	EUR
*Combined spending plans*						
March 2020	Canadian federal government [132]	Press release: Canada’s plan to mobilize science to fight COVID-19	Can$ 275 million	200 million	162 million	180 million
April 2020	Canadian federal government [90]	Press release: PM announces new support for COVID-19 medical research and vaccine development	Can$ 1billion+	0.73 billion+	0.6 billion	0.65 billion
*Rapid/early funding*						
Feb 2020	UK: NIHR/UK Research and Innovation (MRC) [133]	Novel coronavirus research: (1) vaccines and therapeutics; (2) diagnostics and epidemiology, spread, containment, basic knowledge	£20 million	25 million	20 million	22 million
Feb 2020	Canada: CIHR and other research councils [115]	Canadian 2019 Novel Coronavirus (COVID-19) Rapid Research Funding	Can$ 7 million (ended up Can$ 55 million)	5 million (40 million)	4 million (32 million)	4.6 million (36 million)
Feb 2020	New Zealand: HRC/Ministry of Health [134]	Government agencies launch rapid research response to COVID-19 threat	NZ$ 3 million	1.9 million	1.5 million	1.7 million
April 2020	Brazil: National Council for Scientific and Technological Development [135]	COVID-19 treatment, vaccine, diagnosis, pathogenesis and natural history of the disease, disease burden, attention to health, prevention, and control	R$ 49 million	9.2 million	7.4 million	8.3 million
*Initial funding—indigenous/minority populations*						
March 2020	Australia: Paul Ramsay Foundation [116]	Support for APPRISE COVID-19 research with First Nations Peoples (part of Foundation’s A$ 9 million initial COVID programme)	A$ 2 million	1.3 million	1.2 million	1.2 million
Aug 2020	New Zealand: HRC [111]	Māori-led part of 2020 COVID-19 Equity Response (Total NZ$ 8 million)	NZ$ 3 million	1.9 million	1.5 million	1.7 million
Sept 2020	Canada: CIHR [118]	Indigenous COVID-19 Rapid Research Funding Opportunity	Can$ 2 million	1.5 million	1.2 million	1.3 million
Sept 2020	USA: NIH [120]	Rapid Acceleration of Diagnostics (RADx) Underserved Populations (first allocations to 32 institutions—part of US$ 1.5 billion RADx initiative begun in late April 2020)	US$ 234 (up to US$ 512 million by Nov 2021) [136]	234 million	189 million (414 million)	210 million (460 million)
*International/networks*						
March 2020	UK: government [137]	Press release: PM announces record funding to find a coronavirus vaccine—donation to CEPI	£210 million	260 million	210 million	233 million
Oct 2020	Germany: Federal Ministry of Education and Research [94]	Funding of 13 projects in the National Research Network of University Medicine (network had been established in spring 2020)	€150 million	167 million	135 million	150 million
*Subnational organization*						
March 2020	Brazil: Rio de Janeiro state government [135]	Rio de Janeiro State Research Support Foundation—COVID-19 research programme	R$ 24.6 million	4.6 million	3.7 million	4.1 million
April 2020	Australia: NSW Government [113]	NSW Health COVID-19 Research Program—various streams	A$ 25 million	16.7 million	13.5 million	15 million
*Public/private sector collaborations*						
May 2020	US federal government [59]	Operation Warp Speed: public (multi-agency)/private collaboration: develop and manufacture new vaccines and drugs	US$ 10 billion (ended up higher)	10 billion	9 billion	8.1 billion
Sept 2020	Germany: Federal Ministry of Education and Research [138]	Funding to BioNTech (part of €750 million to three companies) to support development and production capacity for COVID-19 vaccines	€445 million	496 million	401 million	445 million

Currency equivalents as at end of May 2020

In many countries the major health research funding bodies launched rapid funding calls shortly after the outbreak of the pandemic and continued to launch calls throughout the pandemic. At various times there was recognition of the need for research programmes to identify and promote the particular needs of minority communities, especially Indigenous populations (outside of Europe). There was also funding recognizing the importance of networks as well as various forms of collaboration. These are just a few of the key topics on which there were specific funding announcements, and Table 3 provides just some examples of such categories to illustrate the range of funding provided in our countries.

In the United States, OWS’s very large spend contributed to public/private collaborative development of vaccines (such as Moderna and Johnson & Johnson) and accelerating therapeutics, described in detail in Additional file 1 [59, 96, 97, 139]. The NIH, and later BARDA, played a major role not only in funding early research to develop the mRNA technology, but also in the funding and conduct of the Moderna trials [16, 140–142].

As previous analysis had shown, trials for vaccines or new drugs become increasingly expensive as the later phases are increasingly larger. This creates financial risk, and therefore, traditionally, vaccine/drug developers have generally preferred to have confirmed early evidence strongly suggesting effectiveness before moving on to the larger trials necessary to confirm effectiveness and checking for rare side-effects, as is always required for regulatory approval [17, 143]. The provision of greater resources via OWS was undoubtedly a key factor in accelerating vaccine and drug development. It enabled some of the usual phases of the development to be conducted in parallel [17, 97, 143, 144], as had been previously identified as a potential way of accelerating the development of drugs and vaccines [84]. Similarly, the unprecedented level of preordering at risk by the OWS programme, and others, not only reduced risks to companies, but also meant that enormous amounts of money were being used to ensure that a supply of vaccines, and in some cases drugs, would be available for distribution as soon as they received regulatory approval [59, 96, 97, 143].

The need for new and additional COVID-19 programmes evolved as the pandemic continued. For example, the allocation to the CIHR of approximately Can$ 150 million as part of the initial two overall allocation announcements from the Canadian government [90, 132] ended up as an amount spent of over Can$ 400 million over 39 competitions on COVID-19 research.

Despite the unprecedented levels of funding, some researchers still faced funding challenges. There was some public funding from both federal and state agencies in Brazil [135], but it was the researchers there, out of our seven HRSs, who faced the greatest difficulties securing finance for COVID-19 research [20, 145]. Elsewhere, even some of the COVID-19 academic research that was eventually the most successful could face challenges. For example, the work at Oxford University on what became the Oxford/AstraZeneca vaccine initially faced considerable battles in securing financing before eventually partnering with a commercial company. The Oxford team also increasingly secured some larger grants in addition to OWS support, including some funding from CEPI, to which the UK Government had made a major contribution [18, 137]. Somewhat similarly, in Germany, BioNTech had received government and EU funding in the early stages of developing an mRNA vaccine but needed much more capital for the COVID-19 vaccine and partnered with the US company Pfizer [114], and later received a large grant from the German government for late-stage development and manufacturing [138].

Finally, the picture was also complicated by two further factors that highlight the importance of taking an overall perspective, and also the importance of coordination. Specifically, despite the Brazilian HRS facing cuts and financial challenges both before and during the pandemic [20, 145], the strength of the research capacity in Brazil had been built up over many years, and the coordination of the clinical research through the Coalition COVID-19 Brazil initiative resulted in some important contributions, for example in the testing of repurposed drugs described above and in Lesson 7 [20, 21, 88, 146].

There was always likely to be a risk that increased financing provided during the crisis to conduct research to develop countermeasures against the virus would be accompanied by an increase in some of the resources being wasted. As noted above, this problem has been widely highlighted as occurring in many systems [1, 34–42], and the FDA’s analysis was based on worldwide registers of COVID-19 trials [35]. This emphasizes the interrelationship between the lessons from HRSs’ response to COVID-19, because where the coordination was strongest, namely in systems such as the NIHR in the United Kingdom, there was less danger of duplication and multiple underpowered small studies.

### Lesson 6—Capacity-building: important contributions came from mobilization of capacity developed over years to conduct primary and secondary research, enhanced interdisciplinary cooperation and clinical research integrated in healthcare systems

In all seven countries, the existing research capacity was used to make significant contributions to the production and utilization of a wide range of valuable research. Often a noticeable feature of the response was the long period over which key elements of such capacity had been built up, and the wide range of disciplines that sometimes collaborated. However, the rapid progress with COVID-19 research production and utilization also depended on the continuing pioneering development of capacity.

#### Rapid mobilization of vaccine research capacity built up over many years

The rapid development of vaccines during the pandemic provides a prime example of research relying on the many earlier years in which the capacity to conduct basic or discovery research had slowly advanced, and while this took various forms, the mostly publicly funded early work in universities and public laboratories was crucial [11, 12, 17, 18]. The years of painstaking, sometimes discouraging, research into the potential for using mRNA for vaccines and drugs had been conducted by scientists at various institutions in the United States. These included universities (notably the University of Pennsylvania, where Katalin Karikó—who later also joined BioNTech—and Drew Weissman had made important progress), NIH laboratories, and companies in the United States such as Moderna working with the NIH [11, 12]. Similarly, in Germany in the years prior to the pandemic, medical academics Uğur Şahin and Özlem Türeci received funding from the government-backed German Research Foundation (DFG) [147] and the EU [114] for development of their work on mRNA at the University of Mainz. This contributed to the creation of their company, BioNTech [11], but they needed considerable further funding as well, including venture capital [114].

Progress on developing mRNA technology was facilitated through an originally unrelated stream of lipid nanoparticle technology research which provided a way of delivering the mRNA to cells [11, 13]. Of many contributions to this, the research of Pieter Cullis in Canada, at UBC and spin-off companies including in Vancouver, BC, is highlighted in particular [11, 13]. BioNTech and Moderna (in conjunction with the NIH) were ready to start working on what would become the first-ever approved vaccines using this pioneering new technology immediately after the news of the outbreak of a new virus in China started filtering out and the genetic sequence was released [11–13].

Many years of publicly funded vaccine development at the Jenner Institute, University of Oxford (UK), had resulted in a platform to enable the team to respond more rapidly than ever before to a new virus, the unknown Disease X. Severe acute respiratory syndrome coronavirus 2 (SARS-Cov-2) turned out to be Disease X, and Sarah Gilbert, Catherine Green, Teresa Lambe and colleagues immediately started using their newly created platform as soon as the genetic sequence was released [18, 121, 148]. Established capacity in Australia and the United States to conduct animal trials was also important in the rapid development of vaccines [17, 149, 150], as was high-quality capacity to conduct human trials in countries including Brazil and South Africa [18, 20].

#### Mobilizing and extending wide-ranging primary research capacity sometimes linked to networks

Existing strong capacity was also rapidly mobilized in institutes and laboratories linked to major hospitals. Researchers across Europe rapidly developed the reverse transcription polymerase chain reaction (PCR) test, led by Christian Drosten from the Berlin Institute of Virology at Charité Hospital. Publications from this and other studies using the test are described in Lesson 7 [151–155]. Similarly, researchers at the Peter Doherty Institute in Melbourne rapidly conducted studies [85–87, 156], in one of which the team suggested that a reason for the success was the extensive clinical experience in the laboratory [156]. We noted above how capacity at the institute was rapidly mobilized to address priorities in Australia through the APPRISE platform, and research system investment in such networks proved to be of considerable value [86, 116]. This was also the case with immunization research in Canada [157].

In the United Kingdom, in March 2020, researchers led by Sharon Peacock, with support from the UK Government’s Chief Scientific Adviser Patrick Vallance, rapidly built the COVID-19 Genomics UK Consortium. It was based on existing strengths in areas such as pathogen genomics and the network of specialist academic facilities working with public health agencies and the NHS, with the MRC-funded and readily accessible cloud bioinformatics infrastructure being a particularly important facilitator [103, 158, 159].

Many university researchers used their capacity in early research to attempt to develop new drugs to treat COVID-19. For example, several teams of established academic researchers, funded (in part) by the CIHR, collaborated to make some progress developing possible new antiviral treatments at the University of Alberta, Canada [160, 161]. Starting earlier through pre-pandemic research funded by the National Institute of Allergy and Infectious Diseases (NIAID) and others, a team at Emory University, Georgia, USA, had invented the antiviral molnupiravir that was then developed and tested as a COVID-19 therapy by Ridgeback Biotherapeutics and Merck Sharp & Dohme (and authorized in late 2021) [162–164].

Researchers from the full range of scientific disciplines made useful contributions to the COVID-19 research response. Examples described in Additional file 1 include a study led by anthropologists in which volunteers who had participated in vaccine trials in the United States were interviewed to better understand vaccine motivation in an attempt to inform vaccine promotion [165].

#### Mobilizing and extending secondary research capacity and also capacity to use evidence

In HRSs including in Australia and Canada, existing strong capacity that had been created over many years in secondary research in the form of traditional systematic reviews, and the much more recent living systematic reviews, was rapidly leveraged and extended [24, 25, 107, 166]. In Australia, the particular strength in pioneering living systematic reviews was being extended just prior to the pandemic to include the development of living guidelines [166]. In Canada, the existing capacity to conduct systematic reviews and produce evidence for policy-makers seemed widespread. There was an ability for further training to be provided, for example by the Strategy for Patient-Oriented Research (SPOR) Evidence Alliance [167], and international recognition through the ability of COVID-END to create and lead a multicountry expert consortium [168]. (These examples are elaborated in Lesson 9.)

In Germany, one of the 13 projects of the "Network University Medicine", the COVID-19 Evidence Ecosystem (CEOsys), established a national evidence network on COVID-19 led by the Institute for Evidence in Medicine, for Cochrane Germany, at the University Hospital Freiburg. The evidence ecosystem involved 20 university hospitals and several nonuniversity partners coordinating to identify and evaluate scientific findings on a range of COVID-19 issues and produce a series of interdisciplinary living evidence synthesis. These were then used to produce living guidelines as described in Lesson 9 [169–171].

At the subnational level in NSW, the capacity-building set out in the 2018 strategy both for researchers to collaborate with users and for NSW Health staff to use evidence, proved to be extremely valuable in the pandemic, and was taken further—see Lesson 9 [83, 113].

#### Interdisciplinary research

We identified examples in several countries, including Brazil and Canada, where important aspects of research progress during the pandemic relied on scientists from different disciplines working together. In Brazil, in mid-March 2020, multidisciplinary collaboration between universities/research institutes/companies in Sāo Paulo developed and produced molecular diagnostic tests [172].

Ontario, Canada, was one of many places where teams conducted wastewater surveillance, including to detect outbreaks in so-called hotspot communities. A review performed by Public Health Ontario showed how such studies had become widespread and were being used in interdisciplinary research [173]. Modellers also estimated surges in cases and intensive care admission using funding from federal (CIHR), provincial and institutional (St. Michael’s) levels [174].

#### Mobilizing and organizing clinical trial capacity in health systems

There were useful attempts during the pandemic to build on and coordinate the considerable research capacity that existed in healthcare systems, for example in the United States, BC, and in Brazil, where the rapid creation of Coalition COVID-19 Brazil was described above [20, 21, 88, 146, 175]. Despite their achievements, these various efforts inevitably faced challenges [88, 93, 100, 176].

In the debate about how the RECOVERY trial made such rapid progress in the United Kingdom, the existing research capacity infrastructure of the CRN throughout UK hospital trusts was an extremely important element [1, 3, 10, 39, 102, 103, 177, 178]. This infrastructure meant not only that there were clinical researchers across the entire country who were ready to participate, but also that considerable progress with various governance issues discussed above had already been made. This was contrasted with Australia, Canada and overall in the United States, where analysts reported such infrastructure did not exist in terms of one coherent national clinical research network embedded in the healthcare system, and research progress was slower [40, 99–101, 179]. Many of these authors thought the NIHR/CRN could provide lessons for reforms in their own systems.

There was considerable racial disproportionality in COVID-19 clinical trials in the United States, with underrepresentation of minority groups, despite the existence of many strategies to increase enrolment of diverse populations [180, 181]. According to FDA Acting Commissioner Janet Woodcock and colleagues, problems facing US trials included insufficient diversity as well as slow enrolment. Both could be addressed, they suggested, by integrating research into community practice, the source of healthcare for many Black and other Americans underrepresented in trials [181].

### Lesson 7—Knowledge production: accelerating research production (new vaccine platforms, mobilized capacity, adaptive platform trials) produced results—but problems for policy research; rapid publication of findings became essential but led to dangers

A major lesson from the pandemic is that the three components covering knowledge production and use, namely components 7, 8 and 9, have at times overlapped as some of the traditionally sequential phases of research development and translation into products have been accelerated and conducted in parallel [17, 84, 97, 143]. Additionally, the increasingly valued approach within HRS of the co-production of knowledge had been noted (and encouraged) in the WHO evidence synthesis [66], and in some cases has played an important role in the pandemic response [30, 51, 182]. Nevertheless, it is still appropriate to start with knowledge production, the seventh component of the overall HRS framework, while highlighting some examples of valuable overlap; for example, the rapid knowledge production in the development of vaccines resulted in many important papers  which are described in Lesson 8 on product development.

Additional file 1, the Background section and sections below illustrate the enormous amount of research conducted on a wide range of COVID-19 topics. In terms of research outputs, the results were highly variable, ranging from some rapidly conducted and published studies whose highly cited papers generated immediate and worldwide attention on a range of COVID-19-related topics, to many small, duplicate studies that would be unlikely to produce meaningful results [34, 35]. Between these ends of the spectrum many studies were published that made some contribution to the body of knowledge. One feature of many of the more high-profile papers is that they involved collaboration between teams from different institutions, sometimes different countries [9, 14–16, 19, 151, 183].

Knowledge was extremely rapidly produced on sequencing, diagnosing and isolating the virus, through the immediate mobilization of capacity. In some cases, research teams themselves mobilized in their existing international collaborations, such as the one across China and in Sydney in which Yong-Zhen Zhang and Edward Holmes and colleagues sequenced the SARS-CoV-2 genome [103, 183]. The full paper was submitted to *Nature* on 7 January 2020 and published on 3 February, receiving over 4000 citations by 31 December 2021 [183]. Holmes received the 2021 Australian PM’s Prize for Science for his “transformative role in the scientific response to COVID-19, and his groundbreaking research into the evolution of viral diseases” [184].

In a further paper, Zhang and Holmes [185] also noted that their initial release of the sequence had facilitated the immediate mobilization of research teams such as those led by Drosten in Germany and colleagues mostly in Europe who developed the PCR test extremely rapidly (by 16 January 2020) [151]. Drosten’s team then worked with the open access journal *Eurosurveillance* to successfully facilitate equally rapid appropriate peer review, which in turn led to publication on 23 January 2020 [151, 153]. A further feature of this rapidly mobilized research by some institutes linked to hospitals was that it could result in the production of a series of important, highly cited papers about the virus. For example, Drosten collaborated with various colleagues over several publications beyond, and drawing on, the PCR test, including a very rapidly produced case report letter published in the *New England Journal of Medicine* (*NEJM*) on 30 January 2020, about the transmission of COVID-19 infection by an asymptomatic contact in Germany initially identified on 27 January [186]. It was cited over 2000 times by 31 December 2021. Another rapid paper published in *Nature* on isolating the virus from patients with mild symptoms was cited over 3000 times by 31 December 2021 [155]. Similarly, teams at the Peter Doherty Institute in Melbourne Hospital rapidly produced papers, including one reporting the first isolation of the virus outside of China [156] as well as the previously noted research letter in *Nature Medicine* that was cited over 500 times by the end of 2021 [87].

#### Adaptive platform trials

The pandemic increased recognition of the considerable advantages from using adaptive, multi-armed platform trials (with simple protocols) for identifying known therapies that could be safely and effectively repurposed to treat COVID patients. Platform trials mean, of course, that the effectiveness of multiple drugs can be trialled simultaneously, and their adaptive nature means that additional drugs can be added as the initial ones are found to be effective or not effective. The advantages were acknowledged by research leaders in the United States such as Anthony Fauci, head of the NIH’s NIAID [40], and by those who had successfully led the RECOVERY platform trial in the United Kingdom [3, 39] that resulted in a range of important publications in leading journals [9, 187, 188].

The international REMAP-CAP platform trial had sites across all seven of our countries bar Brazil prior to the pandemic [189, 190]. The collaborators held a meeting on COVID-19 on 23 Jan 2020 and by 3 March had adapted the trial to add COVID-19 treatments to which internationally it started recruiting patients on 9 March [39, 189]. The project soon became an early Urgent Public Health priority study in the United Kingdom, which led to rapid recruitment in the United Kingdom, and later 65% of the patients in the international trial were recruited in the United Kingdom [104]. Publishing in leading journals *JAMA* and *NEJM*, it also contributed to the bodies of evidence showing the effectiveness of corticosteroids [22, 191] and interleukin 6 (IL-6) receptor antagonists [192, 193], but did so to a much smaller extent than the RECOVERY trial. Leading members of the REMAP-CAP team described the frustrations in countries outside the United Kingdom, such as the United States and across Europe, with delays in recruitment and/or setup [39, 100], and, as noted, leaders of the ASCOT trial made similar comments [101]. Therefore, the platform trial design was of most benefit where combined with other features of the UK integrated health and research system described above.

Three other platform trials also showing the benefits of the approach in terms of producing important knowledge all involved researchers from Brazil and Canada, and in two cases from other nations in various combinations. The WHO’s Solidarity Therapeutics Trial was a large global platform trial with sites in many countries, including, as noted, Brazil and Canada, and with funding from many organizations including the CIHR [64]. The Brazilian contribution was coordinated by the Ministry of Health’s Oswaldo Cruz Foundation (Fiocruz) in Rio de Janeiro [194], and the Canadian arm by a clinician linked to UBC in Vancouver. Solidarity’s interim findings, published in *NEJM*, were on the first four repurposed drugs investigated and suggested that none of them was effective in treating hospitalized COVID-19 patients [64]. The TOGETHER platform trial was led by Pontificia Universidade Católica de Minas Gerais, Brazil, and McMaster University, Hamilton, Canada, funded by North American philanthropists and conducted originally in 11 clinical sites across Brazil. One of its arms studied the antidepressant fluvoxamine as an early treatment for COVID-19 for patients with a known risk factor for progression to severe disease. Published online in *The Lancet Global Health* in 2021, it showed that the treatment reduced the need for hospitalization in patients who, given the recruitment period, were primarily unvaccinated [195]. Reliable reports in autumn 2021 that another arm of TOGETHER would show no benefit from the use of ivermectin [196] were confirmed in the trials’ report in *NEJM* in 2022 which attracted considerable attention [197].

In the third combined platform trial, Brazil had the most sites in the Canadian public/philanthropy-funded Antithrombotic Therapy to Ameliorate Complications of COVID-19 (ATTACC) adaptive trial that combined with two other platform trials (REMAP-CAP and parts of the NIH’s ACTIV platform) in an international trial to which Brazil enrolled the second-highest number of patients overall after the United States. Published in *NEJM,* it showed in non-critically ill COVID-19 patients that an initial strategy of therapeutic-dose anticoagulation with heparin increased the probability of survival to hospital discharge [198]. (In the three platforms’ parallel trial with critically ill patients, REMAP-CAP took the lead, and most patients were enrolled in the United Kingdom, but for these patients the findings, also in *NEJM*, showed that the treatment was unsuccessful [199].)

#### Examples of policy-relevant research—achievements and challenges

Policy-relevant research was conducted in all countries, but here we focus on a few examples that illustrate key points. A set of papers from New Zealand also featured elements of regional collaboration in knowledge production on issues of interest to local policy-makers with whom there were some elements of co-production that are also discussed in Lesson 9. First, a paper using funding from the prioritization of local topics by New Zealand’s HRC, plus other sources such as the business ministry’s COVID 19 Innovation Acceleration Fund, used genetic epidemiology to reveal transmission patterns and dynamics of COVID-19 in the country, thus helping to quantify the effectiveness of public health interventions such as lockdowns [200]. This paper in *Nature Communications* was written by a team from various institutions mostly in New Zealand, but including some international authors, for example from the Doherty Institute in Australia. The paper was devised by Jemma Geoghegan, who wrote the initial draft with Edward Holmes (see above). They were also co-authors on a paper led by Tara Swadi, chief advisor on COVID-19 at the Ministry of Health. It analysed transmission of COVID-19 that occurred in a flight to New Zealand. This generated considerable international interest, as well being of local relevance [201].

Sarah Jefferies from the Institute for Environmental Science and Research in New Zealand was a co-author on both the above papers [199, 200]. With funding from the two relevant ministries, i.e. health and business, she and colleagues at the institute and elsewhere, including one colleague from the Ministry of Health, conducted a study that used highly complete prospectively collected COVID-19 case and testing datasets [202]. They claimed, “This is the first study to our knowledge to assess the impacts of national or subnational non-pharmaceutical intervention escalation and de-escalation decisions on the distribution, transmission patterns, and severity of COVID-19, and the attainment of an explicit national goal of COVID-19 elimination” [202]. The study led to clear findings that strikingly reinforced the value of the rapid-evidence-informed policy: “New Zealand's response resulted in low relative burden of disease, low levels of population disease disparities, and the initial achievement of COVID-19 elimination” [202]. The paper, published in *The Lancet Public Health*, attracted considerable attention. Jefferies and colleagues won an HRC medal for their research [182].

In Australia, a study on COVID-19 transmission in schools involved researchers from the NSW-based National Centre for Immunisation Research and Surveillance working with the state’s Ministry of Health and Department of Education [113]. It not only resulted in a publication in *The Lancet Child and Adolescent Health* that has received considerable international attention [203], but also informed decision-making [27].

While in the above examples the policy-relevant knowledge production was welcomed, several analyses of lessons from the COVID research response considered the challenges in producing evidence to inform policies about NPIs such as face masks where it was extremely difficult to conduct standard randomized controlled trials (RCTs) [38, 50, 53, 204]. Analysts highlighted the frustration of researchers such as Trish Greenhalgh and a team of international colleagues who, in an article in *The BMJ* in April 2020, had drawn on the evidence that was available on masks and called for support for mask mandates based on application of the precautionary principle [204], only to find that policy-makers in the United Kingdom initially seemed reluctant to act in the absence of the gold-standard RCT evidence [38, 50].

Van Schalkwyk and McKee also supported the approach of Greenhalgh et al., and highlighted the benefits of non-RCT study designs that were more appropriate for this type of issue in the pandemic [53]. These included a paper entitled “Face masks considerably reduce COVID-19 cases in Germany” [205]. In it, researchers from economics and business departments and institutes, mostly in Germany, used the synthetic control method to analyse what was in effect the natural experiment of local policy-makers introducing mask mandates at different times [205]. Its findings that face masks reduced the number of newly registered cases of COVID-19 by between 15 and 75% over a period of 20 days from their mandatory introduction were published in *Proceedings of the National Academy of Science,* and widely reported and cited in Germany and internationally [206, 207].

#### Accelerated publication

Much of the research of all types was not only conducted more rapidly than usual, but also published rapidly through a vast acceleration in the existing trends towards greater use of both open access publications, which sometimes could be published soon after submission, as above, and preprints [34]. Preprint registries became important vehicles for rapid dissemination of research, as highlighted by researchers such as Peacock, who had rapidly established the COVID-19 Genomics UK Consortium and who “recognised the importance of an open science culture” [158]. The dangers, however, include the important caveat that the research has been made public prior to peer review. Consequently, in relation to HCQ, for example: “Access to preprints has also led to irresponsible dissemination as flawed studies are picked up by the media”. Commentators noted that this happened, too, with some poor-quality papers that presented messages promoted by those opposed to NPIs [34, 41, 53].

With their findings such as those on the effectiveness of dexamethasone, the RECOVERY team went even further and announced the clear results in a press release [208]. This approach was criticized by some, but it was defended by the researchers who argued it was justified because so many people were dying—only 4 hours after the press release was published, the chief medical officers for the UK nations issued a recommendation for the therapy’s adoption [10, 36, 110]. Presumably, their confidence in the findings was linked to the known size and quality of the study, which had been selected as one of the first six Urgent Public Health studies to be allowed to draw on NIHR resources, and it had been conducted throughout the healthcare system. A preprint followed a week later, and within a month a preliminary version was published in *NEJM* and cited about 3000 times by the end of 2021 [9, 10].

### Lesson 8—New products: translation of research into new products to reduce mortality and morbidity often occurred at unprecedented speed and often reflected unprecedented levels of both public funding and public/private collaboration tackling the crisis

There are lessons from the way the trials and other research on vaccines, diagnostics, devices and therapies such as new monoclonal antibodies (mAb) rapidly led to new tools to tackle the pandemic.

#### Vaccines

The speed of new vaccine development was unprecedented. COVID-19 vaccines provide a strong illustration of the overall lesson described at the start of Lesson 7 about overlapping knowledge production and use. We have previously described various factors that contributed to the accelerated vaccines. These factors included historical ones about the years of development by teams (especially in Germany, the United Kingdom and the United States). Other factors occurred during the pandemic and included the prioritization of vaccine trials and acceleration of some governance functions (especially in the United Kingdom), the additional finance through public/private partnerships, and the consequently greatly accelerated R&D and advanced manufacturing conducted in parallel (especially in the United States and United Kingdom) [11–13, 17, 18, 59, 97, 103, 114, 122, 143].

The knowledge produced was described in a series of important publications in *Nature* and *NEJM* about mRNA vaccines from both BioNTech/Pfizer [14, 15] and Moderna [16, 140–142], and in *Nature* and *The Lancet* about the Oxford/AstraZeneca vaccine (with trial sites also in Brazil and South Africa) [19, 150, 209]. For their work on mRNA, Uğur Şahin and Özlem Türeci and colleagues including Katalin Karikó (who had joined them from the United States in 2013) won the 2021 annual German Future Prize for research achievements leading to marketable technological developments [147]. Karikó and her colleague from the University of Pennsylvania, Drew Weissman, have also won major international prizes for their key contribution of showing how mRNA needed to be modified [11].

According to Gilbert and Green, the Oxford team liaised early and regularly with the UK regulators, and supplied over 500,000 pages of data for the Oxford/AstraZeneca vaccine. For their part, the regulators looked at the data “just as carefully as they always do. But they started sooner, and put more people on it” [18]. There was considerable praise for the role of OWS in the United States and the Vaccines Task Force in the United Kingdom in rapidly ensuring supplies were ready so the vaccine rollout could begin as soon as the regulators approved the vaccines [97, 210].

There was also global interest in looking to diversify COVID-19 vaccine development. In September 2021, the Pan American Health Organization (PAHO) announced it had selected Fiocruz as one of two Latin American centres it would fund to develop and produce mRNA-based vaccines. It was selected because of its previous record on developing and manufacturing vaccines and having already made promising advances in developing an innovative mRNA vaccine against COVID-19 [211, 212]. On its website, Fiocruz stated that in relation to COVID-19, it would work with the Ministry of Health and that with a “tradition of more than 70 years in the production of vaccines, the Foundation has been committed to maintaining efforts in this field…and emphasizing the importance of the Unified Health System (SUS) as the basis for sustaining the development, production and future national distribution of a vaccine for the disease” [212]. Linked to the vaccine trial in Brazil, there was already a technology transfer agreement that meant Fiocruz had gone on to manufacture the Oxford/AstraZeneca vaccine in Brazil [20].

Accompanying the development of vaccines against COVID-19 there were many questions about the role such vaccines could play. *The Lancet* Commission on COVID-19 Vaccines and Therapeutics took a global perspective in its paper, “Operation Warp Speed: implications for global vaccine security” [139]. It highlighted issues such as “Evidence of community protection is crucial; it could inform government vaccination strategy and policy around ancillary protective measures”. The Butantan Institute in São Paulo, Brazil, had a technology transfer agreement with the Chinese company Sinovac Biotech to manufacture their vaccine following successful trials [20]. In a groundbreaking study that attracted international attention, between February and April 2021 Butantan vaccinated almost an entire Brazilian city, Serrana, using the vaccine it had helped test—cases fell by 80% and deaths by 95% [213, 214].

#### Diagnostics, therapies and devices

The overlap between rapid knowledge production and use continued with public/private partnerships in many countries, including in Brazil on the rapid development of diagnostic tests [172], and eventually in the NIH’s successful programme to develop diagnostics [215, 216]. These developments are further considered in Additional file 1, which also describes how various new COVID-19 therapies such as antivirals were developed and trialled, especially by US companies, sometimes building on previous NIAID-funded university research and often with some trial sites elsewhere, especially Brazil [162–164, 217, 218].

Now, however, we shall focus specifically on describing the unprecedented public/private collaboration in various activities in the development of new mAb therapies with funding from DARPA or BARDA and/or from the NIH’s ACTIV programme, and sometimes OWS involvement in promoting accelerated manufacture [96]. To assess treatments such as mAbs, two master protocols were established by the NIH: ACTIV-2 for outpatient trials and ACTIV-3 for inpatient trials [96].

AbCellera in Vancouver, Canada, supported by DARPA, had developed a mAb technology that meant it was ready to go in January 2020 [98], and working with NIAID’s Vaccine Research Center in the United States it discovered bamlanivimab, which was then developed with its US partner Eli Lilly as the first mAb therapy to reach human testing and to obtain FDA approval [219, 220]. While the development and trialling was increasingly taken over by Eli Lilly, the first tranche of science funding announced by the Canadian PM in March 2020 included support for AbCellera’s work [132]. Ely Lilly conducted their successful trial of bamlanivimab plus etesevimab for nonhospitalized patients entirely in the United States. It was published in *NEJM* [220]. As noted by Slaoui et al., there were also trials of bamlanivimab in ACTIV-2 and ACTIV-3 [96]. *NEJM* published a report of an OWS/NIH-funded ACTIV-3/TICO (Therapeutics for Inpatients with COVID-19) platform trial of the mAb in hospitalized patients that was halted when futility analysis showed lack of benefit [221].

A further study published in *JAMA* in June 2021 showed that bamlanivimab as a monotherapy reduced the incidence of infection among residents and staff of skilled nursing facilities. The study was sponsored by Eli Lilly and conducted in partnership with the NIAID and the COVID-19 Prevention Network [222]. An editorial about the paper highlighted both the success of the trial in mobilizing a federally funded clinical trials network to support the company trial, and also the challenges around using bamlanivimab as a monotherapy [223].

In July 2020, OWS announced that REGN-COV2 (later REGEN-COV), a neutralizing antibody cocktail of casirivimab plus imdevimab developed by Regeneron, was intended to be the first candidate therapeutic it would take through to commercial manufacturing, with a US$ 450 million investment [96]. In December 2020, a report of the earlier phase of the trial supported by BARDA showed success in reducing viral load in outpatients [224]. Its effectiveness in reducing the risk of COVID-19-related hospitalization, or death from any cause, was reported online in *NEJM* in September 2021 [225], and in August it had been shown to reduce the risk of COVID-19 in contacts of infected persons [226]. (However, and illustrating the challenges posed in a pandemic, in January 2022, the FDA withdrew its Emergency Use Authorization for both mAbs described above because they were not thought to be effective against the Omicron variant [227]. Then, on 11 February, the FDA gave Emergency Use Authorization to a new mAb, bebtelovimab, that was effective against Omicron and that again AbCellera had discovered and Eli Lilly developed [228].)

There were also efforts to accelerate the translation of basic research into medical devices. For example, in Brazil there was an attempt to use chitonase-based nanotechnology, manufactured in Brazil, in the filter element of the VESTA Face Respirator to reduce COVID-19 infection. It was developed through a partnership between academic researchers, a public laboratory and a private company, and made rapid progress described more fully in Additional file 1 [Private Communication from Mário Fabrício Fleury Rosa, 25 April 2022].

### Lesson 9—Informing policies and practice: the considerable divergence in the use of evidence to inform NPI policies, etc., and to promote equity in policies partly reflected established structures and cultures; collaborative living guidelines and good communications mattered

Across the seven countries there were examples of existing mechanisms and channels being leveraged quickly and effectively (and sometimes extended) to address the pandemic through use of evidence to inform policy-making and/or practice. This was particularly noticeable in Australia, Canada, Germany (especially at the start of the pandemic) and New Zealand. This mirrors the picture from Table 1, as these are the four of our countries with the most success in limiting COVID-19 deaths, although Germany’s early success was not fully maintained throughout 2020 and 2021. One feature of this evidence was the role of both locally produced research, often addressing local needs, and international evidence. In this component/lesson, the focus is primarily at the level of the relevant jurisdiction, as will also be the case for Lesson 10. Therefore, examples from each of these four countries, and where relevant from subnational jurisdictions, will be considered in turn, including in some cases the important development of living guidelines, which may also be of international relevance. It is also relevant to consider the role of communications before using the combined evidence to address the questions about the link between the role of the HRS in facilitating a willingness to use evidence, and the level of deaths.

#### Australia, including NSW

In Australia, as early as 31 January 2020, a leading APPRISE researcher reported they were developing research to share data with government departments to support decision-making during the COVID-19 emergency [229]. A report in May 2020 in *NEJM* from the NSW Ministry of Health identified how Australia had achieved an unusual national consensus on COVID-19 policies. Furthermore, expert committees had played a key role, meeting daily and advising bodies that created policies, recommended legislation and implemented laws related to COVID-19 [230]. From 1 February, the Australian government increasingly tightened its border control policies, and by mid-March it restricted entry for all foreigners.

Later reports continued to highlight the themes of policy-makers’ willingness to draw on the scientific evidence, and also adopt a more consensus approach than usual in the federal country. In December 2020, the Australian Academy of Health and Medical Sciences claimed that “by any global measure the response to date has been a spectacular success”, and it called for continued support for research to deliver “the knowledge and tools required to tackle the pandemic” [231]. Reports in December 2020 also suggested a generally high level of public trust in experts, and ministerial support for their input, with a leading role for previously marginalized Indigenous public health experts [232]. Researchers from the APPRISE project at the University of Queensland claimed in September 2021 that despite the global picture of Indigenous communities suffering disproportionate infections and deaths, “until very recently Aboriginal and Torres Strait Islander peoples had not recorded a single fatality” [117]. The Indigenous communities had effectively led and taken the initiative, and researchers were trying to help identify lessons [117].

However, addressing the Delta variant later in 2021, and then Omicron, proved more challenging than the first 18 months. There were fears that policy-makers were not sufficiently considering the concerns of community leaders and researchers about the dangers of ending COVID-19 restrictions before high levels of vaccination had been achieved in the Aboriginal communities [233]. (There was a dramatic surge of cases across many parts of the country into 2022, but while deaths increased from their very low level, they remained low by international standards.)

While rapid production of clinical guidelines became a feature of the pandemic in many countries, the approach in Australia was particularly significant. Pioneering research led in Australia that had resulted in the development of living systematic reviews [234, 235] proved very useful during the pandemic [38]. It was widely adopted, including by WHO [22].

But in addition, those Australian researchers who developed the concept had already worked with others to extend it. Their intention was to develop and deploy “a world-first, end-to-end, closed-loop evidence system for near real-time updating of systematic reviews and clinical practice guideline recommendations” [166]. Following the outbreak of the pandemic, this work accelerated. With funding from the federal and Victoria state health departments, they collaborated with a wide range of 31 (later 32) peak professional and policy stakeholders to create a national system of living clinical guidelines first published in March 2020, updated over 70 times by December 2021, and quite widely used by practitioners and policy-makers [24–26, 236]. Just as with primary and secondary research, a clear lesson seems to be that major, national, coordinated initiatives, with a high level of user involvement, seem more likely to produce high-quality outputs (in this case guidelines) that are valued and used, and that stand out from the explosion of small/poor-quality ones [42].

At the state level, the NSW government’s response to COVID-19 built on previous pandemic planning that had been informed by lessons from severe acute respiratory syndrome (SARS) and H1N1 influenza. As early as January 21, “NSW Health opened its Public Health Emergency Operations Centre… to coordinate case finding, contact tracing, outbreak control, communications, and other preventive actions” [230]. Over a 1-week period in March 2020, NSW Health also established the COVID-19 Critical Intelligence Unit to create a living evidence repository “to inform clinical policy and clinical practice…. [It] provides rapid and ‘good enough’ advice and transparency regarding the limitations of current, and often rapidly changing, evidence” [237].

The COVID-19 research programme from the NSW’s health ministry provides a strong example of effective co-production of evidence, consistent with NSW Health’s 2018 research strategy [83]. Authors from the health ministry reported that “the agile response of highly skilled and experienced researchers in close partnership with policy makers through the Emergency Response Priority Research workstream has ensured health decision makers have the best possible local evidence on which to base operational decisions” [27]. The evaluation of the NSW COVID-19 research programme emphasized how pre-existing relationships between academics and policy-makers were successfully leveraged through creation of a new structure for collaboration. It quoted the NSW Chief Health Officer as saying, “Some researchers have been able to be very nimble and can thrive in these environments….The researchers are given access to our data, it’s efficient, it’s evidence-based, it’s a win–win and a new way of working with researchers” [113]. Examples of research projects making a policy impact include the ones on sewage surveillance and also on COVID-19 transmission in schools described above [113, 203], and in more detail in Additional file 1.

#### Canada, including the provincial level

It is claimed that, compared to many countries, Canada holds evidence in high regard [107], and in making the announcement of the Can$ 1 billion investment in medical research, PM Trudeau said, “We are making sure that Canada remains at the forefront of scientific research to help us make smart and effective decisions on the path to recovery” [90]. One reason cited in 2020 for the much lower death rate in Canada was a greater willingness to listen to the evidence than existed in the United States under President Trump: “Though Canada’s response has not been entirely devoid of politics, Canadian officials have consistently deferred to public health experts and scientists to drive policy decisions” [238].

An analysis of why Canada’s COVID death rate was so much lower than that in the United States during the first 2 years of the pandemic also identified the role of scientists and public health experts in encouraging politicians to support and retain firmer NPIs than were generally applied in the United States [239]. But there were also other factors. These included the differences in the healthcare systems that existed at the start of the pandemic, with Canada having a universal publicly funded system that was lacking in the United States. There was also the vaccination rate, where Canada rapidly overtook the United States in the summer of 2021 [239]. By mid-August 2021, it was claimed that Canada had achieved “the highest vaccination rate—of single and double doses—anywhere in the world [through] a combination of savvy negotiations, financial resources and high trust in public health institutions” [240]. As Table 1 showed, at the end of 2021 Canada was still maintaining the highest vaccination rate across our countries.

While agreeing that Canada had controlled the virus more successfully than the United States, other analyses pointed to countries such as Australia and New Zealand as having a much lower death rate than Canada. One such article acknowledged the federal government’s crucial role in funding COVID-19 scientific research, but was critical of some other aspects of the Canadian response where the federal nature of the country did not seem to work so well, including failures on information sharing and “the inability to maintain an adequate public health surveillance system” to support local decision-making [241]. That article also highlighted the much higher proportion of deaths in the long-term care sector in Canada compared with other high-income countries. (However, it noted that the death rate in care homes in BC was lower than the average across Canada, something that Liu et al. thought was partly caused by the consistent communication about the pandemic from the Provincial Health Officer and elected leaders in BC—see below [242].)

Furthermore, an equity lens has not been consistently used in creating science-informed policies in Canada during the pandemic. There were calls as early as June 2020 for the initial modelling that “by necessity, assumed relative homogeneity in risks of infection and outcomes” to be replaced by an approach “[l]everaging data on heterogeneity to guide nuanced, population- and setting-specific strategies” [243].

Existing strong capacity in secondary research and guideline development in Canada was not only rapidly mobilized, but also extended. At the federal level, in April 2020, Innovation, Science and Economic Development Canada along with the support of the Chief Science Advisor Mona Nemer created CanCOVID as a Canada-wide network of health, science and policy researchers to facilitate COVID-19 research collaboration. It began conducting knowledge synthesis for policy-makers with its mission “to enable the agile, evidence-based decision-making needed to help steer Canada safely through the COVID-19 pandemic” [89]. Also, the known expertise in systematic reviewing and guideline development in institutions such as McMaster University, Hamilton, Ontario, were drawn on and enhanced through initiatives such as COVID-END (which received CIHR funding and leveraged the work of the SPOR Evidence Alliance) [168]. COVID-END was created as a time-limited network bringing together “more than 50 of the world’s leading evidence-synthesis, technology-assessment and guideline-development groups around the world”, to help the production and use of evidence syntheses and guideline development [168]*.*

Across Canadian provinces and territories there were studies conducted with the intention of informing policy and/or practice. Studies with an explicit aim of integrating a knowledge translation approach included one from St Michael's Hospital, Toronto, Ontario, for long-term care homes which sought to "integrate immunity study results to tailor delivery to improve COVID-19 preparedness and outbreak management" [244]. Some provinces also built on the CIHR-funded SPOR Evidence Alliance, with its aim of working towards a “collaborative research environment that is centred around patients and health system decision-makers” [167].

The evaluation of the BC HRS response to COVID highlighted the importance of the leadership provided by the Provincial Health Officer, Bonnie Henry, in directing the requirement for research coordination, and Michael Smith Health Research BC “through its long-standing and respected role as a broker between the research community and decision-makers” [93]. Health Research BC had launched a COVID-19 research competition in line with the strategic priorities identified in the province, as well as four research projects on urgent priorities requested by the Provincial Health Officer. The strong network of regional plus provincial health authorities, the BC Centre for Disease Control and the connection to the provincial government facilitated evidence-informed decision-making. The BC COVID-19 Strategic Research Advisory Committee worked to connect research results with the health system, with the coordination being “valued by senior decision-makers” in the health ministry and health service [93], although, as described in Lesson 10, some researchers reported facing continuing obstacles.

#### Germany, fluctuating success

The extent to which Germany, with a leading role for the Robert Koch Institute (RKI) (the federal government’s public health institute in Berlin) [245], has been able to draw on scientific evidence to achieve success in controlling the virus has been a matter of some debate. Lothar Wieler, President of the RKI, and colleagues claimed in March 2021 that “Germany demonstrates the difficulty of maintaining success throughout the COVID-19 pandemic” [246]. In terms of the initial response, Wieler et al. suggested: “The country’s strong enabling environment, including a good public health care system and expert scientific institutions contributed to the early success” [246]. Specifically, he noted that factors linked to the initial success included Germany’s prevention protocols, which facilitated a rapid response to the outbreak, the early development of testing capacity and high levels of testing, and an effective strategy for protecting older people.

In Germany, the government had a quite comprehensive National Pandemic Plan prior to the pandemic, although some public health facilities were understaffed and problems were encountered with shortages in personal protection equipment [246]. Germany was in the top 10% of nations on the GHS Index in October 2019, but eight other European countries were ranked higher [57]. All 16 of Germany’s states also had pandemic plans, but in the early weeks of the pandemic many politicians went beyond what the plans had defined in terms of consulting experts [247]. For one commentator writing in early April 2020, and comparing Germany’s much higher rate of testing and lower case fatality rate than in other countries such as the United Kingdom, “the country was meticulously prepared for a pandemic” [248]. A test protocol was rolled out in January 2020, and when required, the testing was conducted at well over 200 quality-controlled laboratories across the country [248, 249].

Various cross-country analyses reported favourably on the strengths of the pandemic response in Germany, and the willingness to engage with scientific evidence. The analysis in Germany for the study by Jasanoff et al. was concluded in late December 2020 and stated that “Germany’s public health response was characterized by a consistent pattern of delegation of policy questions to scientific authority (especially RKI) and a general appeal to rationality and solidarity” [250]. The analysis by Han et al. of the COVID-19 response in nine Asian and European countries identified Germany as one of four of the countries where “experts on infectious diseases within established public-health institutes are responsible for ensuring that scientific evidence drives policy making” [251]. Similarly, in an analysis of “co-producing the covid-19 response” in Germany and three Asian countries, Marten et al., suggested that various existing structures where researchers and policy-makers interacted in Germany were part of the pandemic response, including scientific advisory boards, research institutes and the RKI [51].

The early success also involved leadership by some of the state governments [249] and by Chancellor Merkel, who, according to an editorial in *Nature*, acted “on the basis of expert advice” [252]. Her own scientific background had, as national leader, established an insistence “that decision-making benefits from evidence”, which was also compatible with a wider political culture committed to rational responses driven by scientific data [250, 252].

The success in controlling the early wave was not fully continued into later waves of the pandemic [246, 251]. Chancellor Merkel “favoured an early return to tough restrictions—as advised by scientists—but the leaders of many of Germany’s powerful state governments refused” [252]. Wieler, too, suggested that the second surge saw “states deviating from federal recommendations”, and while the federal system allowed states to tailor their strategies, “it also limited widespread implementation of a standard testing strategy or national containment measure even in the face of rising case counts” [246]. Various potential weaknesses in the German response to the pandemic were also subsequently described. A detailed documentary analysis from a public health perspective of the role of expert committees suggested they were “not sufficiently representative and interdisciplinary to take different perspectives into account” [247]. Despite the initial success in introducing large-scale testing more rapidly than many other European countries, later, in the face of further surges and increasing disagreements between the federal and state governments, it was suggested that “the lack of reliable data” to fully inform policies, including data on issues such as variants, might have been one of the reasons behind the surges [253]. These issues are considered further in the Communications subsection below, and in more detail in Additional file 1.

During the pandemic, several organizations published recommendations, including RKI which updated the advice on its website over 20 times [254], or clinical guidelines. The Association of the Scientific Medical Societies in Germany (AWMF) produced living guidelines building on the living evidence syntheses from the COVID-19 Evidence Ecosystem project, described above [169]. A major goal of the project was to encourage translation by communicating the findings using the channels most relevant for specific target groups. The AWMF guidelines included national medical ones for the care of COVID-19 patients, but could also be at the population or public health level, such as the living guideline on measures for the prevention and control of COVID-19 transmission in schools [169, 171].

#### New Zealand, a unitary state

In New Zealand, the health research strategy’s priority of “translating research findings into policy and practice” [70] contributed to the context in which PM Ardern highlighted the importance of both science and her chief scientific adviser. She wrote that “science, scientists and science communicators have been at the forefront of the Government’s… fight to eliminate COVID-19…provide me with advice about the way forward, and to connect me and other Ministers with the range of scientific experts and communicators, both in New Zealand, and overseas” [28]. There seemed to be a combination of relying on scientific advice at the outset, and then increasingly also on (co-produced) findings from local research as well as continuing use of the international evidence.

A more detailed account of how the science was used appeared in *Nature Immunology* by Geoghean et al., who, as noted, produced some of the key evidence [30]. Under the title “New Zealand’s science-led response to the SARS-CoV-2 pandemic”, they explained: “The New Zealand government’s use of scientific expertise, spanning public health, infectious diseases, genomics modelling and immunology, has been one of the keys to the success of its SARS-CoV-2 elimination and control strategy” [30]. In *NEJM,* expert advisors also highlighted New Zealand’s “rapid, science-based risk assessment linked to early decisive government action”, and the PM’s effective communication [29]. In New Zealand, the model of researchers sometimes working closely with policy-makers was seen as positive [182]. Government officials or advisers were authors on several of the important papers, and in at least one case noted above, first author [201]. When Sarah Jefferies and colleagues were awarded their medal, Sunny Collings, HRC Chief Executive Officer, noted that the team both conducted research and provided real-time analysis and support to the Ministry of Health [182]. Key findings from studies described above [200–202] not only showed the impact and effectiveness of the initial NPI escalation and de-escalation decisions, but also continued to inform decisions [30].

#### Communications as a key NPI

Skills in effective scientific communications were also drawn on and enhanced, including in Australia, Canada, Germany and New Zealand [28, 29, 51, 113, 232, 255, 256]. Apart from Australia, these nations were included in analysis, conducted by UBC, of communications in nine countries selected partly because they “managed relatively effective responses” to COVID-19 [257]. The study, published in September 2020, found that communication was an effective NPI against COVID-19. Some themes were common across the jurisdictions: “Despite the differences, many of our case studies offered similar best practices: clear, evidence-based messaging; materials translated into multiple languages…. compassionate, empathetic acknowledgement of the difficulties of COVID-19 response” [257].

The study reported that at the federal level, Canadian public health communications were seen as “clear and understandable, emphasizing science and expertise; an innovation team embedded within the federal government incorporated insights from behavioural science to shape Covid-19 messaging” [258]*.* However, an analysis of the first full year of the pandemic took a rather different angle and claimed that “limited national and interregional coordination of public health communication was apparent throughout the pandemic, and the federal government fell short in leveraging its unique position to unite the public in supporting measures that help mitigate the pandemic” [241].

While presenting different perspectives about the national picture, both the above analyses praised the COVID-19 communications in BC led by Bonnie Henry. Furthermore, the cross-national COVID-19 communications report also looked in detail at two Canadian provinces—BC and Ontario—and reported favourably on the former. In BC, the approach seemed to mirror several of the important generic points listed above, with epidemiological information being accompanied by regular and extensive references to social or civic values as Henry successfully led the communications [259]. In addition to the comparison between the more effective communications and better outcomes in BC than in Ontario, the study also considered the situation in Sweden. It suggested the communication of the policies was more effective in Sweden than in Ontario, but the outcomes were worse because the policies being communicated in Sweden were less effective, and worse than those in Ontario or its own Nordic neighbours [259]. As one of several other communications initiatives in Canada to combat COVID-19 misinformation, a collective of independent scientists, healthcare experts and science communicators came together following a suggestion from Timothy Caulfield from the University of Alberta to create ScienceUpFirst [255].

The nine-nation communications report claimed that “[s]cientific expertise has clearly been the foundation for the German approach in dealing with Covid-19 but has also shaped communication strategies” [260]. It highlighted the important role of scientists such as Drosten and Wieler in communications and providing advice, with the key role played by Chancellor Merkel, who “joined her own scientific expertise with concern and empathy” [260]. However, later analysis suggested that gaps in the understanding of epidemiological figures by politicians and media professionals were particularly problematic given “the initially very academically oriented scientific policy advice” [253]. Problems for successful communications were caused by the insufficient digitization in the health system and other limitations in the data. These are discussed further in Additional file 1.

New Zealand was highlighted in the report as one of the cases where leaders had shared epidemiological modelling data with the population [257]. In New Zealand, communications were seen as a critical intervention, and the centrepiece of government response, namely a four-stage alert system for lockdown measures*,* was “introduced and explained clearly to citizens before restrictions were put into effect” [261]. The wider cross-national analysis by Han et al. included a discussion on communications and noted that while many other leaders struggled to secure public trust, PM Ardern “won national and international praise for communicating firmly yet empathetically” [251]. In New Zealand, Siouxsie Wiles, a biomedical researcher, science communicator and adviser to PM Ardern, won the 2021 New Zealander of the Year Award for her science communication during the pandemic [256]. Various countries, especially New Zealand, showed not only that research systems and policy systems could work closely together to produce effective evidence-informed policy to control the pandemic, but also how such policies could be effectively communicated to the public [28, 29, 256] by a leader who was trusted [52].

Journalists commenting on the communications report noted that, whereas some governments have been praised for the science-driven way that they have communicated about the pandemic, others not included in the nine, “most notably the U.S. and the U.K., have been hit with criticism for public health messages that are confusing or not based in science” [262].

Taking a historical perspective, the above experiences reinforce lessons from previous research conducted at a time of preparation for a potential flu pandemic that had also highlighted the importance of developing ways to communicate effectively with the public during health crises, including the key role played by trust [263].

#### Links between willingness to use evidence, effective leadership and success in controlling the virus

Overall, efforts such as those above to promote and facilitate evidence use in policies, practice and communications were variously supported by a range of factors (further identified and explored in Additional file 1) that can, at least in part, be linked to the HRS. These include an explicit mandate for evidence use from the strategy for health research, as in NSW [83] and New Zealand [70]; established cultures favouring the use of evidence, as in Australia/NSW [83], Canada, including BC [93, 107, 167], Germany [250, 252] and New Zealand [28, 52]; formal structures, such as the National Pandemic Plan and state plans in Germany [246, 247], the APPRISE pre-planned research platform in Australia, and state ones as in NSW [85, 230]; specific programmes of co-produced research aimed at informing policy, as in BC [93] and NSW [113]; the readiness of teams who were already pioneering new approaches to promoting evidence use to take them further, as in Australia [24, 25, 166] and Canada [168]; and the use of evidence in communications, as in Canada, including BC, Germany and New Zealand [257].

In drawing lessons, it is important to note that even if countries such as the United Kingdom, United States and Brazil produced many key research findings and translated them into life-saving products, their countries suffered comparatively high death rates at least in part because of the failings of their respective leaders in relation to component 9, the use of evidence to inform policies. It is also the case that countries that controlled the virus best, had the fewest patients who could potentially be included in trials.

Inevitably, however, just as there were some examples described in Additional file 1 when leaders in Australia, Canada, Germany and New Zealand faced challenges in using the evidence to inform policies, there were also examples of policies, and especially practice, being informed by evidence in Brazil, the United Kingdom and United States. It is useful to consider a few instances here. They include, in Brazil, the United Kingdom and the United States, the vaccine rollout policies which, as noted in component 4, have already been assessed as saving many lives [5–8, 125]. For example, in the United Kingdom the vaccine rollout organized by the Vaccine Task Force led by Kate Bingham, which had responsibility for the whole vaccine programme in the United Kingdom, was seen as being particularly rapid and effective [103, 210], although as Table 1 shows, the vaccination rate in the United Kingdom, along with that in the United States, is now falling behind other nations. In both Brazil and the United States, some local leaders were more willing than others to draw on the evidence to inform policies [206, 264, 265]. For example, a study in Brazil showed there were on average fewer deaths and hospitalizations in municipalities with a female mayor, and that “[l]ower overconfidence and more weight to scientific advice among female leaders may also explain why they enforce more NPIs” [206].

In all three countries, clinical guidelines were produced to inform healthcare practice in the pandemic [266–269]. For example, in the United Kingdom, the National Institute for Health and Care Excellence (NICE) produced COVID-19 guidelines much more rapidly than usual (19 by the end of 2021) [267, 268], as did the NIH in the United States, where, for example, an NIH guideline related to mAbs for nonhospitalized patients was updated regularly [269]. Furthermore, in all three countries (as elsewhere), there has been widespread praise for the millions of healthcare staff doing their best, often in the most difficult of circumstances, to provide evidence-based care.

Nevertheless, despite these and other examples, and notwithstanding their successes in the production of vital scientific breakthroughs, the worse-performing countries from Table 1, namely Brazil, the United States and, to some extent, the United Kingdom, were those where, as Additional file 1 details, there were well-documented failures by political leaders to use the scientific evidence consistently to inform policy-making. This is described, for example, in editorials in *Nature* and in *The Lancet* criticizing President Bolsonaro for being dismissive of the science [270, 271]. Similarly, a *NEJM* editorial in late 2020 criticized how, in the United States, experts were ignored or denigrated: “Our current leaders have undercut trust in science and in government, causing damage that will certainly outlast them. Instead of relying on expertise, the administration has turned to uninformed ‘opinion leaders’ and charlatans who obscure the truth and facilitate the promulgation of outright lies” [272].

Marcia Castro, a Brazilian at Harvard’s School of Public Health and second author on the Victora et al. paper [8], described how Brazil had an immunization programme that had previously worked well and suggested that “Brazil could have given a lesson to the world on how it handled its response to HIV/AIDS and Zika epidemics” [265]. However, President Bolsonaro’s COVID-19 response was a failure of leadership because he and “Donald Trump were similar in their denial of how serious the virus was and their denial of science” [265]. It has also been claimed that in both countries the approach of the national leader could make it challenging for local policy-makers to draw on the scientific evidence [43]. This might allow comparison with subnational policy-makers in federal countries where the national leader was more supportive of the science. The adoption and promotion of chloroquine and HCQ by the Brazilian Government, including through its Ministry of Health, provides a major example of the political pressure to act in the absence of the usually required research evidence (or subsequently to ignore the evidence when it was available). President Bolsonaro replaced a health minister who seemed to oppose recommending these drugs with an army general, and immediately the Ministry of Health implemented a protocol for the use of the drugs ([273—translated], and see Additional file 1 for further details).

The position of the United Kingdom in this analysis is complicated, because while the HRS that had been built on a comprehensive strategy worked extremely well in terms of knowledge production and product development, the use of evidence at the national level was much more challenging. Specific aspects of this are discussed further in the next lesson, and also in Additional file 1.

There seems to be a clear lesson that having HRSs in which there are skills, mechanisms, structures and cultures to undertake and promote the use of evidence creates situations in which these attributes can be mobilized by political leaders who prioritize the saving of lives. Reflecting on the United States’ top score on the 2019 GHS Index, it was noted in April 2020 that the index “did not anticipate the poor response to the pandemic by high-scoring countries such as the US where major gaps in federal leadership resulted in a failure to mobilize the country’s substantial capacity” [103]. According to one researcher looking at the high scores but poor performance by the United Kingdom and United States, “we had everything—except leadership” [274]. One of the GHS Index team was quoted as saying, “Even though the US and UK had the best environments in terms of plans in place and thinking about what they would need in terms of capacity…when it came to the moment that everyone had been preparing for, the decision-making really hampered the actual ability of the country to respond” [274].

Leadership and evidence-based policies were required from the start. However, Additional file 1 describes evidence from papers in *The Lancet,* and WHO reports, that should have led to preparation and action at least in February 2020, but these opportunities were missed in the United Kingdom [210]. Farrar reported the claims that PM Johnson had not been paying attention to the pandemic in the vital early weeks [103] - this period when action was required was well before the PM first tested positive for COVID-19 on 27 March 2020, and well before the eventual announcement on 23 March 2020 by the government of the United Kingdom of the delayed first lockdown. A more detailed examination of the US performance on the GHS Index in October 2019 reveals that some of the seeds of the problem were already there, and had probably been exacerbated by President Trump. For example, in addition to the US ranking of 175th out of 195 countries on the specific item for healthcare access, it had the lowest possible score of zero—it failed to reach the 25% threshold—for public confidence in the government, which is very serious because lack of public trust is likely to undermine disease control and public health messages [61, 275].

Finally, it is noticeable that countries such as the United Kingdom and United States with higher death rates also tended to perform badly in terms of equity [121, 181, 210, 251]. Thus, having an effective HRS does not guarantee policy success (or that an equity lens will be used in science-informed policy), and this will be discussed later, but the key question for now is what lessons can be drawn about the best ways to increase the chances that the various components of an effective HRS are present.

### Lesson 10—Research strategies: pre-existing comprehensive health research strategies and vision enhanced the effectiveness of specific steps and opportunities for producing research to improve policies, practice and health, but did not ensure informed action

The thinking of perhaps more than one system is well described in a report from the Association of Australian Medical Research Institutes (AAMRI). In November 2021 it claimed, “It is no accident that Australia has been able to mount such a strong response during this pandemic; it has been made possible by decades of investment in building up our health and medical research capacity” [276]. However, the report also noted some concerns about Australia’s medical research: a lack of national coordination of priorities or sufficient identification of needs; problems with job security and gender inequity; and that “[c]ollaboration and cooperation between research and healthcare delivery remains fragmented” [276]. While these were long-term issues that had been exacerbated by the pandemic, the report called for all stakeholders in the medical research sector to come together and develop “a National Health and Medical Research Strategy” [276].

As noted, the WHO evidence synthesis of policies and tools to strengthen HRSs [66, 67] highlighted the comprehensive health research strategies both in New Zealand [70] and behind the creation of the NIHR in England [69, 71]. Consideration of the effectiveness of these overall strategies inevitably involves collating some material discussed in the lessons related to specific components. Below, we also briefly consider two of the subnational systems where comprehensive systematic thinking has been of value: BC and NSW.

#### New Zealand

In New Zealand, the coherent HRS had been established in 2017, with responsibilities shared between the health and business ministries and the HRC. In the wider health and political system, however, at the outset of the pandemic there was a major challenge in terms of “a general lack of planning, public health investment and readiness” [277]. As shown on Table 1, the GHS Index published in Oct 2019 showed that New Zealand lacked the capacity of other high-income countries [57]. Siouxsie Wiles thought the lack of readiness illustrated by the rank on the GHS survey encouraged a science-based decision-making process to lockdown [278]. She explained that “countries that thought they were prepared have done very badly…We knew our testing and hospital capacity were really bad… we couldn’t just rely on testing and contact tracing” [278].

In the context of a wider lack of readiness, the New Zealand HRS was ready to respond in a broadly successful way despite its inevitably small size. There were some contributions to the breakthroughs made by the global REMAP-CAP study, but they were very limited by the low volume of COVID cases, in addition to the relative smallness of the research system [110, 192, 279]. There appeared to have been recognition that it was better for a small system to aim mostly to contribute its clinical trial activity to larger international trials, either global, as with REMAP-CAP, or Australasian, as with ASCOT [110, 280]. Additionally, however, much of the research conducted was relevant for specific COVID-19 issues facing New Zealand [110, 200–202, 281]. The research findings were then often effectively used and communicated, sometimes with researchers simultaneously working alongside policy-makers [28–30, 182]. In 2020, policy experts working in New Zealand analysed the importance for any system of mobilizing expertise to deliberate on public policies; the accounts above describe just how successfully the New Zealand HRS had been mobilized to inform the widely praised policies [52]. Similarly, several expert international teams praised the successful communications of the evidence-informed policies [251, 257].

Many of the experts who had hitherto supported PM Ardern’s approach were surprised at the apparent comparatively limited consultation when she announced a change in strategy in October 2021 [282]. This change was also strongly criticized by some leaders of the Māori and Pacific communities [283], who were disproportionately likely to be hospitalized and had lower vaccination rates, and whose potential greater vulnerability to the virus had been thought to have been a motive for the original elimination strategy [284].

However, the numbers in Table 1 by the end of 2021 suggest that there had not been a surge in deaths, and the death rate was still considerably lower than that in any of our other countries (and continued to be very low by international standards in 2022 despite a jump in cases and deaths as Omicron spread [46]). So, overall, it seems reasonable to conclude that the various features of the research response, as well as the commissioning of research looking to promote equity in the response to the pandemic [111, 112], reflected the comprehensiveness and vision of the 2017 New Zealand health research strategy [70]. The contribution was perhaps best summed up by Sunny Collings, who said in November 2021, “New Zealand’s relatively small health research workforce has worked tirelessly together with other health professionals to provide fast and accurate information to try to minimise the impact of COVID-19 on our communities” [182].

#### The United Kingdom

As described in the lessons above, in the United Kingdom the organization, structure, and accompanying capacity of the HRS contributed to successes in relation to most of the HRS components. It was the existing networks, governance arrangements and capacity within the United Kingdom’s HRS (much of it in line with the comprehensive strategy adopted in 2006) that was very effectively mobilized in response to the pandemic, including in various COVID-related trials in the United Kingdom [1, 3, 9, 10, 18, 39, 104]. The affiliations of Peter Horby and Martin Landray, who created RECOVERY, included Oxford NIHR Biomedical Research Centre and MRC-funded centres [9, 103, 127], and the NIHR structures and capacity were already well embedded and coordinated in the integrated healthcare system of the NHS [1, 3, 10, 39, 102, 103]. As noted, flexible adaptations could be made where necessary, for example to the prioritization, data systems and organization of the research capacity, to further facilitate the rapid establishment and implementation of the study throughout the 176 NHS hospital trusts in the CRN [1, 10, 102, 123, 127].

Being able to rapidly mobilize the considerable health research capacity that existed throughout the healthcare system meant that RECOVERY soon established itself as the world’s largest COVID-related therapy trial [1, 3, 10, 39]. As result, a series of key findings about therapies that worked, including dexamethasone, and those that did not, including HCQ, emerged rapidly from RECOVERY, with important contributions also from REMAP-CAP [1, 3, 9, 10, 39, 102, 104]. More generally, when Atkinson et al. complemented their analysis of the NIHR’s formation [74] with an assessment of how the HRS had responded to the pandemic, they reported on the “research readiness” of UK science which meant it was able to “respond rapidly with, for example, clinical trials” [285]. By contrast, concerns were expressed in various other countries about their more constrained clinical trial progress and/or the amount of waste that arose from the extensive duplication from many small studies that lacked the power to make a valid contribution [37, 39–41, 99, 107].

Suggestions that systems should look at what was being achieved in the United Kingdom came from an international perspective [36], as well as countries such as Australia [101], the United States [40, 41, 100, 179], across Europe [39] and Canada [99]. In the latter, there was a particularly detailed analysis conducted in 2020 by Lamontagne et al. of how the comparatively limited contributions to globally major breakthroughs during that year, despite considerable funding, could be linked to gaps in the system, with no equivalent coordinated clinical research networks operating across the type of integrated national healthcare system that exists in the United Kingdom [99]. They suggested that the limited contributions by Canada “have highlighted a broken system”, and they pointed to the NIHR being embedded in the healthcare system which “simultaneously solved problems related to infrastructure development, health system engagement and fragmentation in the UK context” [99].

(Their analysis is described more fully in Additional file 1, where there is also an account of developments in Canadian research throughout the pandemic, including some progress, for example, as they noted, in Lamontagne's own province of Quebec [99, 286]).

Despite the successes in the United Kingdom with research production, problems rapidly emerged with component 9 on using evidence to inform policy and communications, and this was an area where an analysis of the NIHR published in 2010 had suggested more progress was being made by HRSs in Canada than in the United Kingdom’s NIHR [73]. Additional file 1 describes the considerable debate about the extent to which the HRS, or at least leading researchers who advised the government in the United Kingdom as members of the Scientific Advisory Group for Emergencies (SAGE), should share some responsibility for the government’s failure to introduce NPIs sufficiently rapidly in spring 2020.

Responsibility for policies in the United Kingdom that ran the risk of high death rates in the subsequent waves seemed clearer. Based on his first-hand experience of events in autumn 2020, Jeremy Farrar, Director of the Wellcome Trust, wrote, “I respect the mantra that scientists advise and ministers must decide, but ministers were clearly overriding SAGE advice, often while claiming to follow it…I began to question the point of giving advice to a body that chose not to use it” [103]. Additional file 1 provides a more  detailed analysis of how Farrar came to this conclusion about the UK government overriding the science with the "delays that preceded the second lockdown, despite the wealth of data pointing to imminent disaster", and his key conclusion was that “[m]any of the UK’s Covid-19 deaths happened in January, February and March of 2021; they were avoidable. The political decisions made, or not made, in the second half of 2020 were unforgivable” [103].

A key contrast between New Zealand and the United Kingdom was made by policy analysts familiar with both systems. They described the importance of effectively mobilizing expertise to deliberate on public policies. They said that lessons could be learned by contrasting PM Ardern’s successful science-based approach with that in the United States and the United Kingdom. They also commented on a broader move by recent UK governments away from a previous pattern of deliberation with expertise: “the British government’s stumbling (at least from a New Zealand perspective) response to COVID-19, hard on the heels of the policy-making ‘omnishambles’ of the UK’s departure from the EU, is nothing unusual” [52].

#### Subnational-level strategies and progress: NSW in Australia, BC in Canada

NSW Health’s comprehensive 2018 strategy for population health research was noted earlier [83]. This was mirrored in the comprehensive COVID-19 research programme that included items such as process work streams to accelerate approvals, and an impact evaluation plan as well as research projects targeted on priorities including the needs of Aboriginal communities. Many of the projects built on existing partnerships, and some of them adopted an advanced form of co-production that built on the strategy but went even further in terms of joint working and collaboration between researchers and policy-makers [113]. The role of long-established partnerships such as that with the Sax Institute illustrate how the strategy’s commitment to the use of evidence in policy-making was amplified during the pandemic. With funding from the NSW COVID-19 research programme, the institute pivoted from its 5-yearly *45 and Up* health survey to electronic surveys “co-produced” with the Ministry of Health and asking key policy-relevant questions, including as the pandemic continued, about “missed healthcare and mental health during the pandemic” [113, 287].

NSW Health opened its Public Emergency Operations Centre as early as 21 January 2020 based on previous pandemic planning [230], and the creation of the COVID-19 Critical Intelligence Unit in March 2020 reflected the strategy’s emphasis on the importance of using scientific evidence [83, 237]. The evaluation of the COVID-19 research programme in May 2021 presented a positive picture of progress to date, with the co-produced projects, in particular, being viewed as being extremely helpful by the leaders of NSW Health. Taking a strategic approach, the evaluation framed the analysis by suggesting that in response to a public health emergency such as the COVID-19 pandemic, “An established and agile research infrastructure is a powerful tool in enabling rapid research production and knowledge dissemination” [113]. It also described the plans for the final evaluation and impact assessment, and that a pathway had already been created “for efficiencies in future emergencies and business-as-usual procedures” [113]. NSW had a lower death rate in 2020 and 2021 than even the low figures for Australia as a whole [288].

The analysis in relation to BC is inevitably more tentative because the comprehensive health research strategy proposed in 2014 was not fully implemented [79]. Nevertheless, various gaps and opportunities described in the strategy were subsequently addressed. Furthermore, at the outbreak of the pandemic, new coordination structures such as the Clinical Research Coordination Initiative were created, and the BC COVID-19 Strategic Research Advisory Committee was established to connect the needs of the leaders of the provincial government and health system with the health research community [93].

The coordination was valued by senior decision-makers in the health ministry and health service, who reported that they were receiving information to address their questions, even though some BC researchers themselves reported some continuing limitations in structures to support (rapid) research within the health systems [93]. As noted, the health system’s communications to the public in BC, led by the Provincial Health Officer Bonnie Henry, were viewed very highly in the cross-country comparison [257, 259].

### Lesson 11—Negative impacts: the pandemic damaged aspects of HRSs—reduced resources/opportunities especially for non-COVID-19, early-career, female and minority researchers; problems completing projects in lockdowns; reductions in public involvement

This opinion paper does not focus on the undoubted negative impacts the pandemic has had on some aspects of HRSs, and while some examples are mentioned below, this would be a topic for further study. Nevertheless, the Australian report above also illustrates how some of the issues related to reforms to improve the pandemic response can also become entwined with more long-standing issues such as career progression and gender equality in the research workforce [276].

Notwithstanding the successes resulting from prioritization, important issues about balance had to be faced, because the strong concentration on a few priority COVID studies inevitably created difficulties for those in other fields of research. These challenges were particularly acute in the United Kingdom for many researchers who, in practice, had to wait until they were allowed to start or continue research on other topics within the NIHR/NHS [10, 102].

In Canada, challenges faced by the HRS during the pandemic included reduced funding in some areas (e.g. in 2020 CIHR delayed one of their project grant competitions, thereby impacting hundreds of researchers [289]). Furthermore, in Canada and the United States, many researchers, especially women, noted that the pandemic had had a negative impact on their career trajectory, and women from the Black, Indigenous and people of color (BIPOC) communities were particularly disadvantaged because of the pre-existing structural inequities [107, 290, 291]. These issues were particularly challenging for female medical academics with the need to do more clinical work, but could be addressed in ways such as those being advanced by provincial funder Health Research BC and that CIHR has already advanced in some competitions [290, 292].

A survey/workshop conducted in May 2020 of German health researchers who had been working on non-COVID topics reported that 93% believed their projects were affected by the pandemic [293]. Eighty percent reported that they could not collect data as planned, with problems also caused by staff being unavailable because of care commitments, illness or quarantine. The majority had mitigation strategies in place, including adjustments of data collection through the use of digital tools or changing the research design [293]. Many of these approaches could be taken forward after the pandemic.

There were also concerns that the wider system of PPI in research was often reduced during the pandemic and risked becoming tokenistic [93, 107]. Challenges included the need to prioritize rapidly, especially at the beginning of the pandemic when all timelines were greatly compressed [102], and more generally because of the shift online during the pandemic—though some PPI representatives were positive about what emerged as the inclusive benefits of virtual PPI [102]. As with the development of new research techniques, the pandemic encouraged the development of new approaches, for example, in England, a centralized matching system to rapidly identify potential PPI representatives for COVID-19 proposals [294]. The final aspect of this lesson reflects some of the previous analysis in recognizing that addressing the issues will not be easy. While HRSs have been widely praised for their rapid contributions to controlling the pandemic, the damage inflicted on economies by the pandemic will create challenges in securing the funding to address the problems, on top of meeting all the future research needs.

### Drawing on the lessons and our questions in devising recommendations to strengthen HRSs

The lessons above enable us to address the questions posed at the beginning of our analysis and to propose related recommendations. Collectively, the lessons demonstrate that life-saving scientific pandemic achievements that could become globally available did depend to a considerable extent on national HRSs (and, therefore, that scientific achievements generally do depend on HRSs). This is particularly clear in relation to question A(i), where systems with the most developed structures and capacity, especially where as with the United Kingdom there was an overall strategy, were able to make rapid progress in knowledge production that could, where relevant, lead to product development. Even here, it was often necessary to go further than the pre-pandemic situation, especially with coordination, prioritization and financing, but the existing structures and capacities provided strong foundations. However, further factors came into play because the countries that controlled the pandemic least well, such as Brazil, the United States and the United Kingdom, were inevitably the ones that had the most COVID-19 cases to potentially enter into trials for repurposed drugs and thus make the most progress in such research, and vice-versa for Australia and New Zealand.

The picture is more complex in relation to question A(ii). Across our seven countries it did seem clear that where there was a willingness to use evidence in the most appropriate way given the situation of the country, then, as with Australia and New Zealand in particular, it led to considerably fewer deaths than occurred in countries such as Brazil and the United States where the presidents frequently dismissed the scientific evidence. Of course other factors such as location, healthcare access, racial and other inequalities, financial resources and the level of trust in the political leadership also played an important part, but a willingness to use evidence mattered, and also meant that the resources used locally to produce the relevant evidence were not being wasted. Many features of strong HRSs helped facilitate the use of evidence to inform life-saving practice and policies. By themselves, however, they could not guarantee political leaders would be willing to use the evidence in ways that would give priority to life-saving policies. (In relation to healthcare practice, there was often scope for evidence use to be facilitated by the HRS irrespective of the approach of the political leaders.)

Further complications arose when federal leaders were keen to use evidence but faced challenges in getting all subnational leaders to do the same. Such challenges sometimes arose in Canada and Germany, where at times other challenges also included the availability of sufficient data to inform policies and devising consistently appropriate communications. In the United Kingdom, the extent to which the national leader was willing to use evidence was contested, which made the analysis even more complicated.

Finally, in relation to question B, it is clear that the avoidance of research waste in the production of life-saving research depends substantially on the existence of a comprehensive and coherent HRS, as in the United Kingdom.

While it could be seen as a limitation of our study that we included just one non-high-income country, Brazil, we believe the example could be instructive for other middle-income countries. Having built its health research capacity over many years, Brazil was in a position to rapidly conduct important COVID-19-related research as described throughout this article. Nevertheless, we recognize that resource constraints and inequitable global distribution of vaccines and new drugs posed additional challenges for low-income countries during the pandemic.

Although the focus of our opinion paper is lessons, based on the analysis it is possible to go beyond confirming that a jurisdiction’s HRS can indeed contribute to the health and well-being of its citizens, to propose how this might happen. Therefore, building on the 2020 WHO evidence synthesis on strengthening HRSs [66, 67], we have developed a set of recommendations (see Table 4) that we hope could be useful for jurisdictional stakeholders interested in strengthening their HRS. The recommendations acknowledge that HRSs are not immune from social and political factors outside their boundaries that can have deleterious effects—but that there may be ways to mitigate or at least account for these effects.

**Table 4:** Recommendations related to each HRS function/component

HRS functions/components	Recommendations related to HRS components, comprehensive strategies and negative impacts
*Governance*	*Governance*
1 Coordination	1 Enhance coordination of the governance function—ideally as part of an overall health research strategy, at minimum as pandemic preparation
2 Priority-setting	2 Develop transparent mechanisms, including using an equity lens, for wide/public engagement in priority-setting, rapid centralized (trial) prioritization in a crisis and encourage adherence to and monitoring of priorities, which may require adjustment over time
3 Ethical approval	3 Identify ways, including increased resources, to sustain pandemic progress and accelerate ethics and other approvals and enhance data access and sharing
4 Evaluation	4 Incorporate into evaluation approaches routine assessment of research impact on health policies, practice, equity, and health and economic well-being; recognize the new opportunities for more rapid assessments
*Financing*	*Financing*
5 Securing finance	5 Encourage continuation of enhanced research funding by documenting impacts from COVID-19 research and discourage cuts in non-COVID-19 research by analysing the damage already caused; identify ways to tackle waste
*Capacity*	*Capacity*
6 Capacity-building	6 Train and sustain as wide a range of research capacity as can be afforded and include pandemic planning; integrate clinical research capacity into healthcare systems; enhance/build capacity to use and communicate research
*Production and use*	*Production and use of research knowledge*
7 Knowledge production	7 Continue the accelerated methods of research production—where possible using the new vaccines platforms, more adaptive platform trials, transdisciplinary research and, if relevant, co-production; continue the trends towards rapid open access publication
8 Promote use in new products	8 Continue accelerated translation of research into new products by, where appropriate, encouraging continuation of unprecedented levels of public/private collaboration and public funding of research and regulatory bodies
9 Translate to inform policies, practice and opinion	9 Promote structures, cultures and networks that encourage the use of evidence relevant for the needs of policy-making and practice using an equity framework; recognize the importance, and fund, collaborative living guidelines; invest in strategic, evidence-based communication that accounts for current political realities, the influence of key stakeholder groups and individuals, and variations in audience understanding and preference—and is therefore trustworthy
Comprehensive strategies for health research	10 Develop and implement comprehensive health research strategies enhancing the effectiveness of specific steps and maximizing opportunities for producing research to improve health policies, practice, equity, and health and economic well-being
Negative impacts on HRSs	11 Use the HRS strategy to help address problems created, or exacerbated, by the pandemic; build on steps taken to address funding/early-career/minority/gender issues; recognize and promote improved (digital) techniques developed to facilitate research and engagement during the pandemic

Source: Extensively adapted from Pang et al. (2003) [45] and Hanney et al. (2020) [66, 67]

In the area of HRS governance, the need to improve coordination is clear, as are the importance of priority-setting mechanisms—including those that enable broad stakeholder engagement—and rapid approvals of ethics, data access and sharing, and study protocols, in as coordinated a way as possible. Another important area of HRS governance is evaluation; given the unprecedented resources that were allocated for COVID-19 research and the importance of understanding where and how impact was maximized, it is recommended that funders step up their efforts to incorporate routine impact assessments at the project and system level. These assessments will assist in another HRS component: appropriate and adequate financing. Funders should work to determine the benefits of the increased funding for COVID-19 research and, on the other side, the damage done by the loss of funding for non-COVID-19 research; they should also take advantage of an opportunity to address many of the system conditions that result in research waste.

Important contributions came from rapid mobilization of existing primary and secondary research capacity, enhanced interdisciplinary cooperation and clinical research integrated in healthcare systems. Therefore, we recommend training and sustaining as wide a range of research capacity as possible, and improving the integration of research directly into healthcare settings. This integration must be multifaceted, involving infrastructure, culture change, education and skill-building.

In the area of knowledge production and product development, accelerated methods of developing vaccines and other evidence greatly enhanced the pandemic research response. We therefore recommend the continuation of accelerated methods of research production and product development, including the trend towards open access publishing that was seen during the pandemic. Journals will ideally continue to be as flexible as possible to ensure rapid peer review and open access publication of key papers, along with making the study data public.

In the HRS component of using evidence, it is worth examining the extent to which policy-making was informed by evidence, and served to limit deaths during the pandemic. An understanding of this crucial element of the response may enable researchers and policy-makers to work more closely together in the future—facilitated by appropriate mechanisms and structures—to more closely link evidence and policy. Focused attention to evidence-informed communication—that accounts for current political realities, influence of key stakeholder groups and individuals, and variations in audience understanding and preferences—is clearly warranted, and crucially should help to build trust.

Some of the lessons from the pandemic, including the point immediately above, reinforce lessons from analysis of earlier pandemic threats [263]. However, additionally, there is no doubt that countries are already starting to learn from the lessons from COVID-19 about responding to future pandemics and also for addressing chronic illnesses going forward. For example, NSW Health’s COVID-19 research programme addressed blockages in the administrative processes of the HRS and created a pathway “for efficiencies in future emergencies and business-as-usual procedures” [113]. In Germany, one of the goals of the Network of University Medicine was the “generation of findings also for better preparation for future epidemiological events” [94]. Teams that developed the new vaccine platforms are already exploring ways of using the technology to develop vaccines for other illnesses [18, 114], and the teams that successfully conducted adaptive platform trials, especially the RECOVERY team, have developed plans to build on the pandemic advances by making such trials part of standard good clinical care in order to maximize research advances to address major diseases [3].

We are hopeful that, coming out of the pandemic, in addition to attending to specific components of HRSs such as coordination, approvals, production and translation of knowledge, jurisdictional stakeholders explore the important interactions among these components, as well as the factors external to the system that are likely to affect its success, in overall HRS strategies.

## Conclusions

Numerous research achievements have saved the lives of millions of people worldwide during the COVID-19 pandemic. Alongside achievements, there have been many research-related challenges, including pressure to rapidly develop and deploy evidence, and duplication and waste of resources. We explored to what extent these achievements, and the ability to address the challenges, appear to have built on the existing readiness of HRSs. We used a WHO framework that identifies the four main functions of HRSs (governance, financing, capacity-building, producing and using research), and which should prove instructive for jurisdictions interested in maximizing the impacts of health research. We developed lessons based on analysis of key articles with relevance to Australia, Brazil, Canada, Germany, New Zealand, the United Kingdom and/or United States. We explored the efficient production and synthesis of COVID-19 evidence, the effective promotion and facilitation of local and global evidence use in policy and practice, and the challenges in doing all this.

The lessons demonstrate the importance of governance in the focusing of resources, including coordination, priority-setting and the acceleration of much-needed research approvals. It is clear that additional funding during the pandemic was key to life-saving research, but that some resources were wasted in duplication and on small studies. The research response to the pandemic benefited from the existing capacity of HRSs—including existing primary and secondary research capacity, and especially that embedded into research-ready health systems. Nevertheless, the acceleration of research production did not automatically translate into efficient and effective policies and practices. In particular, the use of evidence did not consistently promote equity-based policy. Further, while pre-existing health research strategies seem to have enhanced some jurisdictions’ response, they did not ensure informed action. Finally, the negative impacts of the pandemic on the HRS and researchers—due to reduced resources for non COVID-19 research and the effects of lockdowns on research—will have long-term ramifications.

In sum, our analysis demonstrates the benefits of greater coordination and integration within HRSs, and between health research and healthcare systems—both of which can be achieved via a comprehensive health research strategy. Given our finding that such strategies do not necessarily guarantee the use of evidence to inform policy-making, we recommend that development and implementation of jurisdictional health research strategies involve broad stakeholder engagement to maximize input and collaboration.

Finally, although further analysis of the downsides of the pandemic on health research is warranted, there is no doubt that HRSs will experience challenges because of non-COVID-19 research as well as research careers—especially those of early-career, minority and female researchers—having been put on hold. HRS recovery is another compelling reason for jurisdictions to develop comprehensive health research strategies, involving as many stakeholder groups as possible (including patients) in identifying problems and developing long-term solutions.

## Supplementary Information

**Additional file 1.** Examples of responses to the pandemic in seven health research systems

## Data Availability

Not applicable for this opinion paper, but the Additional files contain a collation and account of many of the sources used.

## Abbreviations

AAMRI: Association of Australian Medical Research Institutes
ACTIV: Accelerating COVID-19 Therapeutic Interventions and Vaccines
APPRISE: Australian Partnership for Preparedness Research on Infectious Disease Emergencies
ASCOT: AustralaSian COVID-19 Trial
ATTACC: Antithrombotic Therapy to Ameliorate Complications of COVID-19
AWMF: Association of the Scientific Medical Societies in Germany
BARDA: Biomedical Advanced Research and Development Authority (US)
BC: British Columbia
CIHR: Canadian Institutes of Health Research
CRN: Clinical Research Network (England)
DARPA: Defense Advanced Research Projects Agency (US)
EU: European Union
FDA: Food and Drug Administration (US)
Fiocruz: Oswaldo Cruz Foundation (Brazil)
GHS: Global Health Security
HCQ: Hydroxychloroquine
HRC: Health Research Council (New Zealand)
HRS: Health research system
JAMA: *Journal of the American Medical Association*
mAb: Monoclonal antibody
MRC: Medical Research Council (UK)
mRNA: Messenger ribonucleic acid
NEJM: *New England Journal of Medicine*
NHMRC: National Health and Medical Research Council (Australia)
NHS: National Health Service (UK)
NIAID: National Institute of Allergy and Infectious Diseases (US)
NIH: National Institutes of Health (US)
NIHR: National Institute for Health Research (England)
NPI: Non-pharmaceutical interventions
NSW: New South Wales
OWS: Operation Warp Speed (US)
PAHO: Pan American Health Organization
PCR: Polymerase chain reaction
PM: Prime minister
PPI: Patient and public involvement
RCT: Randomized controlled trial
R&D: Research and development
RADx: Rapid Acceleration of Diagnostics (US)
RECOVERY: Randomised Evaluation of COVID-19 Therapy
REMAP-CAP: Randomized, Embedded, Multifactorial Adaptive Platform trial for Community-Acquired Pneumonia
RKI: Robert Koch Institute (Germany)
SAGE: Scientific Advisory Group for Emergencies (UK)
SPOR: Strategy for Patient-Oriented Research (Canada)
UBC: University of British Columbia
UK: United Kingdom
US: United States

## Footnotes

1 The comprehensive strategy described here is that of the National Institute for Health Research (NIHR) which was established by the English health department, and has a central role in England’s health research landscape, while the governments and administrations of the other three nations in the United Kingdom made their own arrangements. However, the NIHR does work alongside the other organizations, and the Medical Research Council (MRC) has a United Kingdom-wide remit. Furthermore, during the pandemic the systems have collaborated strongly, including over research priorities. Therefore, in this paper the term United Kingdom will usually be used unless the context is clearly a pre-pandemic situation or references.
