# Supplementary material for: Saving millions of lives but some resources squandered: emerging lessons from health research system pandemic achievements and challenges
Source: Health Res Policy Syst. 2022 Sep 10;20:99. doi: 10.1186/s12961-022-00883-6 (PMC9464102; doi:10.1186/s12961-022-00883-6)
Supplement: Supplementary file 1 — Additional file 1. Examples of responses to the pandemic in seven health research systems [file 12961_2022_883_MOESM1_ESM.pdf]

Saving millions of lives but some resources squandered: emerging lessons from health research system pandemic achievements and challenges.

Additional File 1: Examples of responses to the pandemic in seven health research systems

Stephen R Hanney[a*], Sharon E Straus[b], Bev J Holmes[c]

[a]Health Economics Research Group, Department of Health Sciences, Brunel University London, UK.

[b]Physician-in-chief, St Michael's Hospital, Unity Health Toronto, Toronto, Ontario, Canada.

[c]President and CEO, Michael Smith Health Research BC, Vancouver, British Columbia, Canada.

Corresponding author: [*]Stephen Hanney[a] Stephen.hanney@brunel.ac.uk

Introduction

This additional file presents the evidence collated about examples of the contributions made and challenges faced, during the SARS-CoV-2 pandemic by the health research systems (HRSs) in Australia, Brazil, Canada, Germany, New Zealand, United Kingdom (UK), and the United States (US). The reasons for selecting the seven countries, and key contextual issues, including their respective COVID-19 death (and excess death) rates, are all described in the background section of the main article. The accounts primarily cover 2020 and 2021, giving particular emphasis to the response in 2020. To create the series of accounts we identified relevant publications in three main ways.

1. We regularly scanned the emerging global literature, especially in the journals publishing the major breakthroughs in vaccines, therapies, diagnosis and policy-related research, major living systematic reviews, and regular news and opinion pieces. These journals included *Nature,* the *BMJ,* the *Journal of the American Medical Association (JAMA),* the *Lancet* and the *New England Journal of Medicine (NEJM).* Additionally some studies described aspects of the response of particular HRSs. These included the ongoing Collection of papers in *Health Research Policy and Systems*.
2. We extracted the most relevant data for our study from several rapidly conducted studies of the international research response to the pandemic, including an analysis of the total number of projects, and resources provided, by research funders globally (although full figures from some countries such as China were not obtained) [1], and an analysis of research outputs [2].
3. We drew, in a highly selective manner to meet our own requirements, on various multi-country analyses of the COVID-19 response in groups of specific countries, each of which included some, but not necessarily all of our seven countries. The respective teams conducted these studies mainly by reviewing relevant peer-reviewed and grey literature [3-7].

Also for Canada, SES drew on her experience as a senior medical academic and member of key bodies, and BJH drew on her experience with a provincial health research agency. SRH drew on material he had reviewed for the World Health Organisation (WHO) evidence synthesis on HRSs [8], and a continuing review of relevant websites.

We limited the focus and scope of the analysis because of the volume of COVID-19 research conducted, but sometimes adopted a snowballing approach once publications on a selected topic

had been identified. For each country we also searched the website of at least one major research funder or institute. Hundreds of COVID-19 projects were started in just our selected countries, and each HRS has confronted a range of challenges. Overall, we focused on literature about research that made some impact, e.g., on vaccine development and use, or non-pharmaceutical interventions (NPIs), or identification of drugs rapidly found to be effective (e.g., corticosteroids and Interleukin 6 (IL-6) receptor antagonists) or not effective as with hydroxychloroquine (HCQ). For each country we considered issues of particular expertise, relevance or concern. We also focused on efforts to promote and facilitate uptake of research evidence by policymakers and practitioners, and address the needs of Indigenous/minority populations. For each country, we also described publications that either provided context about the pandemic response of the HRS, or that contributed to understanding of the relative overall performance of the country as illustrated on Table 1 of the main paper, and/or seemed likely to provide useful lessons. Inevitably, the situation in many countries has fluctuated and the examples from specific countries might relate to a unique period of the pandemic.

Each country account is organised under three broad headings. The first two sections focus on key examples of achievements in, respectively, a) efficiently producing and synthesising useful COVID-19 evidence, and b) effectively promoting and facilitating evidence use in product development, policy and practice. The third section covers examples of challenges in first, producing and synthesising useful evidence, and second, in using local and global evidence. Finally, the third section additionally begins to identify examples of the damage that the pandemic has caused to parts of HRSs, and responses to the challenges that occurred during the pandemic. For each country we provide a separate reference list, but there is just one list of abbreviations and acknowledgements at the end.

References

1. Norton A, Bucher A, Antonio E, Advani N, Grund H, Mburu S, et al. A living mapping review for COVID-19 funded research projects: nine-month update [version 4; peer review: 2 approved]. Wellcome Open Research 2021; doi: https://doi.org/10.12688/wellcomeopenres.16259.4.
2. Kim K, Cho KT. A review of global collaboration on COVID-19 research during the pandemic in 2020. Sustainability, 2021; doi: https://doi.org/10.3390/su13147618.
3. Jasanoff S, Hilgartner SJ, Hurlbut JB, Özgöde O, Rayzberg M, CompCoRe Research Team. Comparative covid response: crisis, knowledge, politics. Interim report. 12 Jan 2021. https://compcore.cornell.edu/wp-content/uploads/2021/03/Comparative-Covid-Response_Crisis-Knowledge-Politics_Interim-Report.pdf. Accessed 20 Nov 2021.
4. Marten R, El-Jardali F, Hafeez H, Hanefeld J, Leung GM, Ghaffar A. Co-producing the covid-19 response in Germany, Hong Kong, Lebanon, and Pakistan. BMJ. 2021;372 doi: https://doi.org/10.1136/bmj.n243.
5. COVID-END. COVID-19 evidence network to support decision-making. COVID-END (mcmasterforum.org). Accessed 8 Nov 2021.
6. Han E, Tan MMJ, Turk E, Sridhar D, Leung GM, Shibuya K, et al. Lessons learnt from easing COVID-19 restrictions: an analysis of countries and regions in Asia Pacific and Europe. Lancet. 2020; doi: https://doi.org/10.1016/ S0140-6736(20)32007-9.
7. Tworek H, Beacock I, Ojo E. Democratic Health Communications during Covid-19: A RAPID Response. Vancouver: UBC Centre for the Study of Democratic Institutions; Sept2020. Democratic-Health-Communication-during-Covid_FINAL.pdf (ubc.ca) Accessed 4 May 2022.
8. Hanney SR, Kanya L, Pokhrel S, Jones TH, Boaz H. How to strengthen a health research system: WHO's review, whose literature and who is providing leadership? Health Res Policy Sys. 2020;

doi: https://doi.org/10.1186/s12961-020-00581-1.

Australia

Section 1: *Examples of achievements in production of primary and secondary research to inform development of COVID therapies, vaccines, national/local organisational responses; examples (and lessons) about links to existing strengths in the health research system*

The many studies, often rapidly funded and established [1,2] resulted in the 9th highest number of 2020 publications according to global analysis which also indicated Australia had the 3rd highest publication rate per head of population among the 12 most prolific nations (behind the UK and Italy) [3].

The University of Sydney's Edward Holmes made the first public release of the sequence of the genome of SARS-CoV-2 on behalf of his colleagues in a pre-existing consortium led from Fudan University, Shanghai [4-6]. They had submitted a full paper to *Nature* on 7 January 2020, but given the urgency, the sequence was released to a website by Holmes on 11 January [4]. *Nature* published the full paper on 3 February - it received over 4,000 citations by the end of 2021 [5]. Holmes co-authored a series of highly cited COVID-19 papers. He won the 2021 Prime Minister's (PM) Prize for Science for his *"transformative role in the scientific response to COVID-19, and his groundbreaking research into the evolution of viral diseases"* [7].

The Australian Partnership for Preparedness Research on Infectious Disease Emergencies (APPRISE) was established in 2016 with National Health and Medical Research Council (NHMRC) funding as a partnership of 13 organisations, including eight universities, to produce evidence to inform the country's ability to prepare and respond to infectious disease outbreaks. On 13 January 2020, APPRISE responded to COVID-19 by activating *"a pre-planned research platform to rapidly identify and investigate an emerging disease threat"* [8].

On 29 January 2020, researchers at the Peter Doherty Institute for Infection and Immunity, Melbourne Hospital, Victoria, some of them associated with the Victorian Infectious Diseases Reference Laboratory, claimed to be the first group to isolate SARS-CoV-2 outside of China [8,9]. In a paper submitted to *Medical Journal of Australia* on 25 February 2020, they reported conducting *"sequencing, imaging and rapid global sharing"* of the virus isolated from the patient [9]. Team members acknowledged receiving funding from APPRISE and various NHMRC sources, and acknowledged Victoria's health department as the major funder of the lab. The team also thought a reason for the success was the extensive clinical experience in the lab [9]. An early study of concomitant immune responses prior to recovery conducted at the Doherty Institute attracted global attention when published in *Nature Medicine* on 16 March 2020. The authors acknowledged a similar range of funders and the role of the platform activated by APPRISE [10].

In contrast to the flu pandemic of 2009 when researchers and NHMRC had to scramble to initiate, fund and secure ethics approval for urgent projects, NHMRC noted in 2020 *"This year, we saw the value of research preparedness and the ability of a trusted network such as APPRISE to attract additional funds"* [8]. The additional funds from NHMRC [11] and philanthropic sources supported

studies on a range of topics, including better tests for surveillance and point-of-care testing for flu and COVID-19 in nursing homes [8,11]. Sharon Lewin, Director of the Doherty Institute and chief investigator for APPRISE, explained how work conducted since 2016 to prepare for a pandemic: *"enabled us to fast track our COVID-19 related research"* [11]. The Paul Ramsey Foundation's initial AUD9m pandemic research funding in March 2020 explicitly included AUD2m support for APPRISE's COVID-19 research to develop effective responses with First Nations peoples [12]. Lewin said: *"This work will be done in partnership with Indigenous people and will be led by Indigenous researchers who are already active leaders in APPRISE"* [12]. It included studies at the University of Queensland [13].

APPRISE also contributed additional funding for the international Randomised, Embedded, Multifactorial, Adaptive Platform trial for Community-Acquired Pneumonia (REMAP-CAP), that existed prior to COVID-19 with researchers in many countries [14,15]. The international team held a meeting on COVID-19 on 23 January 2020 and by 3 March had adapted the trial to add COVID-19 treatments. Internationally it started recruiting patients on 9 March [14,16]. For patients seriously ill with COVID-19, the REMAP-CAP trial included corticosteroids and IL-6 receptor antagonists as potential repurposed therapies [17,18]. In Australia, REMAP-CAP received philanthropic (Minderoo Foundation) funding in addition to that from NHMRC and almost 20% of the 56 members of the writing committee had at least one Australian institutional affiliation [17,18]. The corticosteroid study stopped early when equipoise was lost on announcement of findings from the UK's Randomised Evaluation of COVID-19 Therapy (RECOVERY) platform trial [19]. Nevertheless, REMAP-CAP contributed the third highest weight (12%) to WHO's early living review supporting use of corticosteroid, to which some of the Australian REMAP-CAP authors also contributed [20].

The AustralaSian COVID-19 Trial (ASCOT) was established to be a large adaptive platform study including sites in New Zealand (later also in India and Nepal) for non-intensive care patients, with funding from both countries' research councils, plus others including philanthropic foundations [21]. The arms have had to be amended as a consequence of findings from studies such as RECOVERY that advanced more rapidly, e.g., HCQ was dropped [22,23].

A long established lab funded by the Commonwealth Scientific and Industrial Research Organisation contributed to animal testing of vaccines including Oxford/AstraZeneca. Developments such as global cooperation over emerging data meant progress was unusually rapid [24,25]. In addition to the various Australian organisations funding COVID-19 research [26], the University of Queensland received up to USD10.6m as early as 23 January 2020 from the international Coalition for Epidemic Preparedness Innovations (CEPI) to develop its vaccine candidate [27]. (But see section 3.)

Early in the pandemic, Australian researchers led an international study to create a core outcomes set for COVID trials. They suggested the set should include: mortality, respiratory failure, multi organ failure, shortness of breath and recovery [28]. These researchers partially overlapped with those responsible for pioneering Australian-led research that had resulted in the establishment of living (i.e., regularly updated) systematic reviews [29,30]. The living systematic review approach proved especially useful during the pandemic [31] and, sometimes with involvement of the Australian researchers, was promoted by WHO, as above [20], and others including the *BMJ* [32].

Just prior to the pandemic the living systematic review team also created the Australian Living Evidence Consortium to *"pioneer the development and deployment of a world-first, end-to-end,*

closed-loop evidence system for near real-time updating of systematic reviews and clinical practice guideline recommendations" [33]. This, too, immediately proved to be particularly valuable during the pandemic, and with funding from the federal government's Medical Research Future Fund, and Victoria's health department, led to the creation of the National COVID-19 Clinical Evidence Taskforce [34]. (See next section.)

The state level: New South Wales (NSW)

In addition to national funding bodies, state governments launched COVID-19 research programmes, e.g., in April 2020 the New South Wales (NSW) health department launched its AUS25m programme. The programme claimed that in response to a public health emergency such as the COVID-19 pandemic*, "An established and agile research infrastructure is a powerful tool in enabling rapid research production and knowledge dissemination"* [2]. The programme drew on and reflected many components of the comprehensive "*Population Health Research Strategy 2018-2022"* from NSW Health, the state's public healthcare system [35]. Therefore, in addition to projects, the COVID-19 research programme included work streams addressing the research infrastructure and process issues arising in the pandemic, such as the desirability of establishing adaptive platform trials. In recognition of the need for urgency in the pandemic, another workstream attempted to minimise unnecessary delays for COVID-19 related research in both approvals for research ethics and site specific assessments – which had been causing some delays. This workstream also created a pathway *"for efficiencies in future emergencies and business-as-usual procedures"* [2]. A further workstream aimed to expedite the translation of the COVID-19 research, measure the impact of the programme, and boost the communications of the programme's achievements.

Several of the prioritised projects assessed the COVID-19 response and health needs in different Aboriginal communities. Another project used genome sequencing to help trace the source of COVID-19 cases in the community [2]. According to the 2018-22 strategy, the Sax Institute in Sydney, NSW, received core funding from the NSW Ministry of Health [35], and a COVID-19 project built on the Institute's existing infrastructure of the 5-yearly *45 and Up* population health survey. It quickly pivoted to electronic surveys that were "co-produced" with the Ministry of Health to identify policy-relevant questions that were then sent to tens of thousands of study participants. Topics included ones directly related to COVID-19 and also *"concerns such as missed healthcare and mental health during the pandemic"* [2,36].

The 2018-22 strategy had also highlighted the importance of building capacity to undertake collaborative, or co-produced, research: *"long-term programmatic engagement between researchers and policy makers and practitioners has the greatest potential for enhancing the quality and relevance of population health research in NSW"* [35]. It specifically aimed to: *"Develop policy and practice environments that value research....* [and] *Foster research environments that promote the use of research evidence".* The strategy promoted the long-term Prevention Research Support Program aimed at supporting research infrastructure and building research capacity, including among the staff of NSW Health [35].

NSW Health's COVID-19 programme funding reflected various aspects of the strategy in "*The Emergency Response Priority Research"* programme that focused on the rapid creation of evidence that would support urgent operational work for the public health management of COVID-19 in NSW [37]. The projects were expedited between April 2020 and June 2021, and the programme

"leveraged existing research infrastructure, agreements and partnerships" [37]. As part of this, research personnel were embedded in NSW Health *"to work directly with NHS Health datasets to inform the pandemic response".* Projects funded as part of the emergency response included ones on wastewater-based epidemiology and the transmission of COVID-19 in schools [2]. Their impact on policy is described below.

Over one week in March 2020, NSW Health also established the COVID-19 Critical Intelligence Unit (CIU) to create a living evidence repository *"to inform clinical policy and clinical practice....*[It] *provides rapid and 'good enough' advice and transparency regarding the limitations of current, and often rapidly changing, evidence"* [38].

Section 2: *Examples of achievements in promoting evidence use in products, policymaking and practice; examples (and lessons) about links to existing strengths*

As early as 31 January 2020, a leading APPRISE researcher reported they were developing research to share data with government departments and agencies to support decision-making during the COVID-19 emergency [39]. A report in May 2020 in the *NEJM* from the NSW Ministry of Health identified how Australia had achieved an unusual national consensus on COVID-19 policies. Furthermore, expert committees had played a key role meeting daily and advising bodies that created policies, recommended legislation, and implemented laws related to COVID-19 [40]. From February 1, the Australian government increasingly tightened its border-control policies, and by mid-March it restricted entry for all foreigners.

Reports in the international press from November 2020 made similar points in highlighting factors behind the policy success in limiting the number of COVID-19 deaths as shown on Table 1 of the main paper, i.e., listening to scientific advice, and strong consensus leadership [41,42]. In December 2020, the Australian Academy of Health and Medical Sciences, claimed *"by any global measure the response to date has been a spectacular success"*, and it called for continued support for research to deliver *"the knowledge and tools required to tackle the pandemic"* [43].

Reports in December 2020 also suggested a generally high level of public trust in experts, and ministerial support for their input, with a leading role for previously marginalised Indigenous public health experts [44]. The Association of Australian Medical Research Institutes (AAMRI) claimed in November 2021: "*It is no accident that Australia has been able to mount such a strong response during this pandemic; it has been made possible by decades of investment in building up our health and medical research capacity"* [45].

Researchers from the APPRISE project at the University of Queensland claimed in September 2021 that despite the global picture of Indigenous communities suffering disproportionate infections and deaths, *"until very recently Aboriginal and Torres Strait Islander peoples had not recorded a single fatality"* [13]. The Indigenous communities had effectively taken the initiative to protect their communities from COVID-19, and researchers were trying to help identify lessons, but vaccine hesitancy became a problem (see also section 3).

The Australian Technical Advisory Group on Immunisation provided important scientific advice to the federal Minster of Health on vaccines and also liaised with researchers [46]. The National Centre for Immunisation Research and Surveillance is co-funded by the Australian and NSW Governments,

and as the leading body for immunisation research in Australia undertook COVID-19 research on issues such as vaccination coverage and serosurveillance and providing scientific technical support to the Advisory Group [47].

At the state level, the NSW government's response built on previous pandemic planning that had been informed by lessons from severe acute respiratory syndrome (SARS) and H1N1 influenza. As early as January 20, *"NSW Health opened its Public Health Emergency Operations Centre... to coordinate case finding, contact tracing, outbreak control, communications, and other preventive actions"* [40].

The COVID-19 research programme from the NSW's health ministry provides a strong example of effective co-production of evidence. Authors from the health ministry reported: "*the agile response of highly skilled and experienced researchers in close partnership with policy makers through the Emergency Response Priority Research workstream has ensured health decision makers have the best possible local evidence on which to base operational decisions"* [37]. The evaluation of the NSW COVID-19 research programme emphasised how pre-existing relationships between academics and policymakers were successfully leveraged through creating a new structure for collaboration. It quoted the NSW Chief Health Officer as saying: *"Some researchers have been able to be very nimble and can thrive in these environments....The researchers are given access to our data, it's efficient, it's evidence-based, it's a win-win and a new way of working with researchers"* [2].

The evaluation also included the early stages of a formal research impact assessment [2]. In part, this assessment was organised using the Framework for Assessing the Impact of Translational health research, itself developed earlier in NSW [48]. The evaluation described early impact and a plan for a longer-term assessment, in particular of the sewage surveillance programme. That study validated the methods being used by Sydney Water, with which there was a long-standing research partnership. The findings enabled NSW Health *"to target messaging and testing to high-risk areas"* and were also used to manage border restrictions [2,37]. In the longer term, it was intended that some assessment would be attempted of the benefits to health and the economy of being able to perform the tests and translate findings to policymakers and the public in ways "*likely to have prevented additional community transmission"* and that helped inform the easing of restrictions [2].

The study on COVID-19 transmission in schools involved researchers from the NSW-based National Centre for Immunisation Research and Surveillance working with the state's Ministry of Health and Department of Education [2]. It not only resulted in a publication in *Lancet Child & Adolescent Health* that has received considerable international attention [49], but also informed decision making and *"helped shape policy around higher risk activities to help reduce transmission in educational settings"* [37].

Overall, NSW had a lower death rate in 2020/21 even than the low figures for Australia as a whole [50]. The paper describing the CIU claimed that as at Autumn 2020 there had been no shortage of intensive care beds, there had not been shortages of personal protective equipment and there had been a very small number of cases in care facilities. It further claimed: *"The CIU has played a part in these achievements....The CIU represents one of these opportunities to innovate: an organisational and operating model for informed decision making that has potential applications in other systems and for a post-pandemic future in NSW, supporting the goal of establishing learning health care*

systems"
[38].

As noted in section 1, building on their living systematic reviews [29,30], the Australian Living Evidence Consortium and Cochrane Australia worked with 31 (later 32) peak professional bodies in March 2020 to create The National Taskforce [34,51]. National living evidence-based guidelines were published two weeks later, and revised over 70 times by December 2021. NHMRC support was vital to ensure standards. This success showed creating living guidelines was feasible [34,51]. Taskforce members surveyed practitioners caring for COVID-19 patients, and also interviewed some. The vast majority reported they found the guidelines highly relevant; over 50% *"had used the guidelines to support their own clinical decision making... amongst an overwhelming morass of evidence and opinions during the COVID-19 pandemic, the guidelines have been a reliable, united source of evidence-based advice"* [52].

A prospective study of policies on, and use of, COVID-19 therapies in the health service in Australia from May to December 2020 examined the role of literature and guidelines. The RECOVERY trial was most frequently referenced as a factor influencing policy change, with the National Living Guidelines referenced by 26% of respondents reporting policy changes. The prospective study highlighted the *"importance of evidence-based, iterative guidelines to guide our response to the current COVID-19 pandemic"* [53].

Section 3: *Examples of challenges in: 1. production of evidence; 2. use of evidence; and 3. sustaining the research system; examples of lessons to address challenges*

<u>*Challenges in production of evidence*</u>

Analysis published in May 2021 [1] of 56 COVID-19 trials registered in Australia found: none had full, publicly available protocols; only one included all core outcomes (see section 1); many were under-powered with 80% having no plans to share data; many faced recruitment issues (the policy successes resulted in relatively few COVID cases in Australian hospitals); and duplication was common, e.g., six trials of HCQ *"may have put many patients unnecessarily at risk."* Such problems also occurred elsewhere, e.g., there were over 250 HCQ trials globally [31] which often impaired recruitment to other trials [31]. Nevertheless, greater research coordination was called for in Australia, with a bigger role for a trial registry: *"The COVID-19 pandemic presents a unique opportunity to improve collaborative infrastructure and methodologies"* [1].

Also in mid-2021, researchers linked to ASCOT called for a streamlined approach to funding prioritised pandemic projects, more rapid ethical/governance approvals of protocols and *"A small number of national platforms in Australia, similar to RECOVERY in the UK, as the principal vehicle for publicly funded trials"* [22]. In relation to approvals they pointed out that while Australia had a National Mutual Acceptance scheme for ethical approval of multicentre clinical trials, *"the requirement for governance approvals at each individual site creates substantial delays and critical roadblocks"* [22]. The ASCOT project website regularly updated the trial's recruitment figures, and it only reached its 1,000th patient in September 2021 after extending the trial to India and Nepal [21].

The promising COVID-19 vaccine being developed by an Australian company and researchers at the University of Queensland [27] had to be abandoned following problems that became apparent

during the trials (but trial participants' health was not at risk) [54]. The Government had previously agreed to buy 51 million doses.

Challenges in use of evidence

Addressing the Delta variant later in 2021, and then Omicron, proved more challenging than the first 18 months. Some tensions over the most appropriate policies that had been noted in the December 2020 report [44] became more prominent. As 2021 ended, deaths and cases were on an upward trajectory, despite, overall, still being considerably lower than most countries [55]. There were fears that policymakers were not sufficiently considering the concerns of community leaders and researchers about the dangers of ending COVID-19 restrictions before high levels of vaccination had been achieved in the Aboriginal communities [56]. (There was a dramatic surge of cases across many parts of the country in 2022 associated with the spread of Omicron, the easing of public health restrictions and the opening of state and national boundaries, but while deaths increased from their very low level, they remained low by international standards.)

Challenges in sustaining the research system

Despite its positive comments noted above, the AAMRI report also noted some concerns about Australia's medical research, some of which were long-term issues exacerbated by the pandemic. Concerns included a lack of national coordination of priorities or sufficient identification of needs, problems with job security and gender inequity and, it claimed, *"Collaboration and cooperation between research and healthcare delivery remains fragmented*" [44]. It called for all stakeholders in the medical research sector to come together and develop *"A National Health and Medical Research Strategy"* [44].

Section 4: *Summary*

The high rates of publications and the direct and demonstrated evidence impacts on public health policy illustrate that the research environment in Australia generally supported a high-quality response to COVID-19. The coordination provided through the pandemic preparedness APPRISE programme was particularly important in ensuring a rapid response by the HRS, including attention to the needs of the Indigenous population. The policy successes in controlling the virus meant, however, Australia had fewer cases to enter into clinical trials than most countries. This exacerbated problems with a lack of national coordination of clinical trials resulting in too many small, underpowered trials of re-purposed drugs. Nevertheless, the pioneering development of national living clinical guidelines was important, and, as more generally in the Australian response, the success was built upon long term investment in research and research capacity and infrastructure. In particular, in NSW the health research strategy from 2018 was mirrored in the comprehensive COVID-19 research programme that, especially with its elements of co-production of research and mobilising existing long-term partnerships, has been evaluated as meeting the needs of health system leaders. It has been proposed that across Australia, the lessons learnt from the pandemic response, some of which reflect more long-term concerns about job security and equitable treatment for all research staff, should be harnessed to build a stronger national health research strategy. This could cover areas including further strengthening the relationship between the research systems and the healthcare/policy systems, committing to agile mechanisms for greater national coordination and also for identification and funding of priority issues.

References

1. Seidler AL, Aberoumand M, Williams JG, Tan A, Hunter KE, Webster A. The landscape of COVID-19 trials in Australia. The research response in Australia has been rapid, but better coordination is imperative. Med J Aust. 2021; doi: https://doi.org/10.5694/mja2.51148
2. Centre for Epidemiology and Evidence. NSW Health COVID-19 Research Program Impact Evaluation Report 2021. Sydney: NSW Ministry of Health; 2021 covid-impact-evaluation-interim.pdf (nsw.gov.au) Accessed 5 May 2022.
3. Kim K, Cho KT. A review of global collaboration on COVID-19 research during the pandemic in 2020. Sustainability. 2021; doi: https://doi.org/10.3390/su13147618.
4. Farrar J, Ahuja A. Spike - The virus vs the people. The inside story. London: Profile Books; 2021.
5. Wu F, Zhao S, Yu B, Chen Y-M, Wang W, Song Z-G, et al. A new coronavirus associated with human respiratory disease in China. Nature. 2020;579:265–9. https://doi.org/10.1038/s41586-020-2008-3
6. Zhang YZ, Holmes EC. A genomic perspective on the origin and emergence of SARS-CoV-2. Cell. 2020; doi: 10.1016/j.cell.2020.03.035.
7. Salleh A. Eddie Holmes wins Prime Minister's Prize for Science for work on COVID-19 and viral evolution. ABC Science, 3 Nov 2021**.** Eddie Holmes wins Prime Minister's Prize for Science for work on COVID-19 and viral evolution - ABC News. Accessed 18 Jan 2022.
8. National Health and Medical Research Council (NHMRC). Australian partnership for preparedness research on infectious disease emergencies. 27 Oct 2020. Australian Partnership for Preparedness Research on Infectious Disease Emergencies | NHMRC. Accessed 12 Dec 2021.
9. Caly L, Druce J, Roberts J, Bond K, Tran T, Kostecki R, et al. Isolation and rapid sharing of the 2019 novel coronavirus (SAR-CoV-2) from the first patient diagnosed with COVID-19 in Australia. Med J Aust. 2020; doi: 10.5694/mja2.50569.
10. Thevarajan I, Nguyen THO, Koutsakos M, Druce J, Caly L, van de Sandt CE, et al. Breadth of concomitant immune responses prior to patient recovery: a case report of non-severe COVID-19. Nat Med, 2020; doi: https://doi.org/10.1038/s41591-020-0819-2.
11. APPRISE. Fast-tracked funding for the COVID-19 pandemic to support critical research areas. APPRISE, 19 March 2020. Fast-tracked research funding for the COVID-19 pandemic (apprise.org.au). Accessed 11 Jan 2022.
12. APPRISE. Paul Ramsay Foundation commits $2m to support APPRISE COVID-19 research with First Nations peoples. APPRISE, 23 March 2021. Paul Ramsay Foundation – APPRISE Indigenous COVID-19 research. Accessed 12 Jan 2022.
13. Fredericks B, Ward J, McAvoy, Combo T, Spierings S, Bradfield A, et al. Innovative research explores responses to COVID-19 among Indigenous communities in Brisbane. APPRISE, 14 Sept 2021 – republished from Croakey Health Media, 8 Sept 2021. Responses to COVID-19 among Indigenous communities in Brisbane (apprise.org.au). Accessed 12 Jan 2022.
14. National Health and Medical Research Council. Which treatments will save lives in ICU? 19 May 2020. Which treatments will save lives in ICU? | NHMRC. Accessed 12 Dec 2021.
15. REMAP-CAP. REMAP-CAP: A randomised, embedded, multi-factorial, adaptive platform trial for community-acquired pneumonia. REMAP-CAP Trial (remapcap.org). Accessed 7 Jan 2022.

16. Goossens H, Derde l, Horby P, Bonten M. The European clinical research response to optimise treatment of patients with COVID-19: lessons learned, future perspective, and recommendations. Lancet Infect Dis. 2021; doi: https://doi.org/10.1016/S1473-3099(21)00705-2.
17. The Writing Committee for the REMAP-CAP Investigators. Effect of hydrocortisone on mortality and organ support in patients with severe COVID-19: The REMAP-CAP COVID-19 corticosteroid domain randomized clinical trial. JAMA. 2020; doi:10.1001/jama.2020.17022.
18. The REMAP-CAP Investigators. Interleukin-6 receptor antagonists in critically ill patients with Covid-19. N. Engl. J. Med. 2021;384:1491-1502.
19. RECOVERY. Low-cost dexamethasone reduces death by up to one third in hospitalised patients with severe respiratory complications of COVID-19. Oxford University News Release; University of Oxford; 16 June 2020. recovery_dexamethasone_statement_160620_v2final.pdf (recoverytrial.net). Accessed 12 Jan 2022.
20. The WHO Rapid Evidence Appraisal for COVID-19 Therapies (REACT) Working Group. Association between administration of systemic corticosteroids and mortality among critically ill patients with COVID-19. A meta-analysis. JAMA. 2020; doi: https://dx.doi.org/10.1001%2Fjama.2020.17023
21. ASCOT. AustralaSian COVID-19 trial. https://www.ascot-trial.edu.au. Accessed 10 Dec 2021.
22. Bowen AC, Tong SYC, Davis JS. Australia needs a prioritised national research strategy for clinical trials in a pandemic: lessons learned from COVID-19. Med J Aust. 2021; doi: https://doi.org/10.5694/mja2.51143.
23. Health Research Council. Update on COVID-19 clinical trials. HRC, New Zealand, 17 Feb 2021. https://hrc.govt.nz/news-events/update-new-zealands-rapid-response-covid-19-clinical-trials. Accessed 12 Jan 2022.
24. Hanney SR, Wooding S, Sussex J, Grant J. From COVID-19 research to vaccine application: why might it take 17 months not 17 years and what are the wider lessons? Health Res Policy Sys. 2020; doi: https://doi.org/10.1186/s12961-020-00571-3.
25. Mao F. Coronavirus: Australian scientists begin test of potential vaccines. BBC. 2 Apr 2020. https://www.bbc.co.uk/news/world-australia-52130402. Accessed 29 Dec 2021.
26. Norton A, Bucher A, Antonio E, Advani N, Grund H, Mburu S, et al. A living mapping review for COVID-19 funded research projects: nine-month update [version 4; peer review: 2 approved]. Wellcome Open Research 2021; doi: https://doi.org/10.12688/wellcomeopenres.16259.4.
27. Coalition for Epidemic Preparedness Innovations (CEPI). CEPI to fund three programmes to develop vaccines against the novel coronavirus, nCoV-2019. CEPI. 23 January 2020. CEPI to fund three programmes to develop vaccines against the novel coronavirus, nCoV-2019 – CEPI Accessed 7 June 2020.
28. Tong A, Elliott JH, Azevedo LC, Baumgart A, Bersten A, Cervantes L, et al. Core outcomes set for trials in people with coronavirus disease 2019. J Crit Care Med. 2020; doi: 10.1097/CCM.0000000000004585.
29. Elliott JH, Turner T, Clavisi O, Thomas J, Higgins JPT, Mavergames C, et al. Living systematic reviews: an emerging opportunity to narrow the evidence-practice gap. PLoS Med. 2014; doi: https://doi.org/10.1371/journal.pmed.1001603.
30. Elliott JH, Synnot A, Turner T, Simmonds M, Akl EA, McDonal S, et al. Living systematic review: 1. Introduction-the why, what, when, and how. J Clin Epidemiol. 2017; 91:23-30.

31. Pearson H. How COVID broke the evidence pipeline. The pandemic stress-tested the way the world produces evidence — and revealed all the flaws. Nature. 2021; doi: https://doi.org/10.1038/d41586-021-01246-x.
32. Siemieniuk RAC, Bartoszko JJ, Ge L, Zeraatkar D, Izcovich A, Kum E, et al. Drug treatments for covid-19: living systematic review and network meta-analysis. BMJ. 2020; doi: https://doi.org/10.1136/bmj.m2980
33. Australian Living Evidence Consortium. About Us. About us — Australian Living Evidence Consortium. Accessed 17 Nov 21.
34. Tendal B, Vogel JP, McDonald S, Norris S, Cumpston M, White H, et al. Weekly updates of national living evidence-based guidelines: methods for the Australian living guidelines for care of people with COVID-19. J Clin Epidemiol. 2021; DOI: 10.1016/j.jclinepi.2020.11.005
35. Centre for Epidemiology and Evidence. Population Health Research Strategy 2018–2022. Population and Public Health Division. Sydney: NSW Ministry of Health, 2019. research-strategy-2018-2022.pdf (nsw.gov.au) Accessed 5 May 2022.
36. Sax Institute. New COVID survey data for researchers: coming soon. Sax Institute. Dec 2021. https://www.saxinstitute.org.au/news/new-covid-survey-data-for-researchers-coming-soon/?highlight=covid Accessed 7 May 2022.
37. Campbell D, Edwards B, Milat A, Thackway S, Whittaker E, Goudswaard L, et al. NSW Health COVID-19 Emergency Response Priority Research Program: a case study of rapid translation of research into health decision making. Public Health Res Pract. 2021;31(4):e3142124. https://doi.org/10.17061/phrp3142124
38. Levesque J-F, Sutherland K, Watson DE, Currow DC, Bolevich Z, Koff E. Learning systems in times of crisis: the Covid-19 Critical Intelligence Unit in New South Wales, Australia. NEJM Catalyst. 2020. https://catalyst.nejm.org/doi/full/10.1056/CAT.20.0542
39. Australian Research Data Commons. Sharing research data in a timely manner can support public health response to an infectious disease emergency. Sharing research data in a timely manner can support public health response to an infectious disease emergency - ARDC. Accessed 13 Dec 21.
40. McAnulty JM, Ward K. Suppressing the epidemic in New South Wales. NEJM. 2020;382(21):e74. https://www.nejm.org/doi/10.1056/NEJMc2011592
41. Smyth J. How Australia brought the coronavirus pandemic under control. Financial Times, 13 Nov 2020 How Australia brought the coronavirus pandemic under control | Financial Times (ft.com). Accessed 9 Nov 2021.
42. Patrick O. Australia has almost eliminated the coronavirus — by putting faith in science. Washington Post, 5 Nov 2020. Australia almost eliminated the coronavirus by putting faith in science - The Washington Post. Accessed 9 Nov 21.
43. Australian Academy of Health and Medical Sciences. Maintaining strong foundations and building resilience: planning Australia's path through the COVID-19 pandemic. 15 Dec 2020. AAHMS_COVID-19_2021_report_15-Dec-2020.pdf. Accessed 12 Jan 2022.
44. Baskin J, Raman S, Grant W. Australia. In: Jasanoff S, Hilgartner SJ, Hurlbut JB, Ozgode O, Rayzberg M. Comparative Covid response: crisis, knowledge, politics. Interim report. 12 Jan 2021. https://compcore.cornell.edu/wp-content/uploads/2021/03/Comparative-Covid-Response_Crisis-Knowledge-Politics_Interim-Report.pdf. Accessed 12 Jan 2022.
45. Association of Australian Medical Research Institutes. Australia's missing link: a national health and medical research strategy. AAMRI, Nov 2021. https://www.aamri.org.au/wp-

content/uploads/2021/11/Australias-Missing-Link-A-National-Health-and-Medical-Research-Strategy.pdf. Accessed 12 Jan 2022.
46. Australian Technical Advisory Group on Immunisation. Australian Government Department of Health. Australian Technical Advisory Group on Immunisation (ATAGI) | Australian Government Department of Health. Accessed 15 Dec 21.
47. National Centre for Immunisation Research and Surveillance. COVID-19. https://www.ncirs.org.au/our-work/covid-19 Accessed 5 May 2022.
48. Searles A, Doran C, Attia J, Knight D, Wiggers J, Deeming S, et al. An approach to measuring and encouraging research translation and research impact. Health Res Policy Sys. 2016;14:60. doi:10.1186/s12961-016-0131-2.
49. Macartney K, Quinn HE, Pillsbury AJ, Koirala A, Deng L, Winkler N, et al. Transmission of SARS-CoV-2 in Australian educational settings: a prospective cohort study. Lancet Child Adolesc Health 2020; 4(11): 807-16.
50. Australian Bureau of Statistics. Measuring Australia's excess mortality during the COVID-19 pandemic. Australian Bureau of Statistics. 30 March 2022. https://www.abs.gov.au/articles/measuring-australias-excess-mortality-during-covid-19-pandemic Accessed 12 April 2022.
51. National COVID-19 Clinical Evidence Taskforce. (2021 version 45.2). Australian guidelines for the clinical care of people with COVID-19. https://covid19evidence.net.au/. Accessed 16 Nov 2021.
52. Millard T, Elliott JH, Green S, Molton JS, Wurzel D, Hughes CM, et al, on behalf of the National COVID-19 Clinical Evidence Taskforce. Awareness, value and use of the Australian living guidelines for the clinical care of people with COVID-19: an impact evaluation. J Clinical Epidemiology. 2021: doi: https://doi.org/10.1016/j.jclinepi.2021.11.035.
53. Smith EL, Gwee A, Robert JA, Molton JS, Wurzel D, Hughes, CM, et al. Prospective study of policies and use of therapies for COVID-19 amongst Australian health services during 2020. Internal Med J. 2021. https://doi.org/10.1111/imj.15510.
54. BBC. Covid: Australian vaccine abandoned over false HIV response. BBC, 11 Dec 2020. Covid: Australian vaccine abandoned over false HIV response - BBC News. Accessed, 28 Dec 2021.
55. Worldometer: COVID live update: 236,408,442 cases and 4,826,759 deaths from the Coronavirus. COVID Live Update: 236,408,442 Cases and 4,826,759 Deaths from the Coronavirus - Worldometer (worldometers.info). Accessed: 1 Jan 2022, for data on 31 Dec 2021.
56. Komesaroff PA, Chee DA, Boffa J, Kerridge I, Tilton E. COVID-19 restrictions should only be lifted when it is safe to do so for Aboriginal communities. Intern J Med. 2021; doi: https://doi.org/10.1111/imj.15559.

Brazil

Section 1: *Examples of achievements in production of primary and secondary research to inform development of COVID therapies, vaccines, national/local organisational responses; examples (and lessons) about links to existing strengths in the health research system*

As of 1 March 2021, 26 Brazilian randomised controlled trials (RCTs) of drug treatments for COVID-19 had made sufficient progress to be included in the 4th edition of the BMJ living systematic review first published in 2020, but regularly updated. This was the joint 3rd highest number (with US) for any nation (behind China and Iran) [1]. Overall, Brazil had many COVID-19 projects: according to a cross-country analysis it had the highest number for any low- or middle-income country (although full data were not obtained from China) [2].The National Commission for Research Ethics in Brazil reported it had approved 874 COVID-19 studies by June 2021 [3]. Commenting on the response in just the early months, Rosa et al., 2021 said: *"this response was only possible due to decades of investment in research, development and innovation in Brazil"* [4]. In addition to drawing on existing capacity, there was a recognition that the urgency meant the processes of research ethics had to be accelerated and COVID-19 protocols received priority with the National Commission giving approval to 501 protocols by the 25th June 2020
[5].

Two relevant Brazilian federal agencies made early specific COVID-19 research calls. In April 2020 the National Council for Scientific and Technological Development provided USD9.2m for proposals on topics including COVID-19 treatments, vaccines, diagnosis, pathogenesis and natural history of the disease, prevention, and control [6]. The call of up to USD20.3m from the Coordination for the Improvement of Higher Education and Personnel of the Ministry of Education consisted of three separate lines launched at two different dates in April 2020. The USD29.5m from these two combined represented *"around 77% of the total amount for early COVID-19 research funding"* [6]. The rest came from 11 of the 27 states' publicly funded research foundations, with the largest amount being USD4.6m from the Rio de Janeiro State Research Support Foundation for a wide-ranging programme of COVID-19 research. One of its three lines was for the funding of up to six research networks [6].

Physician scientists collaborated to create the Coalition COVID-19 Brazil initiative that, it was claimed in December 2020 by Zimerman et al, *"encompasses more than 70 centres around the country and has been leading 11 randomised clinical trials with more than 5,000 participants. Brazil is a developing country....for which this level of coordination is unprecedented. In fact, rarely has it been achieved in developed countries"* [7]. The Coalition's corticosteroid RCT, conducted in 41 intensive care units, was halted early because of results from the RECOVERY trial. But it was still published in *JAMA,* cited almost 400 times, and also contributed to the evidence showing the effectiveness of dexamethasone [8]. In the study's primary outcome the steroid significantly increased the number of ventilation-free days. The findings for one of the study's secondary outcomes showed a small reduction in mortality that while not significant in itself, contributed 19% weight (the second highest contribution after RECOVERY) to an early WHO review supporting the use of corticosteroids, which was conducted by a team including four of the Brazilian researchers [9].

The Coalition's HCQ trial by Cavalcanti et al., 2020 [10], was published in the *NEJM* and had been conducted in 55 participating hospitals and mainly funded by the hospitals and research institutes. It showed that for mild or moderately ill hospitalised COVID-19 patients, HCQ, with or without azithromycin, did not improve clinical status. While the Coalition's study contributed just 1.1% weight to the largest early meta-analysis by Axfors et al., 2021 [11], it was of considerable importance in debates about HCQ. This is because by far the largest trials (RECOVERY, and WHO's Solidarity trial, which was a pre-print at that stage, but later published [12-14]) used a dose of HCQ that some thought was too high, despite the trialists' confidence in the advice they had received from experts that was reflected in a subsequent publication [15,16]. Therefore, Cavalcanti et al., 2020, was a major part of Axfors et al.'s separate analysis of the studies that had used a lower dosage which still found HCQ did not improve outcomes [11]. Further, the Coalition's trial of azithromycin showed it, too, did not improve clinical outcomes for COVID-19 patients [17].

In addition to the Coalition's trials, Brazilian researchers played an important part in various international platform trials – sometimes having the key role. The Ministry of Health's Oswaldo Cruz Foundation (Fiocruz) in Rio de Janeiro led Brazil's arm of WHO's Solidarity platform trial, with 12 other sites across Brazil. Solidarity found none of the four repurposed drugs tested was effective for COVID-19 [14]. The TOGETHER platform trial was led by Pontifícia Universidade Católica de Minas Gerais in Belo Horizonte, Brazil, and McMaster University, Hamilton, Canada, funded by North American philanthropists and conducted originally in 11 clinical sites across Brazil. Its study of the antidepressant fluvoxamine as an early treatment for COVID-19 patients with a known risk factor for progression to severe disease was published online in the *Lancet Global Health* in October 2021 [18]. It showed the treatment reduced the need for hospitalisation in COVID-19 patients who, given the recruitment period, were primarily unvaccinated. Reliable reports in Autumn 2021 that another arm of TOGETHER would show no benefit from the use of ivermectin [19], were confirmed in the trial's report in the *NEJM* in 2022 that attracted considerable attention [20].

Brazil had the most sites in the Canadian-funded Antithrombotic Therapy to Ameliorate Complications of COVID-19 (ATTACC) adaptive trial that combined with two other platform trials in an international trial to which Brazil enrolled the second highest number of patients overall. This showed in non-critically ill patients with COVID-19, an initial strategy of therapeutic-dose anticoagulation with heparin increased probability of survival to hospital discharge [21]. Brazilian hospitals, centres and researchers contributed to other successful international trials, e.g., ones led by companies from the US on developing a monoclonal antibody (mAb) therapy [22], and also anti-virals [23-25].

Research teams in Brazil also met the requirements necessary for trials of COVID vaccines by Oxford/ AstraZeneca and a Chinese company Sinovac Biotech. The trials included technology transfer agreements with Fiocruz and Butantan Institute, at São Paulo, respectively - see next section [4]. Sarah Gilbert, who led development of the Oxford vaccine, said Brazil was chosen because: *"we knew all the people already, we knew they would be able to conduct high-quality clinical trials because they already had the infrastructure and experience"* [26].

Both institutes continued further studies. Between February and April 2021 Butantan vaccinated almost an entire Brazilian city, Serrana, using the Chinese developed vaccine it had helped test. Cases fell by 80% and deaths by 95% [27]. This groundbreaking study attracted international

attention [28]. A study by institutions across Brazil and Scotland of waning effectiveness after two vaccine doses was funded by Fiocruz (along with the UK MRC, the Scottish Government, and others). It reported in the *Lancet* in December 2021 that waning protection became evident within three months of the second dose [29].

The Pan American Health Organization (PAHO) selected Fiocruz as one of two Latin American centres it would fund to develop and produce mRNA-based vaccines. It was selected because of its previous record on manufacturing vaccines and because it had already made promising advances in developing an innovative mRNA vaccine against COVID-19 [30]. Fiocruz also led many epidemiological projects in Brazil [4].

Section 2: *Examples of achievements in promoting evidence use in products, policymaking and practice; examples (and lessons) about links to existing strengths*

The vaccine trials contributed to the regulatory approval and use of both AstraZeneca and Sinovac vaccines in Brazil [4]. On its website, Fiocruz stated that in relation to COVID-19 it would work with the Ministry of Health and that with a *"tradition of more than 70 years in the production of vaccines, the Foundation has been committed to maintaining efforts in this field...and emphasizing the importance of the Unified Health System (SUS) as the basis for sustaining the development, production and future national distribution of a vaccine for the disease*"[31]. The technology transfer agreement meant that Fiocruz went on to manufacture the Oxford/ AstraZeneca vaccine, called Vaxzevria in Brazil, as well as conducting the policy-relevant research, described above, on the need for boosters [29].

Butantan manufactured Coronavac and its implementation study was a major success [27,28]. By analysing community protection it addressed a key policy-relevant issue identified by the Lancet Commission on COVID-19 Vaccines which took a global perspective and stated *"Evidence of community protection is crucial; it could inform government vaccination strategy and policy around ancillary protective measures"* [32]. An international research leader from Brazil, Cesar Victora, and colleagues used the Butantan study in Serrana as part of the evidence supporting their pioneering analysis of the benefits of the rapid scaling up of the Brazilian vaccination programme by the highly-regarded public healthcare system. They estimated the programme resulted in over 40,000 fewer deaths of Brazilians aged 80+ by May 2021 [33].

The Monitorcovid-19 project at Fiocruz provided data and allowed the development of predictive models for monitoring of the pandemic by public health managers and civil society [34]. In mid-March 2020, multi-disciplinary collaboration between universities/ research institutes/ companies in São Paulo developed and produced molecular diagnostic tests [35].

There were also efforts to accelerate the translation of basic research into medical devices such as the VESTA Face Respirator which sought to use chitonase-based nanotechnology, manufactured in Brazil, in the filter element to reduce COVID-19 infection. A partnership between academic researchers, a public laboratory and a private company made rapid progress with the patent approval granted in April 2021 for a *"dynamic respirator with multifunctional properties to prevent infectious diseases with self-cleaning and drug delivery protective properties"*, and the technology transfer to industry took place in December 2021 [Private Communication from Mário Fabrício Fleury Rosa, 25 April 2022].

Various Brazilian teams rapidly produced clinical guidelines - 33 by June 2020 - aimed at producing *"clear and concise recommendations ...to facilitate decision making"* [36], but see next section. One member of the Brazilian Coalition trial team which had studied dexamethasone [8], also contributed to the first version of WHO's living guideline which focussed on the effectiveness of corticosteroids [37].

Communicating science-based public health messages, and the role of research, could be challenging given the attitude of the President (see next section), but one of PAHO's various initiatives involved working creatively with Brazilian Post to promote messages through a series of stamps [38].

There was great variation in how far local policymakers made evidence-informed decisions, but one study showed there were on average fewer deaths and hospitalisations in municipalities with a female mayor, and *"more weight to scientific advice among female leaders may also explain why they enforce more NPIs"* [39].

Section 3: *Examples of challenges in: 1. production of evidence; 2. use of evidence; and 3. sustaining the research system; examples of lessons to address challenges*

Challenges in production of evidence

Despite the rapid availability of some public funding for COVID-19 research, fewer than 18% of 789 COVID projects identified in May 2020 as being led by public universities and research institutes appear to have "received funding" [4]. Drug trials, as well as patient care, across Latin America, including Brazil, were hindered by large number of patients having taken what were then unproven COVID-19 treatments such as ivermectin. A leading researcher was quoted in *Nature* in October 2020 as saying: "*What we're having is a populist treatment, instead of an evidence-based treatment"* [40]. As elsewhere [41], many of the rapidly produced guidelines were found to be of poor methodological quality [36].

Challenges in use of evidence

The adoption and promotion of chloroquine and HCQ by the Brazilian Government, including through its Ministry of Health, provides a major example of the political pressure to act in the absence of the usually required research evidence (or subsequently to ignore the evidence when it became available). President Bolsonaro replaced a health minister who seemed to oppose recommending these drugs with an army general; immediately the Ministry of Health implemented a protocol for the use of the drugs. An article analysing the available evidence and the policymaking concluded: *"In addition to being ineffective and inefficient, the line of action adopted by the Brazilian federal Government goes against the legal framework of the Unified Health System (SUS), since it values decisions based on epidemiological knowledge and actions that prioritize disease prevention and health promotion"* [42 – translated].

The very high number of cases and death rate in Brazil was blamed by *Nature* on the failure of leaders to follow the evidence: "*By sidelining their scientists, the governments of Brazil and India have missed out on a crucial opportunity to reduce the loss of life*" [43]. Similarly, a *Lancet* editorial claimed *"perhaps the biggest threat to Brazil's COVID-19 response is its president, Jair Bolsonaro*" [44]. An analysis of the use of evidence in policymaking suggested Presidents Trump and Bolsonaro

"took a political approach to the pandemic... Both leaders repeatedly resisted recommendations made by scientific experts" [45].

In Brazil, critics of the President also pointed to his devaluation of expertise and scientific evidence, and creation of mistrust through *"raising doubts about previously successful policies, especially mass vaccination"* [46]. Others claimed: *"President Jair Bolsonaro's administration has publicly undermined science while refusing to implement protective national lockdowns and spreading misinformation"* [47]. In Autumn 2021 a panel of the Brazilian senate recommended charging President Bolsonaro with crimes against humanity for his handling of COVID-19 [48].

Brazil's overall score on the Global Health Security (GHS) Index in 2019, described in Table 1 of the main paper, put it in 22nd position out of 195 - ahead of many high-income countries - and on the item *"Rapid response and mitigation of the spread of an epidemic"* it was ranked 9th – higher than Australia, Canada, Germany or New Zealand [49]. Some of the factors behind this might be linked to the comment from Marcia Castro, a Brazilian at Harvard's School of Public Health and second author on the Victora et al. paper [33], that *"Brazil could have given a lesson to the world on how to respond to a pandemic based on how it handled its response to HIV/AIDS and Zika epidemics"* [50]. She also described how Brazil had an immunisation programme that had previously worked well. However, President Bolsonaro's COVID-19 response was a failure of leadership because he and *"Donald Trump were similar in their denial of how serious the virus was and their denial of science"* [50]. Despite the *"failure of leadership"* from the President, many governors and mayors across Brazil did what they could, but inevitably they became entangled in disputes with the federal government.

In the December 2021 GHS Index Brazil had dropped down the rankings from 22nd in 2019 to 43rd [51].

Challenges in sustaining the research system

The analysis of early COVID-19 research funding predicted in mid-2020 that the economic crisis was *"likely to have a strong effect on scientific activities, such as massive drops in funding, scientific publications, patents, and qualified human resources"* [6]. The budgetary pressures on Brazilian researchers were experienced across the spectrum of the research system, with a major blow in October 2021 when the Government backtracked on a promise of more money after years of cuts [52].

Summary

Having built its health research capacity over many years, Brazil, a middle income country, was in the position to rapidly conduct important COVID-19 related research [20]. This was facilitated by many Brazilian physician scientists coordinating their research in an unprecedented way through the Coalition COVID-19 Brazil that conducted a series of globally important trials of repurposed drugs including dexamethasone and HCQ. Financial constraints on research were a major problem, although various important internationally-funded trials also had sites in Brazil. The reputation in vaccine development of Fiocruz and Butantan Institute, and the country's known capacity to conduct high quality trials, meant Brazil hosted key vaccine trials and was able to secure technology transfer agreements with two of the leading COVID-19 vaccine developers. With its very strong record, Brazil's SUS undertook the vaccinations and a pioneering study calculated vaccination had averted

over 40,000 deaths in people aged 80+ in Brazil by May 2021. There were important multi-disciplinary collaborations and acceleration of the translation of basic research into new devices. Brazil, however, suffered from a very high COVID-19 death rate which was widely blamed on the failure of the President and his government to listen to scientific evidence on issues such as NPIs, and also by their promotion of unproven drugs such as HCQ - securing its approval by over-ruling existing procedures. Despite the challenges, Brazil benefitted from the long-term build-up of research capacity and could provide lessons for other middle-income countries - with adequate funding Brazil could benefit from building on the rapid moves towards greater coordination.

References

1. Siemieniuk RAC, Bartoszko JJ, Ge L, Zeraatkar D, Izcovich A, Kum E, et al. Drug treatments for covid-19: living systematic review and network meta-analysis. BMJ. 2020; doi: https://doi.org/10.1136/bmj.m2980
2. Norton A, Bucher A, Antonio E, Advani N, Grund H, Mburu S, et al. A living mapping review for COVID-19 funded research projects: nine-month update [version 4; peer review: 2 approved]. Wellcome Open Research 2021; doi: https://doi.org/10.12688/wellcomeopenres.16259.4.
3. Brasil. Ministério da Saúde. Conselho Nacional de Saúde. Comissão Nacional de Ética em Pesquisa. Boletim Ética em Pesquisa - Edição Especial Coronavírus (Covid-19). CONEP/CNS/MS. 2021,1:1-13 ED70.cdr (saude.gov.br) Accessed 8 June 2022.
4. Rosa MFF, da Silva EN, Pacheco C, Millett C, Gadelha CAG, Santos LMP. Direct from the COVID-19 crisis: research and innovation sparks in Brazil. Health Res Policy Sys. 2021; doi: https://doi.org/10.1186/s12961-020-00674-x.
5. Brasil. Ministério da Saúde. Conselho Nacional de Saúde. Comissão Nacional de Ética em Pesquisa. Boletim Ética em Pesquisa - Edição Especial Coronavírus (Covid-19). CONEP/CNS/MS. 2020,1:1-71. ED28.cdr (saude.gov.br) Accessed 8 June 2022.
6. Campo KN, Rodrigues ICP, Lopes ÉSN, Gabriel LP. Early public research funding response to COVID-19 pandemic in Brazil. Rev Soc Bras Med Trop. 2020;53:e20200522. DOI: 10.1590/0037-8682-0522-2020
7. Zimerman A, Lopes RD, D'Ávila A, Rohde LE, Zimerman L. COVID-19 in Brazil: the headlines should be about science. (Letter) The Lancet, 2020; doi: https://doi.org/10.1016/S0140-6736(20)32375-8.
8. Tomazini BM, Maia IS, Cavalcanti AB, Berwanger O, Rosa RG, Veiga, VC, et al. Effect of dexamethasone on days alive and ventilator-free in patients with moderate or severe acute respiratory distress syndrome and COVID-19: the CoDEX randomized clinical trial. JAMA. 2020; doi: 10.1001/jama.2020.17021.
9. The WHO Rapid Evidence Appraisal for COVID-19 Therapies (REACT) Working Group. Association between administration of systemic corticosteroids and mortality among critically ill patients with COVID-19. A meta-analysis. JAMA. 2020; doi: https://dx.doi.org/10.1001%2Fjama.2020.17023
10. Cavalcanti AB, Zampieri FG, Rosa RG, Azevedo LCP, Veiga VC, Avezum A, et al. Hydroxychloroquine with or without azithromycin in mild-to-moderate Covid-19. NEJM. 2020; doi: https://doi.org/10.1056/NEJMoa2019014.
11. Axfors C, Schmitt AM, Janiaud P, van't Hooft J, Abd-E-Salam S, Abdo EF, et al. Mortality outcomes with hydroxychloroquine and chloroquine in COVID-19 from an international collaborative meta-analysis of randomized trials. Nat Commun. 2021; doi: https://doi.org/10.1038/s41467-021-22446-z.
12. RECOVERY Collaborative Group. Effect of hydroxychloroquine in hospitalized patients with Covid-19. N. Engl. J. Med. 2020; doi: https://doi.org/10.1056/NEJMoa2022926.

13. WHO Solidarity Trial Consortium. Repurposed antiviral drugs for COVID-19; interim WHO Solidarity trial results. Preprint. 2020; doi: https://doi.org/10.1101/2020.10.15.20209817.
14. WHO Solidarity Trial Consortium. Repurposed antiviral drugs for Covid-19 — interim WHO Solidarity trial results. NEJM. 2021; doi: https://doi.org/10.1056/NEJMoa2023184.
15. Wise J, Coombes R. Covid-19: The inside story of the RECOVERY trial. BMJ. 2020; doi: https://doi.org/10.1136/bmj.m2670.
16. White NJ, Watson JA, Hoglund RM, Chan XHS, Cheah PY, Tarning J. COVID-19 prevention and treatment: a critical analysis of chloroquine and hydroxychloroquine clinical pharmacology. PLoS Med. 2020; doi: https://doi.org/10.1371/journal.pmed.1003252.
17. Furtado RHM, Berwanger O, Fonseca HA, Correa TD, Ferraz LR, Lapa MG, et al. Azithromycin in addition to standard of care versus standard of care alone in the treatment of patients admitted to the hospital with severe COVID-19 in Brazil (COALITION II): a randomised clinical trial. Lancet. 2020; doi: https://doi.org/10.1016/S0140-6736(20)31862-6.
18. Reis G, Silva EASM, Silva DCM, Thabane L, Milagres AC, Ferreira TS, et al. Effect of early treatment with fluvoxamine on risk of emergency care and hospitalisation among patients with COVID-19: the TOGETHER randomised, platform clinical trial. Lancet Glob Health. 2022; doi: https://doi.org/10.1016/S2214-109X(21)00448-4.
19. Schraer R, Goodman J. Ivermectin: How false science created a Covid 'miracle' drug. BBC, Health. Ivermectin: How false science created a Covid 'miracle' drug - BBC News Accessed 16 May 2022.
20. Reis G, Silva EASM, Silva DCM, Thabane L, Milagres AC, Ferreira TS, et al. Effect of early treatment with ivermectin among patients with Covid-19. NEJM 2022;386:1721-31. DOI: 10.1056/NEJMoa2115869
21. The ATTACC, ACTIV-4a, and REMAP-CAP Investigators. Therapeutic anticoagulation with heparin in noncritically ill patients with Covid-19. NEJM. 2021; doi: 10.1056/NEJMoa2105911.
22. Gupta A, Gonzalez-Rojas Y, Juarez E, Casal, MC, Moya, J, Falci, DR, et al. Early treatment for Covid-19 with SARS-CoV-2 neutralizing antibody sotrovimab. NEJM 2021; doi: 10.1056/NEJMoa2107934.
23. Jayk Bernal A, Gomes da Silva MM, Musungaie DB, Kovalchuk E, Gonzalez A, Delos Reyes D, et al. Molnupiravir for oral treatment of Covid-19 in nonhospitalized patients. NEJM. 2021; doi: 10.1056/NEJMoa2116044.
24. Pfizer. Pfizer announces additional phase 2/3 study results confirming robust efficacy of novel COVID-19 oral antiviral treatment candidate in reducing risk of hospitalization or feath. Pfizer, 14 Dec 2021. Pfizer Announces Additional Phase 2/3 Study Results Confirming Robust Efficacy of Novel COVID-19 Oral Antiviral Treatment Candidate in Reducing Risk of Hospitalization or Death | Pfizer. Accessed 24 Dec 2021.
25. NIH/US National Library of Medicine/ClinicalTrials.gov. Evaluation of protease inhibition for COVID-19 in standard-risk patients (EPIC-SR). Identifier: NCT05011513. Evaluation of Protease Inhibition for COVID-19 in Standard-Risk Patients (EPIC-SR). - Full Text View - ClinicalTrials.gov. Accessed 24 Dec 2021.
26. Gilbert S, Green C. Vaxxers. The inside story of the Oxford AstraZeneca vaccine and the race against the virus. London: Hodder and Stoughton; 2021.
27. Instituto Butantan. Projeto S: imunização em serrana faz casos de covid-19 despencarem 80% e mortes, 95%. https://butantan.gov.br/noticias/projeto-s-imunizacao-em-serrana-faz-casos-de-covid-19-despencarem-80-e-mortes-95. Accessed 3 Jan 2022.
28. BBC News. Brazil Covid: Deaths plunge after town's adults vaccinated. BBC. 1 June 2021. https://www.bbc.co.uk/news/world-latin-america-57309538 Accessed 13 Jan 2022.
29. Katikireddi SV, Cerqueira-Silva T, Vasileiou E, Robertson C, Amele S, Pan J, et al. Two-dose ChAdOx1 nCoV-19 vaccine protection against COVID-19 hospital admissions and deaths over time: a retrospective, population-based cohort study in Scotland and Brazil. Lancet. 2021; https://doi.org/10.1016/S0140-6736(21)02754-9.

30. Pan American Health Organisation (PAHO). PAHO selects centers in Argentina, Brazil to develop COVID-19 mRNA vaccines. PAHO, 21 Sept 21. PAHO selects centers in Argentina, Brazil to develop COVID-19 mRNA vaccines - PAHO/WHO | Pan American Health Organization. Accessed 28 Sept 2021.
31. FIOCRUZ. Fundação Oswaldo Cruz: uma instituição a serviço da vida. Vacina contra Covid-19. Vaccines against Covid-19 (portal-fiocruz-br.translate.goog) Accessed 19 July 2022.
32. Lancet Commission on COVID-19 Vaccines and Therapeutics Task Force Members. Operation Warp Speed: implications for global vaccine security. Lancet Glob Health. 2021; doi: https://doi.org/10.1016/S2214-109X(21)00140-6.
33. Victora PC, Castro PMC, Gurzenda S, Medeiros AC, Franca GVA, Barros AJD. Estimating the early impact of vaccination against COVID-19 on deaths among elderly people in Brazil: analyses of routinely-collected data on vaccine coverage and mortality. EClinicalMedicine. 2021; doi: 10.1016/j.eclinm.2021.101036.
34. FIOCRUZ. Institute of Communication and Scientific and Technological Information in Health (ICICT). Monitorcovid-19. Rio de Janeiro, 2020. Available at: https://bigdata-covid19.icict.fiocruz.br/. Accessed 13 Jan 2022.
35. da Silva RGL, Chammas R, Plonski A, Goldbaum M, Novaes HMD. University participation in the production of molecular diagnostic tests for the novel coronavirus in Brazil: the response to health challenges. Cad Saúde Pública. 2020; doi: https://doi.org/10.1590/0102-311X00115520.
36. Carpes Lanes T, Lima Ribeiro MA, Seoni de Oliveira D, Gabriel do Nascimento MG, Garcia FR, Franca de Oliveira Menco JM, et al. Clinical practice guidelines for the treatment of Covid-19 in Brazil: a systematic review. Rev. Cuid. 2021; doi: http://dx.doi.org/10.15649/cuidarte.2025.
37. Lamontagne F, Agoritsas T, Macdonald H, Leo YS, Diaz J, Agarwal A, et al. A living WHO guideline on drugs for covid-19. BMJ. 2020; doi: http://dx.doi.org/10.1136/bmj.m3379.
38. Pan American Health Organisation (PAHO). Brazilian Post launches stamps dedicated to COVID-19 in association with PAHO.8 July 2020. Brazilian Post launches stamps dedicated to COVID-19 in association with PAHO - PAHO/WHO | Pan American Health Organization. Accessed 1 Dec 2021.
39. Bruce, Cavgias A, Meloni L, Remígio M. Under pressure: women's leadership during the COVID-19 crisis. J Development Economics. 2022; doi: https://doi.org/10.1016/j.jdeveco.2021.102761 .
40. Rodríguez Mega E. Latin America's embrace of an unproven COVID treatment is hindering drug trials. Nature. 2020; 586:481-2. doi: https://doi.org/10.1038/d41586-020-02958-2
41. Florez ID, Amer YS, McCaul M, Lavis JN, Brouwers M. Guidelines developed under pressure. The case of the COVID-19 low-quality "rapid" guidelines and potential solutions. J Clin Epidemiol. 2021; doi: 10.1016/j.jclinepi.2021.11.012.
42. Souza D de O. Cloroquine e hidroxicloroquina no Brasil: um caso de ineficácia na gestão da saúde pública (Translated: Chloroquine and hydroxychloroquine in Brazil: a case of inefficacy in public health management). Rev Salud Pública. 2021;23(2):1-7. DOI: https://doi.org/10.15446/rsap.v23n2.89741
43. Nature Editorial. India, Brazil and the human cost of sidelining science. Nature. 2021;593:7-8.
44. Lancet Editorial. COVID-19 in Brazil: "So what?" Lancet. 2020;395:1461.
45. Riley C, Xie B, Khurshid A. Challenges encountered in comparing international policy responses to COVID-19 and their effects. Health Res Policy Sys. 2021; doi: https://doi.org/10.1186/s12961-021-00783-1.
46. Urbinatti A, Monteiro M, Di Giulio G. Brazil. In: Jasanoff S, Hilgartner SJ, Hurlbut JB, Ozgode O, Rayzberg M. Comparative Covid Response: Crisis, Knowledge, Politics. Interim Report. 12 Jan 2021. https://compcore.cornell.edu/wp-content/uploads/2021/03/Comparative-Covid-Response_Crisis-Knowledge-Politics_Interim-Report.pdf. Accessed 12 Jan 2022.

47. Taylor L. 'We are being ignored': Brazil's researchers blame anti-science government for devastating COVID surge. Nature. 2021; doi: https://doi.org/10.1038/d41586-021-01031-w.
48. BBC. Brazil senators back criminal charges against Bolsonaro over Covid handling. BBC News, 27 Nov 2021. Brazil senators back criminal charges against Bolsonaro over Covid handling - BBC News. Accessed 13 Jan 2022.
49. Global Health Security Index (2019). Nuclear Threat Initiative (NTI) and John Hopkins. Oct 2019. https://www.ghsindex.org/wp-content/uploads/2021/11/2019-Global-Health-Security-Index.pdf. Accessed 8 Dec 2021
50. Mineo L, Castro M. Why the COVID-19 outbreak in Brazil has become a humanitarian crisis. The Harvard Gazette, 28 April 2021. Harvard public health expert discusses Brazil's COVID-19 crisis – Harvard Gazette. Accessed 28 Dec 2021.
51. Global Health Security Index (On-going). About. About - GHS Index. Accessed 6 Nov 2021.
52. Rodrigues M. Scientists reel as Brazilian government backtracks on research funds. Nature News. 22 Oct 21. doi: https://doi.org/10.1038/d41586-021-02886-9.

Canada

Section 1: *Examples of achievements in production of primary and secondary research to inform development of COVID therapies, vaccines, national/local organisational responses; examples (and lessons) about links to existing strengths in the health research system*

Global analysis conducted by a team from the UK and Canada suggested Canada had the third highest number of funded COVID-19 projects [1]. On 23 April 2020 Prime Minister (PM) Trudeau announced more than CAD1 billion investment in *"A national medical research strategy to fight COVID-19"* covering various research areas and modes of support to build on an announcement on 23 March 2020 of CAD275m [2,3].

The additional funding included some for organisations funded in the first tranche, and the largest element was CAD600m for the Strategic Innovation Fund to support COVID-19 vaccine and therapy trials led by the private sector, and Canadian biomanufacturing opportunities. The many other areas included an additional CAD115m for CIHR projects to accelerate development and implementation of medical and social countermeasures against COVID-19, and the establishment of the COVID-19 Immunity Task Force to develop, coordinate and implement various immunity studies to provide information to decision-makers. There was also CAD10m for the long-established Canadian Immunization Research Network to study COVID-19 immunity, conduct vaccine-related research and clinical trials *"and to enhance Canada's capacity to monitor vaccine safety and effectiveness"* [2,4]. In terms of COVID-19 research publications, Canada had the 7th highest number, with over 60% being multinational collaborations [5].

At the federal level, in April 2020, Innovation, Science and Economic Development Canada with the support of the Chief Science Advisor, Mona Nemer, created CanCOVID as a Canada-wide network of health, science and policy researchers to facilitate COVID-19 research collaboration [6]. (But see section 3.)

The first call from the Canadian Institutes of Health Research (CIHR) on 10 February 2020, made in combination with other Canadian research funding councils, requested rapid COVID-19 proposals by 18 February [7], with the peer review and initial decisions made rapidly [8]. While the original call included the study of countermeasures *"tailored to the unique circumstances of different populations"* as one of the relevant areas that could be funded, some later calls were more targeted, and became more focused as gaps in research were identified in areas such as Indigenous communities' experience with COVID-19 and keeping residents and staff of long-term care homes safe [7]. CIHR's first specific Indigenous COVID-19 rapid research programme was launched in September 2020 [9]. The Canadian Treatments for COVID-19 trial had already been set-up to work with WHO's Solidarity trial, itself informed by WHO's Global Research Collaboration for Infectious Disease Preparedness [7,8,10]. A CIHR targeted call requested proposals to enable it to expand with further Canadian sites. A clinician linked to the University of British Columbia (UBC) led the successful proposal and was on the trial's writing committee [7,10-12].

Through its existing Strategy for Patient-Oriented Research (SPOR) Innovative Trials Programme, the CIHR also funded Canadian research that reported key findings as part of the REMAP-CAP platform

trials of corticosteroids and IL-6 receptor antagonist [13,14]. (See next section for Canadian-led WHO guidelines using these studies.)

Canadian sites played a key role in other international trials, as noted above in the Brazilian section. The ATTACC platform trial was led by Canadian sites and had a consortium of Canadian funders including CIHR, Ontario Ministry of Health, the Thistledown Foundation (through FastGrants from the US), the Peter Munk Cardiac Centre in Toronto, and Research Manitoba. Additional funding came from the LifeArc Foundation from the UK. Despite the Canadian leadership, the ATTACC platform trial enrolled more patients in the US and Brazil. Jointly with the two other platforms (including REMAP-CAP) its findings published in *NEJM* showed in noncritically ill COVID-19 patients an initial strategy of therapeutic-dose anticoagulation with heparin increased probability of survival to hospital discharge [15]. In the three platforms' parallel trial of the treatment with critically ill patients, REMAP-CAP took the lead and most patients were enrolled in the UK, but the treatment was not successful [16]. The TOGETHER platform trial was co-led by McMaster University (at Hamilton, Ontario) but conducted in Brazil. Its findings suggested fluvoxamine as an early treatment reduced the chance of hospitalisation [17]. Reliable reports in Autumn 2021 that another arm of TOGETHER would show no benefit from the use of ivermectin [18], were confirmed in the trial's report in the *NEJM* in 2022 which attracted considerable attention [19].

Other completed trials included one testing the mAb antibody sotrovimab, which found it an effective early treatment for COVID-19 [20]. The *NEJM* paper's first author was from a Canadian medical centre in Toronto, but most sites were in the US.

CIHR's targeted call for a project to generate knowledge synthesis and other evidence products targeting COVID-19 related topics [21] was an area of internationally-recognised Canadian leadership. This led to funding of CAD1m to support COVID-END at McMaster University, which leveraged the existence of the CIHR-funded SPOR Evidence Alliance (see section 2) [22]. COVID-END, *"a time-limited network that brings together more than 50 of the world's leading evidence-synthesis, technology-assessment and guideline-development groups around the world"* aimed to help the production and use of evidence syntheses [22]. (See next section for the key role of reviews in informing Canadian policy.) Researchers associated with COVID-END, and others, were concerned about the low quality of some rapidly produced guidelines across the globe, in contrast to the high quality living guidelines. Therefore, they undertook a living review of rapid COVID-19 guidelines [23]. This found the quality had often remained low, but they identified potential solutions.

Various initiatives at provincial and hospital levels aimed to bring together the needs and priorities of provincial governments, health systems and hospitals with local research capacity and draw on federal, provincial and hospital research funding. Ontario was one of many places where teams conducted wastewater surveillance, including to detect outbreaks in 'hotspot' communities. A review performed by Public Health Ontario showed how such studies had become widespread and were being used in interdisciplinary research [24]. The modellers in the Ontario team also estimated surges in cases and intensive care admission using funding from federal (CIHR), provincial and institutional (St. Michael's) levels [25].

In Alberta, the projects combining state funding with that from CIHR included one from the University of Alberta where several teams collaborated on early stage progress developing possible new antiviral treatments for COVID-19 [26,27]. In Quebec, the Government created an

interdisciplinary network to examine ways to better coordinate research efforts, including clinical research networks [28,29]. Federal and provincial funding contributed to developing vaccine candidates in research centres and companies in various provinces [30], with some recipients named in the PM's first funding statement of March 2020 [3]. One such major example was the work of the University of Saskatchewan's Vaccine and Infectious Disease Organization-International Vaccine Centre which had previously produced two coronavirus vaccines for animals. Another was Medicago, a company in Quebec City that was using a plant-based vaccine technology it had been working on for 20 years and that had originally been developed by Agri-Food Canada and Laval University. It received federal funding for animal and human testing of its COVID-19 vaccine as well as expansion of its manufacturing capacity, and also CAD7m from the Quebec government. It was the first Canadian vaccine candidate to start clinical trials in July 2020 [30].

The provincial level – British Columbia (BC)

In BC, examples of research infrastructure and partnerships existing prior to the pandemic were described in the background section of the main paper. COVID-19 research funding leveraged these to facilitate the creation of new coordination structures such as the BC COVID-19 Strategic Research Advisory Committee and its working groups, including vaccine effectiveness, long-term care and public communications. This brought together the needs of the leaders of the provincial government and health system with the health research community [31].

An evaluation emphasised that no one organisation had formal responsibility for implementing the BC research response, rather there was a collegial effort to collaborate, with David Patrick and the BC Centre for Disease Control [32], where he was Director of Research, widely seen as playing a key role in coordination [31]. Nevertheless, the evaluation also highlighted the importance of the leadership provided by the Provincial Health Officer, Bonnie Henry, in directing the requirement for research coordination, and Michael Smith Health Research BC *"through its long-standing and respected role as a broker between the research community and decision-makers"* [31]. Health Research BC launched a COVID-19 research competition in line with the strategic priorities identified in the province, as well as four research projects on urgent priorities requested by the Provincial Health Officer. Another key factor was that through the federal funding described above, and the provincial funding, *"The rapid and substantial infusion of research funding to established institutions and researchers was perceived by many stakeholders as the most important contributor to success of the research response"* [31]. The combination of the greater coordination and the collegial effort, creation of the BC Clinical Research Coordination Initiative, the availability of finance and the ability to leverage existing infrastructure, and some acceleration of ethics review and data access protocols, resulted in some successes (see next section). However, some researchers still experienced challenges (see section 3) [31].

Research conducted in BC prior to the pandemic proved to be of critical importance to the international research effort during the pandemic. Illustrating basic research's long-term impact, during the pandemic international attention focused on basic lipid nanoparticle technology research conducted years earlier by researchers including Pieter Cullis at UBC in Vancouver, BC, and his spin-off companies. Such work became crucial in the development of mRNA vaccines by providing a way of delivering the mRNA to cells [33,34].

The first tranche of science funding announced by the federal PM included support related to mAb development work by AbCellera Biologics, another Vancouver-based spin-off company from UBC [3]. AbCellera's technology meant that since 2018 it had been funded by the Pandemic Prevention Platform of the US Defense Advanced Research Projects Agency (DARPA) *"to establish a robust technology platform for pandemic response capable of developing field-ready medical countermeasures within 60 days of isolation of an unknown viral pathogen"* [35]. This meant it was ready to go in January 2020 and discovered bamlanivimab which was developed with its partner Eli Lilly as the first mAb therapy to reach human testing and to get FDA approval. This further development and, especially, trialling of the mAb therapy was increasingly undertaken by Eli Lilly in the US [36,37]; further successes and challenges are discussed in the US section below. (However, here we note that in January 2022, the US Food and Drugs Agency (FDA) withdrew its Emergency Use Authorisation for the mAb, but then on 11 February, the FDA gave Emergency Use Authorisation to a new mAb, bebtelovimab, that was effective against Omicron and that again AbCellera had discovered and Eli Lilly developed [38].)

Section 2: *Examples of achievements in promoting evidence use in products, policymaking and practice; examples (and lessons) about links to existing strengths*

It is claimed that compared to many countries, Canada holds evidence in high regard [39] and in making the announcement of the CAD1 billion investment in medical research (see section 1), PM Trudeau said *"We are making sure that Canada remains at the forefront of scientific research to help us make smart and effective decisions on the path to recovery"* [2]. That announcement built on the PM's previous one entitled: *"Canada's plan to mobilize science to fight COVID-19*" [3].

The full data from which Table 1 in the main paper was drawn show the deaths in Canada were considerably lower not only than in its neighbour, the US, as a whole, but also lower than in 48 of the 50 states of the US, and just marginally higher than in the remaining two states [40]. The excess deaths figures from Table 1 also suggest Canada did even better compared to the US [41]. One of the reasons cited in 2020 for the much lower death rate in Canada was a greater willingness to listen to the evidence than existed in the US under President Trump: *"Though Canada's response has not been entirely devoid of politics, Canadian officials have consistently deferred to public health experts and scientists to drive policy decisions"* [42].

An analysis of why Canada's COVID death rate was so much lower than in the US the first two years of the pandemic also identified the role of scientists and public health experts in encouraging politicians to support and retain firmer NPIs than were generally applied in the US [43]. But there were also other factors. These included the differences in the healthcare systems that existed at the start of the pandemic, with Canada having a universal publicly funded system that was lacking in the US. Another difference was the vaccination rate, where Canada rapidly overtook the US in the summer of 2021 [43]. By mid-August 2021, it was claimed that Canada had achieved *"the highest vaccination rate - of single and double doses – anywhere in the world [through] a combination of savvy negotiations, financial resources and high trust in public health institutions"* [44]. As Table 1 showed, at the end of 2021 Canada was still maintaining the highest vaccination rate across our countries.

Prior to the pandemic, the SPOR Evidence Alliance, a partnership of researchers and health system stakeholders, had been created to work *"towards creating a collaborative research environment that is centred around patients and health system decision-makers"* [45]. During the pandemic it conducted workshops on producing rapid reviews for Health Canada as well as the Public Health Agency of Canada. Patients and researchers co-created and led workshops on patient engagement in reviews to illustrate how engagement could happen, even in rapid reviews done on short timelines for policy makers. In addition to COVID END and the SPOR Evidence Alliance, CanCOVID also began conducting knowledge synthesis for policymakers with its mission *"to enable the agile, evidence-based decision-making needed to help steer Canada safely through the COVID-19 pandemic*" [6].

Informed by RECAP-MAP and other trials (see section 1), various versions of WHO's living COVID guidelines first recommended dexamethasone, and later added IL-6 receptor antagonist, with the respective lead authors being from Canada [46,47]. As noted, successful work to turn AbCellera's discovery of bamlanivimab into an mAb therapy involved various trials to decide exactly for which patients, and in which combinations, it should be used, but its use for non-hospitalised patients received emergency authorisation from Health Canada as early as November 2020 [37,48].

The National Advisory Committee on Immunization provided advice to the Government on aspects of COVID-19 vaccines, and also published guidance, statements and many updates [49].

As one of various initiatives to combat misinformation about COVID-19, a collective of independent scientists, healthcare experts and science communicators supported an idea suggested by Timothy Caulfield from the University of Alberta, and created *ScienceUpFirst* [50]. The movement encouraged supporters to share expert-vetted posts countering COVID-19 misinformation on social media.

Canada (along with Germany and New Zealand) was included in an analysis, conducted by UBC and published in September 2020, of communications in nine countries selected partly because they *"managed relatively effective responses"* to COVID-19 [51]. The study found that communications was an effective NPI against COVID-19. Some themes were common across the jurisdictions: *"Despite the differences, many of our case studies offered similar best practices: clear, evidence-based messaging; materials translated into multiple languages.... compassionate, empathetic acknowledgement of the difficulties of Covid-19 response"* [51].

At the federal level, Canadian public health communications were seen as *"clear and understandable, emphasizing science and expertise; an innovation team embedded within the federal government incorporated insights from behavioural science to shape Covid-19 messaging"* [52]. However, an analysis of the first full year of the pandemic took a rather different angle and claimed that *"limited national and interregional coordination of public health communication was apparent throughout the pandemic, and the federal government fell short in leveraging its unique position to unite the public in supporting measures that help mitigate the pandemic"* [53]. This critical analysis did, however, praise the COVID-19 communications in BC led by Bonnie Henry during the first year.

Furthermore, the 2020 cross-national report from UBC looked in detail at the COVID-19 communications in two Canadian provinces - BC and Ontario - with the former viewed as having adopted a much more effective approach than the latter. A specific part of the analysis identified both BC and Sweden as having more effective communications of the jurisdictions' respective

policies to combat the pandemic than Ontario, but because Ontario's policies were broadly more effective than Sweden's, it joined BC in having a lower death rate than Sweden [51].

The analysis specifically about BC showed how the communications approach seemed to mirror several of the important generic points listed above with epidemiological information being accompanied by regular and extensive references to social or civic values. Furthermore, as noted, it was the non-elected Provincial Health Officer, Henry, who successfully took the lead in presenting this combined message in a way that reduced the opportunities for confusion or contradiction [54].

Similarly at the provincial level, there was interest across Canada in evidence for decision making; many provinces initiated science tables or committees that were available to inform policymakers e.g., the Ontario COVID-19 Science Advisory Table [55]. Other studies conducted with an explicit aim of integrating a knowledge translation approach included one from St Michael's Hospital, Toronto, Ontario, for long term care homes which sought to "*integrate immunity study results to tailor delivery to improve COVID-19 preparedness and outbreak management*" [56].

In BC, the strong network of regional plus provincial health authorities, the BC Centre for Disease Control and the connection to the provincial government facilitated evidence-informed decision making. As described in the evaluation of the response of the BC HRS, the BC COVID-19 Strategic Research Advisory Committee worked to connect research results with the health system, with the coordination being *"valued by senior decision-makers"* in the health ministry and health service [31]. Health system leaders perceived that when an issue arose they could receive information from the research system to help address the question [31].

Section 3: *Examples of challenges in: 1. production of evidence; 2. use of evidence; and 3. sustaining the research system; examples of lessons to address challenges*

Challenges in production of evidence

It was difficult to achieve a national prioritisation of research gaps that brought together the sub-national units and that consistently utilised the existing research entities. The result was duplication of effort across provinces, territories and at the national level, including the conduct of multiple knowledge syntheses and guidelines on the same topics across provinces, territories and federal policymakers, and limited public involvement in COVID-19 research [39].

CIHR drew on some analysis from early in the pandemic [8] to state: "*At this stage of the pandemic, the gap in Canada's clinical trials coordination infrastructure has once again been noted as an area in need of improvement*" [57]. While the role of some individual trialists and groups was noted, *"collaboration and coordination mechanisms across these groups have not been specifically funded"* [57]. CIHR, therefore, launched a call for proposals for greater coordination of research capacity and activities, possibly through a *"network of networks"* [57].

Similarly, based on analysis up to late 2020, François Lamontagne and colleagues claimed that despite Canada's large investment in health research during the pandemic, *"Our very limited success in contributing to the worldwide effort to find effective treatments for COVID-19, and discredit useless and harmful ones, have highlighted a broken system"* [28]. The team also identified three main problems, and contrasted the experience in Canada with the comparative success of the UK

where the NIHR was embedded into the healthcare system. This, they said, "*simultaneously solved problems related to infrastructure development, health system engagement and fragmentation in the UK context*" [28]. They claimed a culture change was needed in Canadian clinical research, along with the will to forge a partnership among the various health systems and research institutes and organisations. They suggested it might be useful to start with some pilot studies in some provinces [28] - see section 1 for some examples of progress at the provincial level.

There were calls as early as June 2020 for the early modelling that *"by necessity, assumed relative homogeneity in risks of infection and outcomes"* to be replaced by an approach *"Leveraging data on heterogeneity to guide nuanced, population-and setting-specific strategies"* [58]. Moreover, while COVID-19 research showed evidence of health disparities, research to preferentially address these disparities was slow to be launched.

Even in BC, with some successes in coordinating the provincial health research system, aspects of the challenges for research coordination appeared to relate to long-standing problems facing the HRS. These included limitations in structures to support research within the health system [31], lack of clear authority for the coordination, and related challenges with aspects of research priority-setting. Despite the rapid ethical approval for the Canadian Treatments for COVID-19 trial in BC [8], some BC researchers still faced delays in securing ethical approval and access to data [31]. This limited their ability to contribute timely research as they had hoped.

Challenges in use of evidence

As in other jurisdictions, challenges existed in using evidence in policy decisions. While evidence existed to inform decisions, it was not always used by policy makers who also balanced political and economic interests. Moreover, clear messaging to the public on the evolving nature of the evidence given rapid research production was not consistently used.

While agreeing that Canada had controlled the virus more successfully than the US, some analysts pointed to countries such as Australia and New Zealand as having a much lower death rate than Canada. One such article acknowledged the federal government's crucial role in funding COVID-19 scientific research, but was critical of some other aspects of the Canadian response where the federal nature of the country did not seem to work so well, especially after the first wave, including failures on information sharing and *"the inability to maintain an adequate public health surveillance system"* to support local decision making [53]. That article also highlighted the much higher proportion of deaths that were in the long-term care sector in Canada compared to other high income countries. But, it noted that the death rate in care homes in BC was lower than the average across Canada, something that Liu et al., thought was partly caused by the consistent communication about the pandemic from the Provincial Health Officer and elected leaders in BC – see above [59].

Despite the general readiness to draw on research evidence, and a lower death rate than in many comparable countries, an equity lens has not been consistently used in creating science-informed policies during the pandemic [58].

COVID-19 deaths/cases per million population varied greatly between provinces [53,60], which might be partly related to different approaches to policymaking and use of evidence, including in

communications. Various complex Intellectual Property issues and disputes arose, including around the lipid technology [33,34].

Challenges in sustaining the research system

Challenges faced by the HRS during the pandemic included reduced funding in some areas (e.g., in 2020 CIHR delayed one of their project grant competitions, thereby impacting hundreds of researchers [61]) and reduced career opportunities, especially for women [39,62]. Examples of attempts to address these pressures occurred at federal level with CIHR taking steps to implement gender policy changes [63], and provincial level, for example in BC [62]. There was also reduced use of effective Patient and Public Involvement (PPI) during the pandemic, including in BC notwithstanding the increased coordination [31].

Summary

While Canada experienced fewer COVID-related deaths than the US, it experienced research challenges including duplication of effort and the need to create infrastructure to support trials platforms. Canada responded relatively quickly to the need for creation of these platforms and the challenge will be to sustain these platforms post-pandemic. Earlier research in BC was crucial for the development of the mRNA vaccines in the US and Germany, and Canada made progress in developing its own vaccine through the work of long-established capacity, but more slowly than in some other countries. Furthermore, transdisciplinary research collaborations across multiple contexts and sectors facilitated a robust response to the pandemic and opportunities to sustain these relationships will be critical to enabling future research advances. The pandemic further highlighted the need to preferentially conduct research to address health disparities in Canada. Key successes in Canada included leveraging existing systems for conducting knowledge synthesis and engaging patients in the conduct of rapid knowledge synthesis. Similarly, strong relationships between researchers and research users (including public health and policy makers) are fundamental to successful pandemic response and examples of this in BC illustrated success.

References

1. Norton A, Bucher A, Antonio E, Advani N, Grund H, Mburu S, et al. A living mapping review for COVID-19 funded research projects: nine-month update [version 4; peer review: 2 approved]. Wellcome Open Research 2021; doi: https://doi.org/10.12688/wellcomeopenres.16259.4.
2. Prime Minister of Canada. Prime Minister announces new support for COVID-19 medical research and vaccine development. Press release, Ottawa, Canada, 23 April 2020. Prime Minister announces new support for COVID-19 medical research and vaccine development | Prime Minister of Canada. Accessed 13 Jan 2022.
3. Prime Minister of Canada. Canada's plan to mobilize science to fight COVID-19. Press release, Ottawa, Canada, 23 March 2020. https://pm.gc.ca/en/news/news-releases/2020/03/23/canadas-plan-mobilize-science-fight-covid-19. Accessed 29 June 2022.
4. Canadian Immunization Research Network. About Us. About Us | CIRN (cirnetwork.ca). Accessed 17 Nov 2021.
5. Kim K, Cho KT. A review of global collaboration on COVID-19 research during the pandemic in 2020. Sustainability, 2021; doi: https://doi.org/10.3390/su13147618.
6. Office of the Chief Science Advisor, Government of Canada. CanCOVID Network. 2020. https://www.ic.gc.ca/eic/site/063.nsf/eng/h_98020.html. Accessed 8 Nov 2021.

7. Canadian Institutes of Health Research (CIHR). CIHR funding opportunities. CIHR Funding opportunities - CIHR (cihr-irsc.gc.ca). Accessed 14 Jan 2022.
8. Cheng MP, Lee TC, Tan DHS, Murthy S. Generating randomized trial evidence to optimize treatment in the COVID-19 pandemic. CMAJ. 2020; doi: https://doi.org/10.1503/cmaj.200438.
9. CIHR. Indigenous COVID-19 Rapid Research Funding Opportunity. CIHR. 29 Sept 2020 https://www.researchnet-recherchenet.ca/rnr16/vwOpprtntyDtls.do?all=1&masterList=true&org=CIHR&prog=3378&resultCount=25&sort=program&type=EXACT&view=currentOpps&language=E Accessed 19 July 2022.
10. ClinicalTrials.gov. Treatments for COVID-19: Canadian Arm of the SOLIDARITY Trial (CATCO). NIH/US National Library of Medicine/ClinicalTrials.gov. NCT04330690. Treatments for COVID-19: Canadian Arm of the SOLIDARITY Trial - Full Text View - ClinicalTrials.gov Accessed 15 Jan 2022.
11. WHO Solidarity Trial Consortium. Repurposed antiviral drugs for COVID-19; interim WHO Solidarity trial results. Preprint. 2020; doi: https://doi.org/10.1101/2020.10.15.20209817.
12. WHO Solidarity Trial Consortium. Repurposed antiviral drugs for Covid-19 — interim WHO Solidarity trial results. NEJM. 2021; doi: https://doi.org/10.1056/NEJMoa2023184.
13. The Writing Committee for the REMAP-CAP Investigators. Effect of hydrocortisone on mortality and organ support in patients with severe COVID-19: The REMAP-CAP COVID-19 corticosteroid domain randomized clinical trial. JAMA. 2020; doi:10.1001/jama.2020.17022.
14. The REMAP-CAP Investigators. Interleukin-6 receptor antagonists in critically ill patients with Covid-19. N. Engl. J. Med. 2021;384:1491-1502.
15. The ATTACC, ACTIV-4a, and REMAP-CAP Investigators. Therapeutic anticoagulation with heparin in noncritically ill patients with Covid-19. NEJM. 2021; doi: 10.1056/NEJMoa2105911.
16. The REMAP-CAP, ACTIV-4a, and ATTACC Investigators. Therapeutic anticoagulation with Heparin in critically ill patients with Covid-19. NEJM. 2021; doi: 10.1056/NEJMoa2103417.
17. Reis G, Silva EASM, Silva DCM, Thabane L, Milagres AC, Ferreira TS, et al. Effect of early treatment with fluvoxamine on risk of emergency care and hospitalisation among patients with COVID-19: the TOGETHER randomised, platform clinical trial. Lancet Glob Health. 2022; doi: https://doi.org/10.1016/S2214-109X(21)00448-4.
18. Schraer R, Goodman J. Ivermectin: How false science created a Covid 'miracle' drug. BBC, Health. Ivermectin: How false science created a Covid 'miracle' drug - BBC News Accessed 16 may 2022.
19. Reis G, Silva EASM, Silva DCM, Thabane L, Milagres AC, Ferreira TS, et al. Effect of early treatment with ivermectin among patients with Covid-19. NEJM 2022;386:1721-31. DOI: 10.1056/NEJMoa2115869
20. Gupta A, Gonzalez-Rojas Y, Juarez E, Casal, MC, Moya, J, Falci, DR, et al. Early treatment for Covid-19 with SARS-CoV-2 neutralizing antibody sotrovimab. NEJM 2021; doi: 10.1056/NEJMoa2107934.
21. CIHR. COVID-19 Knowledge Synthesis Network. CIHR. 29 Oct 2020. https://www.researchnet-recherchenet.ca/rnr16/vwOpprtntyDtls.do?prog=3386&view=currentOpps&type=EXACT&resultCount=25&sort=program&next=1&all=1&masterList=true Accessed 10 March 2022. COVID-END.
22. COVID-END. COVID-19 evidence network to support decision-making. COVID-END (mcmasterforum.org). Accessed 8 Nov 2021.
23. Florez ID, Amer YS, McCaul M, Lavis JN, Brouwers M. Guidelines developed under pressure. The case of the COVID-19 low-quality "rapid" guidelines and potential solutions. J Clin Epidemiol. 2021; doi: 10.1016/j.jclinepi.2021.11.012.
24. Public Health Ontario. Wastewater Surveillance of COVID-19. Public Health Ontario. April, 2021. Wastewater Surveillance of COVID-19 (publichealthontario.ca) Accessed 15 March 2022.

25. Mishra S, Wang L, Huiting M, Yiu CKY, Peterson JM, Kim E, et al. Estimated surge in hospital and intensive care admission because of the coronavirus disease 2019 pandemic in the Greater Toronto Area, Canada: a mathematical modelling study. CMAJOpen. 2020; doi: 10.9778/cmajo.20200093.
26. Rutherford G. U of A team uncovers new compounds to slow SARS-CoV-2 replication mechanism. University of Alberta press release, 21 Oct 2021. U of A team uncovers new compounds to slow SARS-CoV-2 replication mechanism | Folio (ualberta.ca). Accessed 9 Aug 2022.
27. Vuong W, Khan MB, Fischer C, Arutyunova E, Lamer T, Shields J, et al. Feline coronavirus drug inhibits the main protease of SARS-CoV-2 and blocks virus replication. Nat Commun. 2020; doi: https://doi.org/10.1038/s41467-020-18096-2.
28. Lamontagne F, Rowan KM, Guyatt G. Integrating research into clinical practice: challenges and solutions for Canada. CMAJ 2021; doi: https://doi.org/10.1503/cmaj.202397.
29. Quebec COVID — Pandemic Network (RQCP). Quebec: Fonds de recherche du Quebec – Santé; 2020. https://rqcp.ca/groupes-de-travail/#recherche-clinique. Accessed 15 Jan 2022.
30. Chung E. A closer look at Canada's homegrown COVID-19 vaccine candidates. CBC News. 19 Oct 2020. A closer look at Canada's homegrown COVID-19 vaccine candidates | CBC News Accessed 9 June 2022.
31. Penny Cooper & Associates. Rapid review of the coordination of the provincial covid-19 research response. https://www.msfhr.org/wp-content/uploads/2021/10/BC-Covid19-Research-Response-Coordination_Rapid-Review-Report-20210521final.pdf. Accessed 27 Nov 2021.
32. British Columbia Centre for Disease Control. Our Research. BCCDC. Our Research (bccdc.ca). Accessed, 16 Dec 2021.
33. Dolgin E. The tangled history of mRNA vaccines. Nature. 2021; doi: https://doi.org/10.1038/d41586-021-02483-w.
34. Gaviria M, Kilic B. A network analysis of COVID-19 mRNA vaccine patents. Nature Biotechnology. 2021; doi: https://doi.org/10.1038/s41587-021-00912-9.
35. AbCellera. AbCellera is mobilizing a response to the novel coronavirus outbreak as part of its DARPA P3 program. AbCellera press release, 28 Jan 2020. AbCellera is mobilizing a response to the novel coronavirus outbreak as part of its DARPA P3 program. Accessed 16 Jan 2022.
36. AbCellera. Emergency use authorization of AbCellera-ciscovered Bamlanivimab administered with Etesevimab expanded by the FDA to include post-exposure prophylaxis for the prevention of COVID-19. AbCellera press release, 16 Sept 2021. Emergency Use Authorization of AbCellera-Discovered Bamlanivimab Administered with Etesevimab Expanded by the FDA to Include Post-Exposure Prophylaxis for the Prevention of COVID-19. Accessed 16 Jan 2022.
37. Dougan M, Nirula A, Azizad M, Mocherla B, Gottleib RL, Chen P, et al. Bamlanivimab plus etesevimab in mild or moderate Covid-19. NEJM 2021; doi: 10.1056/NEJMoa2102685.
38. AbCellera. AbCellera-Discovered Antibody, Bebtelovimab, Receives U.S. FDA Emergency Use Authorization for the Treatment of Mild-to-Moderate COVID-19. AbCellera. 11 Feb 2022. AbCellera-Discovered Antibody, Bebtelovimab, Receives U.S. FDA Emergency Use Authorization for the Treatment of Mild-to-Moderate COVID-19 Accessed 13 Feb 2022.
39. Holmes B, Straus S. COVID-19 is testing the ability of Canada's health research system. The Globe and Mail/Michael Smith Foundation for Health Research. 26 Aug 2021. COVID-19 is testing the ability of Canada's health research system – Michael Smith Foundation for Health Research (MSFHR). Accessed 9 Nov 2021.
40. Worldometer: COVID live update: 236,408,442 cases and 4,826,759 deaths from the Coronavirus. COVID Live Update: 236,408,442 Cases and 4,826,759 Deaths from the Coronavirus - Worldometer (worldometers.info). Accessed: 1 Jan 2022, for data on 31 Dec 2021.

41. Giattino C, Ritchie H, Roser M, Ortiz-Ospina E, Hasell J. Excess mortality during the Coronavirus pandemic (COVID-19) – [data for end of 2021]. Excess mortality during the Coronavirus pan/demic (COVID-19) - Statistics and Research - Our World in Data. Accessed 10 Aug 2022.
42. Coletta A. Canada's coronavirus performance hasn't been perfect. But it's done far better than the U.S. Washington Post, 15 July 2020. https://www.washingtonpost.com/world/the_americas/coronavirus-canada-united-states/2020/07/14/0686330a-c14c-11ea-b4f6-cb39cd8940fb_story.html. Accessed 15 Jan 2022.
43. Debusmann B. Why is Canada's Covid death rate so much lower than US? BBC News. 15 February 2022. https://www.bbc.co.uk/news/world-us-canada-60380317 Accessed 16 Feb 2022.
44. Cecco L. 'I knew supply was coming': how Canada's push for Covid vaccines paid off. Guardian. 13 Aug 2021. https://www.theguardian.com/world/2021/aug/13/canada-anita-anand-covid-vaccine Accessed 5 June 2022.
45. Strategy for Patient-Oriented Research (SPOR) Evidence Alliance. Welcome to our Alliance. SPOR Evidence Alliance. Accessed 20 Dec 2021.
46. Lamontagne F, Agoritsas T, Macdonald H, Leo YS, Diaz J, Agarwal A, et al . A living WHO guideline on drugs for covid-19. BMJ. 2020; doi: http://dx.doi.org/10.1136/bmj.m3379.
47. Agarwal A, Rochwerg B, Lamontagne F, Siemieniuk RAC, Agoritsas T, Askie L, et al. A living WHO guideline on drugs for covid-19. BMJ.2020; doi: http://dx.doi.org/10.1136/bmj.m3379.
48. AbCellera. AbCellera-Discovered Antibody Granted Interim Authorization by Health Canada as a Treatment for COVID-19. AbCellera. 20 Nov 2020. AbCellera-Discovered Antibody Granted Interim Authorization by Health Canada as a Treatment for COVID-19 Accessed 13 Feb 2022.
49. National Advisory Committee on Immunization. National Advisory Committee on Immunization (NACI): statements and publications. Health Canada. Health science, research and data - Canada.ca. Accessed 16 Dec 2021.
50. ScienceUpFirst. Who We Are. Who Are SUF: Organizations and People - ScienceUpFirst. Accessed 15 Dec 2021.
51. Tworek H, Beacock I, Ojo E. Democratic Health Communications during Covid-19: A RAPID Response. Vancouver: UBC Centre for the Study of Democratic Institutions, September 2020. Democratic-Health-Communication-during-Covid_FINAL.pdf (ubc.ca) Accessed 4 May 2022.
52. Beacock I. Canada. In: Tworek H, Beacock I, Ojo E. Democratic Health Communications during Covid-19: A RAPID Response. Vancouver: UBC Centre for the Study of Democratic Institutions, September 2020; 55-58. Democratic-Health-Communication-during-Covid_FINAL.pdf (ubc.ca) Accessed 4 May 2022.
53. Allin S, Fitzpatrick T, Marchildon GP, Quesnel-Vallée A. The federal government and Canada's COVID-19 responses: from 'we're ready, we're prepared' to 'fires are burning'. Health Econ Policy Law. 2022 Jan;17(1):76-94 (Epub 22 June 2021). doi: 10.1017/S1744133121000220.
54. Beacock I. British Columbia. In: Tworek H, Beacock I, Ojo E. Democratic Health Communications during Covid-19: A RAPID Response. Vancouver: UBC Centre for the Study of Democratic Institutions, September 2020; 59-62. Democratic-Health-Communication-during-Covid_FINAL.pdf (ubc.ca) Accessed 4 May 2022.
55. Ontario COVID-19 Science Advisory Table. Science in service. Informing Ontario's response to COVID-19. Ontario COVID-19 Science Advisory Table (covid19-sciencetable.ca). Accessed 8 Nov 2021.
56. Wellness Hub. Wellness Hub: Understanding COVID-19 Transmission though Implementing and Evaluating an Intervention to Support Wellness, IPAC, Vaccine Uptake, and other Wraparound

Care Needs in LTCHs and Shelters. Protocol. May, 2021. https://osf.io/wqrst Accessed 16 March 2022.
57. CIHR. Operating Grant: Network of clinical trials networks: collaboration to evaluate COVID-19 and intervention strategies. https://www.researchnet-recherchenet.ca/rnr16/vwOpprtntyDtls.do?progCd=11093&language=E&org=CIHR. Accessed 15 Jan 2022.
58. Mishra S, Kwong JC, Chan AK, Baral SD. Understanding heterogeneity to inform the public health response to COVID-19 in Canada. CMAJ 2020;192:E684–5. DOI: https://doi.org/10.1503/cmaj.201112
59. Liu M, Maxwell CJ, Armstrong P, Schwandt M, Moser A, McGregor MJ, et al. COVID-19 in long-term care homes in Ontario and British Columbia. CMAJ. 2020 Nov 23;192(47):E1540-E1546. doi: 10.1503/cmaj.201860 Epub 2020 Sep 30.
60. Little N. COVID-19 Tracker Canada. 2000. COVID-19 Tracker Canada (covid19tracker.ca). Accessed 15 Jan 2022.
61. CIHR. Delay of strategic funding opportunities. June 23, 2020. Delay of strategic funding opportunities - CIHR (cihr-irsc.gc.ca). Accessed 17 Nov 2021.
62. Tiesmaki M. Supporting health researchers during a pandemic: a research funder's response to COVID-19. MSFHR blog, 14 Dec 2020. Supporting health researchers during a pandemic: A research funder's response to COVID-19 – Michael Smith Foundation for Health Research (MSFHR). Accessed 14 Aug 2021.
63. Witteman HO, Haverfield J, Tannenbaum C. COVID-19 gender policy changes support female scientists and improve research quality. PNAS. 2021; doi: https://doi.org/10.1073/pnas.2023476118.

Germany

Section 1: *Examples of achievements in production of primary and secondary research to inform development of COVID therapies, vaccines, national/local organisational responses; examples (and lessons) about links to existing strengths in the health research system*

In some of its updates, an international living review of COVID-19 projects and funders showed Germany having the 3rd highest funding amount [1]. In terms of funding specifically linked to vaccine R&D, a different team suggested the spending in Germany was second only to that in the US [2]. Analysis of figures from the COVID-19 TrialTracker project up to 8 November 2020 indicated 328 clinical studies, or 5% of the global total, were from Germany [3].

In the years prior to the pandemic, medical academics Uğur Şahin and Özlem Türeci received funding from the government-backed German Research Foundation [4] and the EU [5] to help development of their work on mRNA, from early fundamental scientific questions onwards. This research had led to the creation of their company, BioNTech, in Mainz, Germany [6,7]. They needed considerably more funding and results from early work helped them raise venture capital [5]. The years of research by them, and others described in the US and Canadian sections, brought the mRNA technology to the point where teams at BioNTech and CureVac (another German biotech company), as well as US company Moderna, could immediately start developing their COVID-19 vaccine once the sequence of the genome was released in mid-January 2020 [6,7]. The scientists had often dedicated themselves to following what they thought were good ideas that might eventually improve healthcare, even in the face of much scepticism and sometimes potential career harm [6,7].

BioNTech received a EUR100m loan from the European Investment Bank for its COVID-19 vaccine development [5]. However, BioNTech needed much more capital for vaccine development and went into partnership with US company Pfizer with which it had already been working on an mRNA influenza vaccine (see also section 3) [5]. The company did conduct a key Phase 1/2 clinical trial of their COVID-19 vaccine in Germany in April-May 2020 [8]. The main Pfizer/BioNTech Phase 2/3 trial included six sites in Germany, but many more in the US [9]. In September 2020, BioNTech received a EUR445m grant from the German Government to accelerate its vaccine work, and CureVac was awarded EUR252m for its vaccine candidate (but see section 3) [10].

Research to develop the subsequently widely used reverse transcription polymerase chain reaction (PCR) test for COVID-19 was also conducted and published extremely rapidly by an international, mostly European, team led by Christian Drosten from the Berlin Institute of Virology, Charité Hospital [11]. With funding (presumably existing) for work in the area from the EU and German Ministry of Research, they used their considerable experience, and readiness, to respond as they had to similar viruses earlier [12]. By 16 January 2020 they were able to tell WHO they had produced the PCR test for COVID-19 in days [12] - a rapid development Zhang and Holmes later noted was facilitated by their release of the genome sequence [13]. Drosten's team made a pre-submission enquiry to the editors of *Eurosurveillance,* a leading Open Access journal in the field, who lined up reviewers prepared to conduct double-blind reviews rapidly given the journal's standard practice of attempting to respond flexibly and urgently during evolving public health emergencies where

infections could spread exponentially [14]. The paper was submitted on 21 January 2020, then reviewed, revised and accepted, and published on 23 January 2020 [11].

Drosten supported colleagues in München, including at the University Hospital and the Bundeswehr Institute of Microbiology, who rapidly produced a case report letter published in the *NEJM* on 30 January 2020, on the transmission of COVID-19 infection by an asymptomatic contact in Germany initially identified on 27 January [15]. The immense policy and other implications of this letter meant it attracted enormous attention, and it had been cited over 2,000 times by 31 December 2021. With funding from the research ministry, the EU and the German Bundeswehr Medical Services Biodefence Research Program, a similar team applied the PCR test, and other laboratory techniques including ones they had pioneered, to reveal that the infectious virus could be isolated from nose and throat swabs, even from patients with mild symptoms. This information was released in a joint press release from the three institutions on 4 February 2020 [16], and elaborated in an article submitted to *Nature* on 1 March, published online on 1 April, with Scopus recording 3,000 cites by December 2021 [17].

Scientists at institutions including the Robert Koch Institute (RKI), Germany's public health institute in Berlin under the federal Ministry of Health [18], *"mobilized in early January 2020 to launch a national crisis management effort to understand the epidemiology of the pandemic"* [19]. Early research that fed into policymaking included epidemiological methods such as interviews, whole genome sequencing and antibody testing [19].

Examples of other early studies in which University Hospitals in Germany participated included the REMAP-CAP platform trials where German involvement was led from Jena University Hospital, with EU funding [20-22]. There were also three sites in the international trial of remdesivir (see US section) [23].

Various economics and business departments and institutes, mostly in Germany, published a paper that became important evidence in the debate about NPIs. Entitled *"Face masks considerably reduce COVID-19 cases in Germany"* it used the synthetic control method to analyse the effect of the introduction of mask mandates at different times by local policymakers [24]. Its findings that face masks reduced the number of newly registered cases of COVID-19 by between 15-75% over a period of 20 days from their mandatory introduction was widely cited in Germany and internationally [25,26]. A news feature in *Nature* in May 2021 claimed *"The case for mask mandates was made relatively early in the pandemic",* and described their introduction in Germany, and this research on it [27].

In addition to an early rapid research call to tackle COVID-19, in March 2020 the German Federal Ministry of Education and Research (BMBF) announced EUR150m funding to link all research hospitals into a network to conduct COVID-19 research [28]. It was established as the "Network University Medicine" in April 2020 to include all 34 University Hospitals in Germany working on projects in various combinations. Significantly the network's website said: "*This bundling of competencies and resources is intended to create structures and processes in the clinics that ensure the best possible care for COVID-19 patients*" [29]. In addition to advancing knowledge to deal with the current pandemic, one of the goals of the network was the "*Generation of findings also for better preparation for future epidemiological events"* [29].

It was not until October 2020 that the 13 cross-hospital joint projects to be funded with the EUR150m were announced on a range of COVID-19 topics that continued to receive funding into 2022 [30]. One of the 13 projects, The COVID-19 Evidence Ecosystem (CEOsys), established a national evidence network on COVID-19 led by the Institute for Evidence in Medicine, for Cochrane Germany, at the University Hospital Freiburg. The evidence ecosystem involved 20 university hospitals and several non-university partners coordinating to identify and evaluate scientific findings on a range of COVID-19 issues, and produce a series of interdisciplinary living evidence syntheses. They included ones on: testing and diagnostics, outpatient and inpatient treatment, intensive care and palliative care, hospital hygiene, prevention and public health, and mental health. They served as the basis for living guidelines described in section 3, and the planning for urgently needed further research [31].

Section 2: *Examples of achievements in promoting evidence use in products, policymaking and practice; examples (and lessons) about links to existing strengths*

Despite Pfizer taking the lead on manufacturing the BioNTech/Pfizer vaccine, and receiving a large grant from the US government (see US section), BioNTech's EUR445m grant from the German Government's BMBF was to expand its production capacity in Germany, as well as accelerate vaccine development [5]. This was part of a EUR750m grant to three companies, but see next section. Şahin, Türeci and colleagues, including Katalin Karikó who joined them in 2013 (see US section) also won the 2021 German Future Prize, awarded annually for research achievements that lead to marketable technological developments [4].

How far Germany has been able to draw on scientific evidence to achieve success in controlling the virus has been a matter of some debate (see also next section for challenges), and Lothar Wieler, President of the RKI, and colleagues, claimed in March 2021: *"Germany demonstrates the difficulty of maintaining success throughout the COVID-19 pandemic"* [19]. In terms of the initial response, Wieler et al suggested: "*The country's strong enabling environment, including a good public health care system and expert scientific institutions contributed to the early success"* [19]. In more detail they claimed factors linked to the initial success included Germany's prevention protocols, which facilitated a rapid response to the outbreak, the early development of testing capacity and high levels of testing, and an effective strategy for protecting older people.

The Government had a quite comprehensive National Pandemic Plan prior to the pandemic, although some public health facilities were understaffed and problems were encountered with shortages in personal protection equipment [19]. All 16 states also had plans, but in the early weeks of the pandemic many politicians went beyond what the pandemic plans had defined in terms of consulting experts [32]. For commentators writing in early April 2020, and contrasting Germany's higher rate of testing and much lower case fatality rate with that in other countries such as the UK, *"the country was meticulously prepared for a pandemic"* [33] - a test protocol was rolled out in January 2020 and, when required, the testing was conducted at well over 200 quality controlled laboratories across the country [33,34].

Various cross-country analyses reported favourably on the strengths of the pandemic response in Germany, and the willingness to engage with scientific evidence. The analysis in Germany for the study by Jasanoff et al., was concluded in late December 2020 and stated: *"Germany's public health*

response was characterized by a consistent pattern of delegation of policy questions to scientific authority (especially RKI) and a general appeal to rationality and solidarity" [35]. They also felt that while Christian Drosten, as Germany's most visible scientist and government advisor, was picked out by the tabloid press for criticism, there was considerable public and government pushback against their attacks. The team did, however note that while the reliance on a rather one-dimensional indicator, "7-day incidence", underlined the strong focus on science, it also hinted "*at an unwillingness to consider alternative definitions of risk for the German population"* [35].

The analysis by Han et al., of the COVID-19 response in nine Asian and European countries identified Germany as one of four of the countries where *"experts on infectious diseases within established public-health institutes are responsible for ensuring that scientific evidence drives policy making"* [36]. Similarly, in an analysis of *"Co-producing the covid-19 response"* in Germany and three Asian countries, Marten et al., suggested various existing structures where researchers and policymakers interacted in Germany were part of the pandemic response including scientific advisory boards, research institutes and the RKI [37]. They further suggested the straightforward communication style of some academics and scientists in Germany *"helped to calm an unsettled public and build trust and understanding about why interventions are needed"* [37].

An international analysis published in September 2020 of the role of communications in nine countries thought to have controlled the virus at least reasonably well in the initial phase [38] included a section on Germany [39]. It claimed that *"Scientific expertise has clearly been the foundation for the German approach in dealing with Covid-19 but has also shaped communication strategies"* [39]. This analysis highlighted the important role of scientists such as Drosten and Wieler in communications and providing advice. It agreed with other analyses about the early success also involving leadership by some of the state governments, but the key role was played by Chancellor Merkel [34,39], who *"joined her own scientific expertise with concern and empathy"* [38]. According to the leading international science journal, *Nature,* she acted *"on the basis of expert advice"* [40]. Her own scientific background had, as national leader, established an insistence *"that decision-making benefits from evidence"* which was also compatible with a wider political culture committed to rational responses driven by the scientific data [36,40].

As noted, the situation in Germany became more difficult in later surges of the pandemic. Local analyses published more recently identified a more complex picture suggesting that notwithstanding the initial success, there were also challenges with the narrow composition of the experts committees [32], the availability of data [41] and aspects of the communications [41,42]. These could have contributed to the subsequent difficulties. (See next section.)

Several organisations published types of clinical guidelines, or advice. These included the RKI which updated the advice published on their website over 20 times [43]. The Institute also sometimes cross-referenced to the position stated in other German guidelines, including those produced by the Association of the Scientific Medical Societies in Germany (AWMF).

The AWMF produced living guidelines building on the living evidence syntheses from CEOsys. A major goal of the project was to encourage translation by communicating the findings by using the channels most relevant for specific target groups. The guidelines included national medical ones for the care of COVID-19 patients, but could also be at a population or public health level such as the living guideline on measures for the prevention and control of COVID-19 transmission in schools

[31,44]. Production of the schools' living guideline was led by public health researchers at Ludwig-Maximilians University, München, and involved many medical societies and other organisations. It was based on the Cochrane evidence synthesis on the topic that team members conducted as part of the CEOsys project [45]. The guideline was first published in February 2021, revised several times after that, and endorsed by almost 30 participating societies and organisations [44].

Section 3: *Examples of challenges in: 1. production of evidence; 2. use of evidence; and 3. sustaining the research system; examples of lessons to address challenges*

Challenges in production of evidence

Financial support from the German Government and EU did not seem to be sufficient to enable BioNTech to test and manufacture the new mRNA vaccine without Pfizer playing a major role [5]. The CureVac mRNA vaccine did not perform as well as others in trials. A key lesson from the failure was thought to be that the modified mRNA developed especially through the research in the US of the University of Pennsylvania's team [7], was a vital missing element in the CureVac vaccine, unlike the successful mRNA vaccines [46].

Goossens et al., compared the rapid successes from the RECOVERY trial/ UK arm of REMAP-CAP (see UK section) with the generally slower progress across Europe, but individual countries were not identified [3]. They noted the prioritisation in the UK system, and contrasted the average time in the UK for protocol approval of about a week with a mean of three months across Europe. If partnership across Europe was promoted it could help prioritisation and speed [3].

Challenges in use of evidence

The success in controlling the early waves was not fully continued into later waves of the pandemic [19,36]. Chancellor Merkel *"favoured an early return to tough restrictions – as advised by scientists – but the leaders of many of Germany's powerful state governments refused"* [40]. Wieler et al., too, suggested the second surge saw, *"states deviating from federal recommendations"*, and while the federal system allowed states to tailor their strategies, it *"also limited widespread implementation of a standard testing strategy or national containment measures even in the face of rising case counts"* [19]. The severe fourth wave in late Autumn 2021 was described as *"A Pandemic of the Unvaccinated"*, and it was suggested insufficient attention was given to the warning made in the summer of 2021 by scientists who modelled the impact of a fourth wave and said a higher proportion of the population would need to be vaccinated [47].

Various potential weaknesses in the German response to the pandemic were also subsequently described. A detailed documentary analysis from a public health perspective of the role of expert committees suggested that a lack of transparency made it unclear how far the undoubted creation of such committees to provide evidence, actually influenced policymaking [32]. Furthermore, overall there was a lack of adequate diversity in the membership of such committees in terms of gender and disciplines. This meant the expert committees were *"not sufficiently representative and interdisciplinary to take different perspectives into account"* [32]. This suggestion might support the claim reported above in the analysis in Jasanof et al., about reliance on a one-dimensional indicator [35]. The analysis of expert committees also noted that there was rather more disciplinary diversity

in the expert committees created after the first wave, which might indicate an increased understanding of the complexity and nature of the crisis [32].

Despite the initial success in introducing large-scale testing more rapidly than many other European countries, later, in the face of further surges and increasing disagreements between the federal and state governments, it was suggested that *"the lack of reliable data"* on issues such as variants, might have been one of the reasons behind the surges [41]. There was insufficient systematic data at population level to reflect the effects of the various measures and indicate differences between age groups and the number of unreported cases. The sequencing of virus samples was only ramped up in January 2021 [41].

Furthermore, *"the lack of digital data collection tools and the widespread lack of uniform reporting software"* were seen as major obstacles, including to successful communication. It was suggested that gaps in the understanding of epidemiological figures by politicians and media professionals were particularly problematic given *"the initially very academically oriented scientific policy advice"* [41]. (While just outside the period of our analysis, it is relevant to note that in January 2022, the federal government's Expert Council on COVID-19 produced a statement *"on the need for evidence-based risk and health communication"* that among other points highlighted that a major obstacle, not least for communications, was *"the lack of digitization in the health system in Germany"* [42].)

<u>*Challenges in sustaining the research system*</u>

A survey and workshop conducted in May 2020 of German health researchers who had been working on non-COVID topics reported that 93% of them believed their projects were affected by the pandemic [48]. Eighty percent reported they could not collect data as planned, with problems also caused by staff being unavailable because of care commitments, illness or quarantine. The majority had mitigation strategies in place, including adjustments of data collection through using digital tools, or changing the research design [48]. The authors concluded the main challenge was to mitigate the problems, and *"improve long-term resilience in health research. The pandemic has also acted as a driver of innovation and change"* [48].

Summary

The initial strong pandemic response from Germany's HRS reflected long-term investment in science and a culture of support for evidence-use. Long-term research on mRNA by medical academics Uğur Şahin and Özlem Türeci, partly funded by public and EU sources, had led to the creation of their company, BioNTech. Using their pioneering platform, they immediately started developing their COVID-19 vaccine on the release of the genomic sequence. They went into partnership with US company Pfizer, although they also received some further public funding for continuing work in Germany. The new platform and unprecedented levels of funding and public/private collaboration contributed to greatly accelerated vaccine development. The PCR test was developed by 16 January 2020 by a mostly European collaboration led by Christian Drosten from the Berlin Institute of Virology, Charité Hospital. The test, described in a highly cited publication, was used at the end of January by researchers at München in an analysis that reported asymptomatic transmission of COVID-19. Pre-pandemic planning and the willingness of leaders such as Chancellor Merkel to use the science were widely cited as reasons why Germany responded rapidly through testing and other measures to control the first wave more effectively than many countries. The success in controlling

the early wave was not fully continued into later waves. Chancellor Merkel was not able to persuade all state governments to follow the science. Attention should be given to more recent analysis identifying weaknesses in the German policy and communications response such as the narrow disciplinary background of many expert committees and insufficient relevant data to fully inform policies. Nevertheless, the greater research coordination announced in Spring 2020 by the creation of the “Network University Medicine”, and that had started collaborative projects in October 2020, continued throughout our period until the end of 2021, and beyond.

References

1. Norton A, Bucher A, Antonio E, Advani N, Grund H, Mburu S, et al. A living mapping review for COVID-19 funded research projects: nine-month update [version 4; peer review: 2 approved]. Wellcome Open Research 2021; doi: https://doi.org/10.12688/wellcomeopenres.16259.4.
2. Global Health Centre. 2021. COVID-19 Vaccines R&D Investments. Graduate Institute of International and Development Studies. Retrieved from: knowledgeportalia.org/covid19-r-d-funding Accessed 3 May 2022.
3. Goossens H, Derde l, Horby P, Bonten M. The European clinical research response to optimise treatment of patients with COVID-19: lessons learned, future perspective, and recommendations. Lancet Infect Dis. 2021; doi: https://doi.org/10.1016/S1473-3099(21)00705-2.
4. Deutsche Forschungsgemeinschaft (DFG) (German Research Foundation). DFG congratulates team led by Uğur Şahin on the German Future Prize 2021. Press release 47: 18 Nov 2021. DFG - Deutsche Forschungsgemeinschaft - DFG gratuliert Team um Uğur Şahin zum Deutschen Zukunftspreis 2021. Accessed 10 Jan 2022.
5. Roberts J. Q&A: BioNTech vaccine is only ‘mRNA 1.0’. This is just the beginning, say co-founders | Research and Innovation. Horizon. The EU Research & Innovation Magazine. 8 April 2021. Q&A: BioNTech vaccine is only ‘mRNA 1.0’. This is just the beginning, say co-founders | Research and Innovation (europa.eu). Accessed 12 Aug 2021.
6. Dolgin E. The tangled history of mRNA vaccines. Nature. 2021; doi: https://doi.org/10.1038/d41586-021-02483-w.
7. Allen A. For billion-dollar COVID vaccines, basic government-funded science laid the groundwork. Kaiser Health Newsletter, 18 Nov 2020. For Billion-Dollar COVID Vaccines, Basic Government-Funded Science Laid the Groundwork - Scientific American. Accessed 8 Jan 2022.
8. Şahin U, Muik A, Derhovanessian E, Vogler I, Kranz ML, Vohrmehr M, et al. COVID-19 vaccine BNT162b1 elicits human antibody and T_H1 T cell responses. Nature. 2020; doi: https://doi.org/10.1038/s41586-020-2814-7.
9. Polark FP, Thomas SJ, Kitchin N, Absalon J, Gurtman A, Lockhart S, et al. Safety and efficacy of the BNT162b2 mRNA Covid-19 vaccine. NEJM. 2020; doi: 10.1056/NEJMoa2034577 https://www.nejm.org/doi/full/10.1056/NEJMoa203457.
10. Reuters. BioNTech wins $445 mln German grant for COVID-19 vaccine. Reuters, 15 Sept 2020. BioNTech wins $445 mln German grant for COVID-19 vaccine | Reuters. Accessed 7 Dec 2021.
11. Corman VM, Landt O, Kaiser M, Molenkamp R, Meijer A, Chu DKW, et al. Detection of 2019 novel coronavirus (2019-nCoV) by real-time RT-PCR. Euro Surveill. 2020; doi: https://doi.org/10.2807/1560-7917.ES.2020.25.3.2000045.
12. Charité. Researchers develop first diagnostic test for novel coronavirus in China. Press release. Charité – Universitätsmedizin Berlin. 16 Jan2020. https://www.charite.de/en/service/press_reports/artikel/detail/researchers_develop_first_diagnostic_test_for_novel_coronavirus_in_china/. Accessed 21 Nov 2021.
13. Zhang YZ, Holmes EC. A genomic perspective on the origin and emergence of SARS-CoV-2. Cell. 2020; doi: 10.1016/j.cell.2020.03.035.

14. Eurosurveillance editorial team. Response to retraction request and allegations of misconduct and scientific flaws. Euro Surveill. 2021; doi: https://doi.org/10.2807/1560-7917.ES.2021.26.5.2102041.
15. Rothe C, Schunk M, Sothmann P, Bretzel G, Froeschl G, Wallraunch C, et al. Transmission of 2019-nCoV infection from an asymptomatic contact in Germany. NEJM 2020;382:970-1. https://www.nejm.org/doi/full/10.1056/nejmc2001468
16. Charité, Munich Clinic Schwabing and the Bundeswehr Institute of Microbiology. Novel coronavirus: infectious virus detected in the nose and throat of patients with mild symptoms. Press release, 4 Feb 2021. Novel coronavirus: Infectious virus detected in the nose and throat of patients with mild symptoms: Charité – Universitätsmedizin Berlin (charite.de). Accessed 30 Dec 2021.
17. Wölfel R, Corman VM, Guggemos W, Seilmaier M, Zange S, Muller MA, et al. Virological assessment of hospitalized patients with COVID-2019. Nature 2020; doi: https://doi.org/10.1038/s41586-020-2196-x.
18. Robert Koch Institut (RKI). The Robert Koch Institute. https://www.rki.de/EN/Content/Institute/institute_node.html;jsessionid=CB117B88EE5935585ACCCCD21E0FC4C1.internet062. Accessed 11 Jan 2022.
19. Wieler LH, Ute R, Gottschalk R. Emerging COVID-19 success story: Germany's push to maintain progress. Exemplars in Global Health platform. 2021. Emerging COVID-19 success story: Germany's push to maintain progress - Our World in Data. Accessed 14 Aug 2021.
20. REMAP-CAP. REMAP-CAP: A randomised, embedded, multi-factorial, adaptive platform trial for community-acquired pneumonia. REMAP-CAP Trial (remapcap.org). Accessed 7 Jan 2022.
21. The Writing Committee for the REMAP-CAP Investigators. Effect of hydrocortisone on mortality and organ support in patients with severe COVID-19: The REMAP-CAP COVID-19 corticosteroid domain randomized clinical trial. JAMA. 2020; doi:10.1001/jama.2020.17022.
22. The REMAP-CAP Investigators. Interleukin-6 receptor antagonists in critically ill patients with Covid-19. N. Engl. J. Med. 2021;384:1491-1502.
23. Beigel JH, Tomashek KM, Dodd LE, Mehta, AK, Zingman BS, Kalil AC, et al. Remdesivir for the treatment of Covid-19 — final report. NEJM. 2020; doi: 10.1056/NEJMoa2007764.
24. Mitze T, Kosfeld R, Rode J, Wälde K. Face masks considerably reduce COVID-19 cases in Germany. Proc. Natl Acad. Sci. 2020; doi: https://doi.org/10.1 073/pnas.2015954117.
25. PNAS. Altmetrics for "Face masks considerably reduce COVID-19 cases in Germany" https://pnas.altmetric.com/details/95397995/news Accessed 26 March 2022.
26. Van Schalkwyk MCI, McKee M. Research into policy: lessons from the COVID-19 pandemic. Eur J Public Health. 2021; doi: https://doi.org/10.1093/eurpub/ckab155.
27. Peeples L. What the science says about lifting mask mandates. Nature News Feature, Nature. 2021; doi: https://doi.org/10.1038/d41586-021-01394-0.
28. Germany Trade & Invest. Germany Devotes EUR150 Million to Connect Research Hospitals into Anti-Corona Network. Germany Trade & Invest. 26 March 2020. https://www.prnewswire.co.uk/news-releases/germany-devotes-eur150-million-to-connect-research-hospitals-into-anti-corona-network-855147045.html Accessed 9 June 2022.
29. Network University Medicine. Tasks and Objectives: Answer to Unexpected Challenges. https://www.netzwerk-universitaetsmedizin.de/aufgaben-und-ziele Accessed 10 June 2022.
30. Federal Ministry of Education and Research (BMBF). Karliczek: Netzwerk Universitätsmedizin startet vertiefte Forschungsarbeit zu Covid-19. BMBF press release 141/2000. https://www.bmbf.de/bmbf/shareddocs/pressemitteilungen/de/karliczek-netzwerk-universitae-e-forschungsarbeit-zu-covid-19.html Accessed 10 June 2022.
31. Network University Medicine. CEOsys | Aufbau eines COVID-19 Evidenz-Ökosystems zur Verbesserung von Wissensmanagement und Translation (Building a COVID-19 Evidence Ecosystem to improve knowledge management and translation). BMBF. https://www.netzwerk-universitae tsmedizin.de/projekte/ceo-sys#c501 Accessed 10 June 2022.

32. Sell K, Saringer-Hamiti L, Geffert K, Strahwald B, Stratil JM, Pfadenhauer LM. Politikberatung durch Expert*innenräte in der SARS-CoV-2-Pandemie in Deutschland: Eine Dokumentenanalyse aus Public-Health-Perspektive. (Expert committees in German public health policymaking during the SARS-CoV-2 pandemic: a document analysis). Z. Evid. Fortbild. Qual. Gesundh. wesen (ZEFQ), 2021; 165:1–12. DOI:https://doi.org/10.1016/j.zefq.2021.06.002
33. Eckner C. How Germany has managed to perform so many Covid-19 tests. The Spectator. April 6, 2020. https://www.spectator.co.uk/article/how-germany-has-managed-to-perform-so-many-covid-19-tests. Accessed 21 Nov 2021.
34. Stafford N. Covid-19: why Germany's case fatality rate seems so low. BMJ. 2020; doi: https://doi.org/10.1136/bmj.m1395.
35. Beck S, Nardmann J, Pfotenhauer S, van Galen T. Germany. In: Jasanoff S, Hilgartner SJ, Hurlbut JB, Ozgode O, Rayzberg M. Comparative Covid response: crisis, knowledge, politics. Interim report. 12 Jan 2021. https://compcore.cornell.edu/wp-content/uploads/2021/03/Comparative-Covid-Response_Crisis-Knowledge-Politics_Interim-Report.pdf. Accessed 12 Jan 2022.
36. Han E, Tan MMJ, Turk E, Sridhar D, Leung GM, Shibuya K, et al. Lessons learnt from easing COVID-19 restrictions: an analysis of countries and regions in Asia Pacific and Europe. Lancet. 2020; doi: https://doi.org/10.1016/ S0140-6736(20)32007-9.
37. Marten R, El-Jardali F, Hafeez H, Hanefeld J, Leung GM, Ghaffar A. Co-producing the covid-19 response in Germany, Hong Kong, Lebanon, and Pakistan. BMJ. 2021;372 doi: https://doi.org/10.1136/bmj.n243.
38. Tworek H, Beacock I, Ojo E. Democratic Health Communications during Covid-19: A RAPID Response. Vancouver: UBC Centre for the Study of Democratic Institutions, September 2020. Democratic-Health-Communication-during-Covid_FINAL.pdf (ubc.ca) Accessed 4 May 2022.
39. Metzger D, David-Wilp S. Germany. In: Tworek H, Beacock I, Ojo E. Democratic Health Communications during Covid-19: A RAPID Response. Vancouver: UBC Centre for the Study of Democratic Institutions, September 2020; 72-6. Democratic-Health-Communication-during-Covid_FINAL.pdf (ubc.ca) Accessed 4 May 2022.
40. Nature Editorial. Politics will be poorer without Angela Merkel's scientific approach. Nature. 2021;597;304. doi: https://doi.org/10.1038/d41586-021-02479-6.
41. Kurth T, Brinks R. Poorly prepared for the pandemic. Spektrum, Health Care. 4 June 2021. https://www.spektrum.de/news/gesundheitsfuersorge-schlecht-vorbereitet-in-die-pandemie/1868491 Accessed 10 June 2022.
42. Federal Government's Expert Council on COVID-19. Statement on the need for evidence-based risk and health communication. Expert Council on COVID-19. 1 January 2022. https://www.bundesregierung.de/resource/blob/974430/2002168/e9930873d3958ad6c2ee153c8bc319a5/2022-01-30-fuenfte-stellungnahme-expertenrat-data.pdf?download=1 Accessed 10 June 2022.
43. Permanent Working Group of Competence and Treatment Centers for High Consequence Infectious Diseases. Notes on the identification, diagnosis and treatment of patients with COVID-19 Last update 16/07/2021. Robert Koch Institute. www.rki.de/covid-19-therapie-stakob. Accessed 30 Dec 2021.
44. Association of the Scientific Medical Societies in Germany (AWMF). Measures for the prevention and control of SARS-CoV-2 transmission in schools. Living Guideline. Short Version. AWMF. Content as at version 1.1, Nov 2021. 027-076eng_prevention_and_control_SARS-CoV-2_transmission_in_schools_2022-03.pdf (awmf.org) Accessed 11 May 2022.
45. Krishnaratne S, Littlecott H, Sell K, Burns J, Rabe JE, Stratil JM, et al. Measures implemented in the school setting to contain the COVID-19 pandemic. Cochrane Database of Systematic Reviews 2022, Issue 1. Art. No.: CD015029. DOI: 10.1002/14651858.CD015029. Measures implemented in the school setting to contain the COVID-19 pandemic - Krishnaratne, S - 2022 | Cochrane Library Accessed 11 May 2022.

46. Dolgin E. CureVac COVID vaccine let-down spotlights mRNA design challenges. Nature.2021; doi: https://doi.org/10.1038/d41586-021-01661-0.
47. Bennhold K. Germany's fourth Covid wave: 'A pandemic of the unvaccinated'. New York Times, 11 Nov 2021 https://www.nytimes.com/2021/11/11/world/europe/germany-covid-unvaccinated.html. Accessed 20 Nov 2021.
48. Bratan T, Aichinger H, Brkic N, Rueter J, Apfelbacher C, Boyer L, et al. Impact of the COVID-19 pandemic on ongoing health research: an ad hoc survey among investigators in Germany. BMJ Open. 2021; doi: 10.1136/ bmjopen-2021-049086.

New Zealand

Section 1: *Examples of achievements in production of primary and secondary research to inform development of COVID therapies, vaccines, national/local organisational responses; examples (and lessons) about links to existing strengths in the health research system*

The Ministry of Health and the Health Research Council (HRC) launched a rapid research response to the COVID-19 threat in February 2020 even before there were any local cases [1]. In launching the NZD3m call, Sunny Collings, HRC Chief Executive Officer, said, in addition to contributing to the global effort, *"We aim to improve New Zealand's responsiveness and readiness for disease outbreak within New Zealand's borders"*.

Projects that focused on local priorities included those from the joint call and some funded additionally by the HRC [2] (plus other funding sources). They included studies on the specific needs of the Indigenous Māori during the pandemic, and a project aimed at creating a new approach to the use of genomic data to understand the spread of the disease [2].

Papers included one with funding also from other sources, including the Ministry of Business Innovation and Employment's COVID 19 Innovation Acceleration Fund, that used genetic epidemiology to reveal transmission patterns and the dynamics of COVID-19 in New Zealand, thus helping to quantify the effectiveness of public health interventions such as lockdowns [3]. This paper in *Nature Communications* was written by a team from various institutions mostly in New Zealand, but including international authors, e.g., from the Doherty Institute in Australia. The paper was devised by Jemma Geoghegan who wrote the initial draft with Edward Holmes (see Australian section). They were both also co-authors on a paper led by Tara Swadi, chief advisor on COVID-19 at the Ministry of Health, analysing in-flight transmission of COVID. This generated considerable international interest [4]. Findings from these studies combined with other data were extensively used by government agencies [5]. (See next section.)

The titles of some papers from rapidly funded projects reflected the overall successful team approach to tackling COVID, but attempted to understand attitudes in one area where there was less consensus. For example, one paper was called *"Wearing one for the team: views and attitudes to face covering in New Zealand..."* [6].

Under the leadership of Colin McArthur, New Zealand sites already played an active role in the international REMAP-CAP clinical trial. As part of the support for the global effort, funding and hospital sites were provided for REMAP-CAP COVID-19 arms [2]. These included the study of corticosteroids and also the meta review to which it contributed; they were simultaneously published in the *JAMA*, with McArthur as a co-author, showing and reinforcing the benefits of using corticosteroids in treating critically ill patients with COVID-19 [7,8].

Notwithstanding policy successes (see next section) leading to relatively few patients available for recruitment [9], the HRC was able to celebrate New Zealand's contribution to the very rapid global research progress, and the continuing work on the REMAP-CAP project included contributing to the findings on the benefits from IL-6 receptor antagonists [10,11]. The HRC call also helped fund the ASCOT study [12]. (See Australian section above.)

Much of the overall coherence and focus of the COVID-19 research funded reflected, or at least was compatible with, New Zealand's comprehensive health research strategy for 2017-2027 with its focus on researching the priorities of relevant local stakeholders [13]. This included the priority of promoting equity in the pandemic response: a specific part of this call was for Māori-led research and was intended to support equitable health and wellbeing outcomes for Māori [14]. In 2021 the leadership that had been shown by Māori in managing the health and wellbeing needs of their communities during the pandemic helped drive a further new equity research programme *"into a much-needed shift in the health system"* [15]. This, in turn, was entirely compatible with part of the vision of the health research strategy to partner with Māori communities to improve Māori health through research and boost the Māori health research sector [13]. (But see section 3.)

With funding from the two relevant ministries listed above, Sarah Jefferies and colleagues at the Institute for Environmental Science and Research and elsewhere, including a Ministry of Health colleague, used highly complete prospectively collected COVID-19 case and testing datasets [16]. They claimed, *"This is the first study to our knowledge to assess the impacts of national or subnational non-pharmaceutical intervention escalation and de-escalation decisions on the distribution, transmission patterns, and severity of COVID-19, and the attainment of an explicit national goal of COVID-19 elimination"* [16].

The study led to clear findings that strikingly reinforced the value of the rapid-evidence informed policy described in the next section: *"New Zealand's response resulted in low relative burden of disease, low levels of population disease disparities, and the initial achievement of COVID-19 elimination"* [16]. The paper, published in *Lancet Public Health*, attracted considerable attention and had been cited about 80 times by the end of 2021. It is possible that while the low level of cases in New Zealand made trials more challenging, it facilitated the comprehensive detailed analysis described here.

Jefferies, who had also been a co-author on both the papers above on the spread of the virus [3,4], and colleagues won a HRC medal for their research, with Collings pointing out that the team wrote the paper and also provided real time analysis and support to the Ministry of Health. Collings continued: "*New Zealand's relatively small health research workforce has worked tirelessly together with other health professionals to provide fast and accurate information to try to minimise the impact of COVID-19 on our communities"* [17].

Section 2: *Examples of achievements in promoting evidence use in products, policymaking and practice; examples (and lessons) about links to existing strengths*

The health research strategy's priority of *"translating research findings into policy and practice"* [13] contributed to the context in which PM Ardern highlighted the importance of both science and her Chief Scientific Adviser: *"science, scientists and science communicators have been at the forefront of the Government's... fight to eliminate COVID-19...provide me with advice about the way forward, and to connect me and other Ministers with the range of scientific experts and communicators, both in New Zealand, and overseas"* [18]. The country was able to take full advantage of its relatively isolated location and ability to close its borders.

A more detailed account of how the science was used appeared in *Nature Immunology* under the title, *"New Zealand's science-led response to the SARS-CoV-2 pandemic"* [5]. It explained: *"The New*

Zealand government's use of scientific expertise, spanning public health, infectious diseases, genomics modelling and immunology, has been one of the keys to the success of its SARS-CoV-2 elimination and control strategy" [5]. The findings of some studies, as described in Section 1, had elements of co-production, and data gathered were *"used by government agencies to direct public health interventions, highlight transmission hotspots to target community testing, identify superspreading events and assess the impact of interventions such as travel restrictions and border closures"* [5].

In the COVID Notes series in the *NEJM,* expert advisors also highlighted New Zealand's *"Rapid, science-based risk assessment linked to early, decisive government action"*, and the PM's effective communication of key messages [19]. Highlighting the general perception of the importance of effective communications, Siouxsie Wiles, a biomedical scientist with expertise in science communications, became an advisor to the PM and won the New Zealander of the Year Award in 2021 [20]. Success in controlling the pandemic was underlined by New Zealand having no excess deaths even by the end of 2021 [21,22].

Policy analysts from New Zealand described the importance of mobilising expertise to deliberate on public policies. They said lessons could be learnt by contrasting PM Ardern's successful science-based approach with that in the US and the UK [23]. Jefferies et al., concluded that their own study *"supports WHO recommendations for timely decisive government leadership for evidence-informed, risk-based escalation and de-escalation decisions"* [16]. A commentary on the Jefferies et al., paper in the same journal reinforced this message by stating that New Zealand's experience highlighted that successful NPIs "*rely on early decisive reactions from health authorities, performant surveillance systems, and targeted strategies as much as stringency"* [24].

The importance of New Zealand's successful communications of the evidence-based policies was highlighted in the cross-country analysis of communications in countries that had been at least reasonably successful in saving lives. While the overall analysis suggested clear evidence-based messaging was a feature of most of the countries, it specifically identified New Zealand as one of the cases that had shared epidemiological modelling data with the population [25]. Two of the report's major takeaways in relation to New Zealand were: *"Communications were a critical intervention: centrepiece of government response was a four-stage alert system for lockdown measures, introduced and explained clearly to citizens before restrictions were put into effect...Messaging critically reinforced by Prime Minister Jacinda Ardern: with a background in communications, she was constantly accessible, clear, patient, and empathetic"* [26].

Section 3: *Examples of challenges in: 1. production of evidence; 2. use of evidence; and 3. sustaining the research system; examples of lessons to address challenges*

Challenges in production of evidence

As noted, one limitation reflected policy successes: low case numbers restricted recruitment to the clinical trials funded in the main initial HRC-funding call (see section 1). Two of the three were international, ASCOT and REMAP-CAP, but the third, a New Zealand trial of HCQ prophylaxis in frontline healthcare workers did not proceed beyond the trial set-up phase because of the emerging lack of efficacy for HCQ for COVID-19, and a replacement drug was reported to be unnecessary because of the fall in cases [9].

Challenges in use of evidence

At the outset there was a major challenge in terms of *"a general lack of planning, public health investment and readiness"* [27]. As shown on Table 1 of the main paper, the GHS Index published in October 2019 showed New Zealand lacked the capacity of other high income countries included in our study [28]. Wiles thought the lack of readiness illustrated by the rank on the GHS survey encouraged a science-based decision-making process to lockdown [29]. She explained: *"countries that thought they were prepared have done very badly...We knew our testing and hospital capacity were really bad... we couldn't just rely on testing and contact tracing"* [29].

Many of the experts who had supported the approach hitherto were surprised at the apparent comparatively limited consultation when the PM announced a change in strategy in October 2021 [30]. This change was also strongly criticised by leaders of the Māori and Pacific communities [31]. Seemingly unlike earlier, these communities by then had a disproportionately large number of the overall low number of cases. They were also disproportionately likely to be hospitalised by COVID-19, had lower vaccination rates, and had a potentially greater vulnerability to the virus which was thought to have been a motive for the original elimination strategy adopted early in the pandemic [32]. Following international spikes in the pandemic, and further variants, in the end New Zealand continued its cautious approach throughout 2021. Table 1 from the main paper shows the number of deaths remained very low throughout 2020 and 2021 and a high rate of vaccination was achieved by the end of 2021 [33]. (The high rate of vaccination meant that though the number of cases surged in several waves as Omicron rapidly spread once the restrictions were relaxed, the death rate remained much lower than in most other countries across the globe despite increasing from its extremely low level.)

Challenges in sustaining the research system

In late December 2021 the HRC announced additional funding for emerging researchers who, as elsewhere, had been most vulnerable to being exposed to career challenges because of the pandemic [34].

Summary

New Zealand researchers made a useful contribution to the international REMAP-CAP trial of drugs that might be repurposed to treat COVID-19. However, the HRS's main contribution came through working broadly as set out in New Zealand's comprehensive health research strategy for 2017-2027 with its focus on researching the priorities of relevant local stakeholders. The strategy's vision included the priority of promoting equity, and the pandemic response included a specific call for Māori-led research intended to support equitable health and wellbeing outcomes for Māori. A series of projects related to the needs of policymakers to understand the nature of the disease in the country, to the small extent it was present in 2020 and 2021, and the effectiveness of the NPIs. The research findings were then often effectively used and communicated, sometimes with researchers simultaneously working alongside policymakers. In New Zealand, the health research strategy's priority of research findings being translated into policy and practice contributed to the context in which PM Ardern highlighted the importance of both science and her Chief Scientific Adviser to achieve the aim of eliminating COVID-19. There seemed to be a combination of relying on scientific advice at the outset, and then increasingly also on (co-produced) findings from local research as well

as continuing use of international evidence. PM Ardern's effective evidence-based policymaking and communications were widely praised. They were seen as a major factor in the country controlling the virus well throughout 2020, and also in 2021 as high vaccination rates were achieved.

References

1. Health Research Council (HRC). Government agencies launch rapid research response to COVID-19 threat. HRC, New Zealand, 24 Feb, 2020. https://www.hrc.govt.nz/news-and-events/government-agencies-launch-rapid-research-response-covid-19-threat. Accessed 6 Aug 2022.
2. HRC. New research studies set to help local and global fight against COVID-19. HRC, New Zealand, 17 Apr, 2020. New research studies set to help local and global fight against COVID-19 | Health Research Council of New Zealand (hrc.govt.nz). Accessed 17 Jan 2022.
3. Geoghegan JL, Ren X, Storey M, Hadfield J, Jelley L, Jefferies S, et al. Genomic epidemiology reveals transmission patterns and dynamics of SARS-CoV-2 in Aotearoa New Zealand. Nat Commun. 2020; doi: https://doi.org/10.1038/s41467-020-20235-8.
4. Swadi T, Geoghegan JL, Devine T, McElnay C, Shoemack P, Xiaoyun R, et al. A case study of extended in-flight transmission of SARS-CoV-2 en route to Aotearoa New Zealand. Emerg Infect Dis. 2021; doi: 10.3201/eid2703.204714
5. Geoghegan JL, Moreland NJ, Le Gros G, Ussher JE. New Zealand's science-led response to the SARS-CoV-2 pandemic. Nat Immunol. 2021; doi: https://doi.org/10.1038/s41590-021-00872-x.
6. Gray L, MacDonald C, Tassell-Matamua N, Stanely J, Kvalsvig A, Zhang J, et al. Wearing one for the team: views and attitudes to face covering in New Zealand/Aotearoa during COVID-19 Alert Level 4 Lockdown. J Prim Health Care. 2020; doi:10.1071/HC20089.
7. The Writing Committee for the REMAP-CAP Investigators. Effect of hydrocortisone on mortality and organ support in patients with severe COVID-19: The REMAP-CAP COVID-19 corticosteroid domain randomized clinical trial. JAMA. 2020; doi:10.1001/jama.2020.17022.
8. The WHO Rapid Evidence Appraisal for COVID-19 Therapies (REACT) Working Group. Association between administration of systemic corticosteroids and mortality among critically ill patients with COVID-19. A meta-analysis. JAMA. 2020; doi: https://dx.doi.org/10.1001%2Fjama.2020.17023
9. HRC. Update on COVID-19 clinical trials. HRC, New Zealand, 17 Feb 2021. https://hrc.govt.nz/news-events/update-new-zealands-rapid-response-covid-19-clinical-trials. Accessed 12 Jan 2022.
10. HRC. Studies show improved outcomes for COVID-19 patients receiving steroid treatments. HRC, 2 Sept 2020. Studies show improved outcomes for COVID-19 patients receiving steroid treatments | Health Research Council of New Zealand (hrc.govt.nz). Accessed 7 Dec 2021.
11. The REMAP-CAP Investigators. Interleukin-6 receptor antagonists in critically ill patients with Covid-19. N. Engl. J. Med. 2021;384:1491-1502.
12. ASCOT. AustralaSian COVID-19 trial. https://www.ascot-trial.edu.au. Accessed 10 Dec 21.
13. Ministry of Business, Innovation and Employment (MBIE) /Ministry of Health. New Zealand health research strategy 2017–2027. Wellington: Ministry of Business, Innovation and Employment/Ministry of Health; 2017. nz-health-research-strategy-jun17.pdf Accessed 17 Nov 2021.
14. HRC. New call for proposals now open: COVID-19 equity response. HRC, New Zealand, 4 Aug, 2020. https://mailchi.mp/hrc/new-covid-19-call-opens-today-equity-focus?e=69b9329363. Accessed 16 Aug 2021.
15. HRC. Developing the knowledge for a health system shift towards equity, 27 July, 2021. Developing the knowledge for a health system shift towards equity | Health Research Council of New Zealand (hrc.govt.nz). Accessed 15 Aug 2021.
16. Jefferies S, French N, Gilkison C, Graham G, Hope V, Marshal J, et al. COVID-19 in New Zealand and the impact of the national response: a descriptive epidemiological study. Lancet Public Health. 2020; doi: https://doi.org/10.1016/S2468-2667(20)30225-5.

17. HRC. COVID-19 research team earns Liley Medal for landmark study on NZ's pandemic response. HRC, 18 Nov 2021. COVID-19 research team earns Liley Medal for landmark study on NZ's pandemic response | Health Research Council of New Zealand (hrc.govt.nz). Accessed 7 Dec 2021.
18. Ardern J. Opening Remarks. In: Mahi Tahi 2. Annual Report 2020. Auckland, New Zealand: Office of the Prime Minister's Chief Scientific Advisor. 2020. https://cpb-ap-se2.wpmucdn.com/blogs.auckland.ac.nz/dist/f/688/files/2020/01/PMCSA-Annual-Report-2020-web-version_-to-upload-v4.pdf Accessed 15 Aug 2021
19. Baker MG, Wilson N, Anglemyer A. Successful elimination of Covid-19 transmission in New Zealand. NEJM. 2020; doi:10.1056/NEJMc2025203
20. McCallum H. Dr. Siouxsie Wiles named 2021 New Zealander of the Year. Stuff, 31 March 2021. Dr Siouxsie Wiles named 2021 New Zealander of the Year | Stuff.co.nz. Accessed 7 Dec 2021.
21. Giattino C, Ritchie H, Roser M, Ortiz-Ospina E, Hasell J. Excess mortality during the Coronavirus pandemic (COVID-19) –[data for end of 2021]. Excess mortality during the Coronavirus pan/demic (COVID-19) - Statistics and Research - Our World in Data. Accessed 10 Aug 2022.
22. Ritchie H, Mathieu E, Rodés-Guirao L, Appel C, Giattino C, Ortiz-Ospina E, et al. Coronavirus pandemic (COVID-19). Published online at OurWorldInData.org . Retrieved from: https://ourworldindata.org/coronavirus Accessed 10 Aug 2022
23. Mazey S, Richardson J. Lesson-Drawing from New Zealand and Covid-19: the need for anticipatory policy making. The Political Quarterly. 2020; doi: http://dx.doi.org/10.1111/1467-923X.12893.
24. Robert A. Lessons from New Zealand's COVID-19 outbreak response. Lancet Public Health. 2020; doi: https://doi.org/10.1016/S2468-2667(20)30237-1
25. Tworek H, Beacock I, Ojo E. Democratic Health Communications during Covid-19: A RAPID Response. Vancouver: UBC Centre for the Study of Democratic Institutions, September 2020. Democratic-Health-Communication-during-Covid_FINAL.pdf (ubc.ca) Accessed 4 May 2022.
26. Beacock I. New Zealand. In: Tworek H, Beacock I, Ojo E. Democratic Health Communications during Covid-19: A RAPID Response. Vancouver: UBC Centre for the Study of Democratic Institutions, September 2020; 72-6. Democratic-Health-Communication-during-Covid_FINAL.pdf (ubc.ca) Accessed 4 May 2022.
27. Gorman D, Horn M. On New Zealand's weak, strong and muddled management of a COVID-19 epidemic. Intern Med J. 2020;80:901-4.
28. Global Health Security Index (2019). Nuclear Threat Initiative (NTI) and John Hopkins. Oct 2019. https://www.ghsindex.org/wp-content/uploads/2021/11/2019-Global-Health-Security-Index.pdf. Accessed 8 Dec 2021.
29. Global Health Security Index. Top New Zealand health official cites GHS index as key to country's successful COVID-19 response. News Item, 3 Mar 2021. Top New Zealand Health Official Cites GHS Index as Key to Country's Successful COVID-19 Response - GHS Index. Accessed 6 Nov 2021.
30. McClure T. New Zealand government's pivot from Covid elimination 'surprised' top health experts. The Guardian, 12 Oct 2021. New Zealand government's pivot from Covid elimination 'surprised' top health experts (msn.com) Accessed 12 Oct 2021.
31. McClure T. Māori party warns reopening New Zealand amid Covid outbreak would be 'modern genocide'. Guardian 11 Oct 2021. Māori party warns reopening New Zealand amid Covid outbreak would be 'modern genocide' | New Zealand | The Guardian. Accessed 11 Oct 2021.
32. Baker MG, Kvalsvig A, Verrall AJ. New Zealand's COVID-19 elimination strategy. Med J Aust. 2020; doi: https://doi.org/10.5694/mja2.50735.
33. Mathieu, E., Ritchie, H., Ortiz-Ospina, E. et al., A global database of COVID-19 vaccinations. Nat Hum Behav. 2021;5:947–953. Web update for 31 Dec 2021: Coronavirus (COVID-19) Vaccinations - Our World in Data Accessed 10 Aug 2022.

34. HRC. Extra $6.8M for emerging researchers impacted by COVID-19. HRC, 22 Dec 2021. Extra $6.8M for emerging researchers impacted by COVID-19 | Health Research Council of New Zealand (hrc.govt.nz). Accessed 17 Jan 2022.

United Kingdom

Section 1: ***Examples of achievements in production of primary and secondary research to inform development of COVID therapies, vaccines, national/local organisational responses; examples (and lessons) about links to existing strengths in the health research system***

On 4th February 2020 the National Institute for Health Research (NIHR) and UK Research and Innovation (covering all the UK Research councils, including the Medical Research Council (MRC)) launched a GBP20m COVID-19 research call, the first of many the funders would make over the next two years [1]. In the 12 months from April 2020 there were over one million participants in 101 studies prioritised with Urgent Public Health status [2], often established with the involvement of NIHR's Biomedical Research Centres, including at Oxford [3], as well as MRC-funded centres, and delivered in the NIHR's Clinical Research Network (CRN) [2,4].

<u>*Therapies*</u>

The first site was set up on average three days after a study was approved. A researcher quoted said "*it's as if someone's taken a time warp machine to it"* [4]. All Trusts within the National Health Service (NHS) delivered NIHR CRN portfolio studies [2]. The initial intense centralisation of prioritisation at national, and then local, levels was facilitated by the existing NIHR structure built up over many years. The prioritisation meant in most cases researchers in NHS hospitals were, in practice, initially able to set up (or often even conduct) research on only a few centrally selected key COVID Urgent Public Health studies [4-6]. The list was selected by a small group of key players [4] and agreed across the governments of the UK nations (i.e., for Scotland, Wales and Northern Ireland their own administrations, and for England the UK Government). It covered projects from among the many projects proposed by researchers. They had various funders including the MRC, not just the NIHR. The first six priority projects included RECOVERY for repurposing drugs [7], with REMAP-CAP soon added [8].

The RECOVERY adaptive platform trial was created rapidly at Oxford by Peter Horby and Michael Landray whose affiliations included NIHR and MRC funded centres, and who had already been exploring ways of speeding up clinical trials. Ethics review and other procedures were accelerated: it took just nine days from protocol finalisation to recruitment of the first patient. With its brief protocols the RECOVERY trial was implemented as a priority study *"using the amazing clinical network in the NHS built up over many years"* and recruited its first 1,000 patients in 16 days [5,9-12].

Many factors also contributed to the rapid findings from what became the world's largest COVID-19 trial. These included the large number of UK cases: *"the United Kingdom's own bungled public health response to the new virus, which has led to Europe's largest outbreak, has been taken advantage of by Recovery"* [13]. Further reasons included: the harnessing of over 25 different datasets, including through repurposing of the recently created NHSDigiTrials which minimised the burden for patients and staff [14], and, more broadly, the overall coordination possible with the integrated healthcare system, and buy-in from stakeholders at every level into a study seen as embracing *"quality-by-design*" and addressing urgent needs [4,5,9,10].

A press release from the RECOVERY team at lunchtime on 16 June 2020 announced dexamethasone could reduce deaths [15]; it was in use in the NHS later that day (see next section) and published as a pre-print a week later and online in the *NEJM* on 17 July [13,16]. It made a key contribution to WHO's (UK-led) rapid appraisal of corticosteroids (57% weight) [17], and the living guideline [18]. In the summer of 2020, the enormous scale of potential health and economic benefits from use of dexamethasone as a COVID-19 therapy were estimated [19]. RECOVERY equally rapidly showed some suggested novel therapies, e.g., HCQ, did not work [20], and contributed 74% weight to the largest early meta-analysis [21]. Publication of findings from RECOVERY [22] and REMAP-CAP [23] showing the value of IL-6 receptor antagonist triggered a further update of WHO's living guideline, with RECOVERY again providing most cases [24]. Both trials also reported further findings.

While REMAP-CAP had sites across the globe, at one point 65% of total patients in REMAP-CAP arms were in UK NIHR CRN sites [8]. This was partly because in other countries REMAP-CAP faced competition from local studies in addition to often facing greater delays than in the UK before being allowed to start enrolling patients [11]. Horby, contrasting the ongoing success in the UK of RECOVERY and REMAP-CAP platform trials with developments elsewhere, commented: *"There's been a huge amount of wasted resources...it's not surprising because we're in a pandemic and everybody wants to try and do something"* [9].

Despite some concerns about aspects of the speed of the RECOVERY trial, and implications for other possible trials, (see section 3), there was considerable support from clinicians [4,5], and wide international attention and approval [11,13,25-29]. This included noticing *"the trial was remarkably inexpensive and has proved its value"* [29].

However, it would not have been feasible to have achieved the rapid success without the resources that had been committed since 2006 to building up the NIHR/CRN infrastructure [30,31] which "*enabled NHS hospitals to deliver research of global importance at unprecedented pace"* [4]. In addition to that general observation, Wyatt et al., also analysed in detail what happened in their large NHS Acute Trust, which included a group of major research hospitals. They explained *"how the embedded research system was adapted and repurposed to support the COVIID-19 response....The Trust and national COVID-19 response entailed a rapid large-scale reorganisation of research staff, research infrastructure and research priorities"* [4]. So, while the existing NIHR/CRN infrastructure was necessary, other factors involved included the prioritisation being much more severe than usual, and some research capacity being redeployed to focus on the priority projects as other research staff were redeployed to provide extra clinical support in the initial crisis. All this was achieved with no pre-planning within the Trust [4].

Having a unified national healthcare system and centralised research prioritisation also meant the UK was more able to resist various overlapping aspects of what became labelled as *"hype-based medicine"*, i.e., the demand for unproven treatments driven especially by ill-informed parts of the media and political leadership [11,32]. One aspect of this was the unstructured but widespread patient demand for treatments such as HCQ, with which some clinicians acquiesced, and another was the explosion of small, duplicate trials of such drugs as certain clinicians, sometimes with very limited experience of research, desperately searched for an effective cure whilst coping with patient pressure [9,11,32,33].

In a letter to clinicians in the NHS in April 2020, the Chief Medical Officers across the UK nations, including Chris Whitty in England who also headed the NIHR, not only encouraged them to include patients in the priority trials of repurposed drugs as potential therapies, but also strongly discouraged the use of off-licence treatments outside of trials [5,26]. The NIHR itself claimed, *"By being embedded in the NHS, we have been able to respond quickly and prioritise research based on science, relevance and feasibility"* [34]. This continued with additional COVID-19 research topics of concern to the healthcare system regularly being identified, including treatments for long-COVID [35], and whether COVID-19 vaccines should be administered with seasonal influenza vaccines [36]. A prospective study of early UK responses to the pandemic commented on *"research readiness"* and concluded *"the UK was particularly well prepared, in global terms, to respond rapidly with, for example, clinical trials*" [37].

Oxford/AstraZeneca Vaccine

The success of Sarah Gilbert, Catherine Green and many others in developing the Oxford vaccine was based on years of work on vaccines at the university's Jenner Institute [38]. This included creating a platform which would enable them to respond more rapidly than ever before to a new virus, Disease X. In early January 2020, SARS-Cov-2 turned out to be Disease X. Even before the publication of the sequence of the new virus's genome on 11 January, Gilbert and colleagues such as Teresa Lambe had started considering the implications of the new virus. They had liaised with international colleagues, such as in the National Institutes of Health (NIH) in the US, and decided to use their new platform to develop a vaccine as soon as the genetic sequence was released [38-40].

The Oxford team had to make considerable efforts (initially sometimes difficult) to raise funds. Their efforts did, however, result in them receiving support from the NIHR (including Oxford Biomedical Research Centre, the CRN and through using some of the aid budget research funding for vaccine development), the MRC, the Centre for Epidemic Preparedness Innovation (CEPI), and many others [38,39,41]. CEPI, launched in 2017, used resources received from governments and philanthropic organisations to fund the development of vaccines [12,38], and in March 2020 the UK Government's COVID-19 vaccine donation of GBP210m to CEPI was the *"largest single contribution by any country to date"* [42].

The team also liaised (globally) with researchers and AstraZeneca and rapidly progressed ethical approval and other aspects of the trials conducted widely in the NHS, and eventually overseas [38-41,43-45]. A key animal study was successfully conducted in the NIH lab of their collaborators and published in *Nature i*n July, 2020 [44], by which time the human trials were well underway. In the UK, the speed of the trials was increased by various aspects of the mobilisation of the NIHR CRN, as listed above, including the vaccine's prioritisation as one of the six initial Urgent Public Health studies [7]. The findings from the UK phase 2/3 trial were published in the *Lancet* in November 2020 [45], with the combined findings from the trials in the UK, Brazil and South Africa published also in the *Lancet* a month later [41].

A previous analysis about how processes of vaccine/drug development might be accelerated had proposed approaches such as using more resources and conducting the usual phases of development in parallel overlapping ways where possible [46]. The rapid development of the Oxford/AstraZeneca and other vaccines provided evidence that encouraged such lessons to be further elaborated [43,47,48].

Surveillance

During March 2020 another rapid collaboration driven initially by researchers led by Sharon Peacock, with the support of the UK Government's Chief Scientific Adviser Patrick Vallance, successfully created the COVID-19 Genomics UK Consortium (COG-UK). With funding from the UKRI/MRC, NIHR and Wellcome it built on existing strengths in areas such as pathogen genomics and the network of specialist academic facilities working with public health agencies and the NHS [12,49,50]. Peacock highlighted the importance of MRC pre-pandemic funding that had established a readily accessible cloud bioinformatic infrastructure. She also *"recognised the importance of an open science culture"* [49], and described how the consortium members posted details of their work, made manuscripts available as preprints and published in open access formats. In a paper published online in July 2021 a team from the US noted, *"With respect to genomic surveillance of COVID-19, the vast majority of the most reliable data derive from the COVID-19 Genomics UK(COG-UK) collaborations between academicians and public health authorities"* [51].

In October 2021 the UK Health Security Agency (formerly one part of Public Health England) provided an overview of various national surveillance programmes in the UK funded by the health department and delivered by *"research, academic and commercial partners"* and others [52]. The most high-profile ones developed by UK scientists included the Real-time Assessment of Community Transmission (REACT) study based at Imperial College London and the Zoe COVID-19 app from King's College London. Both were highly regarded for the surveillance studies they conducted [53,54].

As the pandemic continued, attempts to understand the nature of the disproportionate impact of COVID-19 on certain ethnic populations highlighted some of the detailed issues that still needed to be explored: *"Prioritising linkage between health, social and employment data will be essential in building a complete picture of ethnic differences in COVID-19 risk and outcomes"* [55].

Section 2: *Examples of achievements in promoting evidence use in products, policymaking and practice; examples (and lessons) about links to existing strengths*

Gilbert and the Oxford/ AstraZeneca team started working early in a productive, iterative manner with the UK regulator, the Medicines and Healthcare products Regulatory Agency (MHRA), to ensure all the usual approval steps were completed, but much more rapidly than usual, resulting in an effective vaccine being approved (as were Pfizer/BioNTech, then Moderna) [38,41,43]. Green explained they started talking to the MHRA early: "*we kept talking to them all the way through, and they reviewed all of our data – over 500,000 pages of it – on a rolling basis...looked at it just as carefully as they always do. But they started sooner, and put more people on it"* [38].

Under the Vaccine Task Force established by the Government and led by Kate Bingham [12], the vaccine advance purchase went well [47], as did the vaccine rollout generally. The UK Parliament's enquiry, *Coronavirus: lessons learned to date*, took a wide range of evidence on many issues. Published in September 2021, it claimed the UK's vaccination programme *"encompassing discovery, purchase and full vaccination of over 80% of the adult population by September 2021...has been one of the most effective initiatives in the history of UK science and public administration"* [56]. (However, as Table 1 of the main paper shows, by the end of 2021 the UK vaccination rate as a percentage of the total population was lagging that of various countries that had started vaccinating after the UK [57]).

While the genomics consortium was a research consortium it aimed to provide data and analysis tools to inform public health actions and policy decisions [49]. According to one of many papers the consortium's real-time whole genome sequencing proved very valuable in controlling outbreaks in hospital settings, both in identifying high risk areas and also *"validating existing control measures in other units, maintaining clinical service overall"* [50].

Building on its well-established role alongside the NIHR of producing clinical guidance for the NHS, the National Institute for Health and Care Excellence effectively accelerated its processes during the pandemic. By December 2021 it had produced guidance on 19 COVID-19 topics. Some were frequently updated as new evidence emerged [58]. As one example, on Vitamin D, the rapid but thorough review of over 200 pages repeated Government recommendations for taking it, but not, on existing knowledge, as something to prevent or treat COVID-19 other than in clinical trials, which it recommended should occur [59].

The rapid uptake of corticosteroids in clinical practice in the NHS [5,13] partly reflected the organisational structure of the NHS and almost instant use recommendation from Chief Medical Officers across the UK nations [60]. Rapid uptake was also enhanced by the build-up of research capacity embedded into the NHS through the CRN and other aspects of the NIHR research infrastructure described in the background of the main paper [30,31,61]. In addition to the benefits the CRN provided to the production of research, the experience from the pandemic might reinforce lessons from studies conducted earlier for the NIHR indicating research active healthcare organisations were likely to have better outcomes, partly because the medical academics knowing about and trusting the research findings, were more likely to apply them [62,63].

Trish Greenhalgh from the Oxford COVID-19 Evidence Service was reported as having tweeted on 21 March 2020 that it should be recognised how rapidly some COVID-19 research evidence was influencing a range of policy and practice: *"This week, I've seen observational studies and rapid reviews done in days, which have changed policy in minutes and practice in hours"* [64].

An initial detailed estimate of the possible health gains from the use of dexamethasone was made by members of the RECOVERY team and others for the period from July-December 2020, but the margins were wide, especially for the global picture [19]. Building on these figures, the NHS estimated that by March 2021 the use of dexamethasone had saved 22,000 lives in the UK, and possibly a million worldwide [65]. Public Health England estimated 27,200 deaths had been averted in England by the COVID-19 vaccination programme by 19 June 2021 [66], and a study of those aged 60+ across 33 European countries estimated the vaccines averted 470,000 deaths by November 2021 [67].

Studies also began to consider how to assess the value of the impact of rapid COVID-19 research, especially in the context of possible challenges to the science budget in the light of the damage to government finances caused by the pandemic [3]. Such work also identified new areas to consider in the circumstances of a pandemic. First, the benefits of having capacity in the Biomedical Research Centres that could be rapidly deployed to address the crisis [3] - the role of such capacity was also considered in a later paper [68]. Second, it proposed that any resulting contribution to accelerating research that was used to reduce the need for economically damaging pandemic counter-measures, would provide a high rate of return [3]. Another UK study involved a detailed analysis of the impact of having a largely NIHR-coordinated research infrastructure embedded in a major London Hospital

Trust. This could facilitate the rapid redeployment of research staff – some to provide extra clinical staff in the COVID-19 crisis, and some to contribute to the COVID-19 research that made such rapid progress in the UK [69]. This paper, therefore, highlighted the degree of effort and reorganisation that was required to achieve the benefits from having the research infrastructure that could respond flexibly to the pandemic.

Section 3: *Examples of challenges in: 1. production of evidence; 2. use of evidence; and 3. sustaining the research system; examples of lessons to address challenges*

Challenges in production of evidence

The speed with which RECOVERY was established was admired, but there were some criticisms such as about the dosage for some drugs, though, as noted, expert advice was followed [5,70]. A much smaller percentage of admitted patients were recruited at some NHS trusts than others. Concerns about this led Darzi et al., to call for research to become part of the clinical pathway for all COVID-19 patients [71], something they, and others, also said should be a routine approach across the NHS, e.g., by using platform trials [10].

The acceleration of urgent COVID-19 research reduced the average time from 62 to five days for Health Research Authority approval, but initially there was a big drop in PPI [72]. Therefore, the Authority established a matching service connecting research teams with existing PPI groups. Within six months *"the level of public involvement in Covid-19 research recovered to - and exceeded – the normal level"* [72]. Despite challenges from the shift to online meetings, some were positive about the benefits in terms of inclusivity [4,72].

Challenges in use of evidence

The Scientific Advisory Group for Emergencies (SAGE) advises the UK government in emergencies. Both were strongly criticised by the Parliamentary report for the high number of deaths resulting from the slowness of the decision to lockdown in the first wave. Even when SAGE advised strong measures on 16 March 2020, the Government took a further week to act [56]. Several reasons were suggested for what appeared to be a type of initial "group think" that some claimed was a belief in "herd immunity" against a pandemic wrongly assumed to be flu-like. First, insufficient attention was given to international evidence and those who relayed it, including experts from the UK medical academic world such as the editor of the *Lancet*, Richard Horton. This might have resulted from a lack of international members on SAGE, and a political culture that promoted British exceptionalism and had weakened trust in evidence [56,73,74]. Second, SAGE members were perhaps too cautious because introducing a lockdown seemed to be off the Government's agenda [74-76].

Many scientists were angry that the Government initially seemed to be slower than others to introduce science-informed NPIs such as lockdowns, and some, including a former Chief Scientific Advisor, established their own *"Independent SAGE"* in an attempt to increase transparent and evidence-informed advice [64,73]. One area of particular concern was the disproportionate impact of COVID-19 on certain ethnic minority populations [55,56]. Another early area of extreme concern was the limited testing in the UK and the consequently large number of deaths in care homes [56]. Jeremy Farrar noted that by 11 March, Public Health England was conducting about 10,000 tests per week, while Germany was doing 500,000. By April, he said, it was obvious the virus was spreading in

institutions but without greater testing it was difficult to gauge exactly what was going on. He commented: *"No matter how much SAGE advised that such testing was central to any strategy, advisers had no operational power or oversight to make it happen"* [12].

Responsibility for policies that ran the risk of high death rates in the subsequent waves seemed clear. Farrar explained in detail that the advice from a SAGE meeting of 21 September 2020, and then an accompanying paper, *"was unambiguous: 'A package of interventions will be needed to reverse this exponential rise in cases'.. ..'not acting now to reduce cases will result in a very large epidemic with catastrophic consequences in terms of direct COVID related deaths'"* [12]. The paper also made clear that this second wave would disproportionately hit the frailest in society and those on lower income and from Black, Asian and minority communities. It was clearly spelt out that stricter restrictions would mean fewer deaths. At that stage PM Johnson refused to take the action he eventually had to take weeks later.

In relation to this failure to act at the appropriate time, Farrar wrote: *"I respect the mantra that scientists advise and ministers must decide, but ministers were clearly overriding SAGE advice, often while claiming to follow it...I began to question the point of giving advice to a body that chose not use it"* [12]. He reflected on *"the delays that preceded the second lockdown, despite the wealth of data pointing to imminent disaster"* then also stated: *"Many of the UK's Covid-19 deaths happened in January, February and March of 2021; they were avoidable. The political decisions made, or not made, in the second half of 2020 were unforgivable"*
[12].

Policy analysts from New Zealand with long-experience of the UK system described the importance of mobilising expertise to deliberate on public policies as PM Ardern had successfully done, but then also commented on a broader move by recent UK governments away from a previous pattern of deliberation with expertise. They claimed, *"the British government's stumbling (at least from a New Zealand perspective) response to COVID-19, hard on the heels of the policy-making 'omnishambles' of the UK's departure from the EU, is nothing unusual"* [77].

Several analyses of lessons from the COVID-19 research response, including from the UK, considered the challenges in producing evidence to inform policies about NPIs such as face masks where it was extremely difficult to conduct standard RCTs relevant to the issues faced in the pandemic [32,64,73,78]. Some pointed to the frustration of researchers such as Greenhalgh et al., who in an article in the *BMJ* in April 2020 had drawn on the evidence that was available on masks and called for support for mask mandates based on application of the precautionary principle [78], only to find policymakers in the UK initially seemed reluctant to act in the absence of the gold-standard RCT evidence [32,64].

Farrar and others also asked why countries ranked 1st and 2nd on the 2019 GHS Index (US and UK) [79] suffered high death rates. On the specific part of the Index related to preparing for a pandemic, the UK had the highest score. According to one researcher looking at the high scores but poor performance by the UK and US: *"we had everything – except leadership"* [80]. One of the GHS Index team was quoted as saying: *"Even though the US and UK had the best environments in terms of plans in place and thinking about what they would need in terms of capacity...when it came to the moment that everyone had been preparing for, the decision-making really hampered the actual ability of the country to respond"* [80].

Leadership and evidence-based policies were required from the start. Horton told the UK Parliamentary inquiry that his journal, the *Lancet,* had published three papers in late January 2020 describing the alarming nature of the new disease, and that February had been the opportunity for the UK to really prepare for this disease. But, he stated, the UK "*missed that opportunity. We could have used the month of February, based on what we knew in January"* [56]. Similarly, several WHO reports throughout February 2020 described public health steps for countries to follow, including, according to the Parliamentary report, advice on 24 February 2020 that countries should "*Immediately activate the highest level of national Response Management protocols to ensure the all-of-government and all-of-society approach needed to contain COVID-19 with non-pharmaceutical public health measures"* [56]. Farrar reported the claims, however, that PM Johnson had not been paying attention to the pandemic in the vital early weeks [12] - this period when action was required was well before the PM first tested positive for COVID-19 on 27 March 2020, and before the eventual announcement on 23 March 2020 by the UK Government of the of the (delayed) first lockdown.

The UK's position on the GHS Index published in December 2021 fell to seventh compared to second in 2019 [81].

The cross-country analysis by Tworek et al., of the communications used by authorities in nine countries with at least a reasonably effective response to COVID-19 contrasted them *"with democracies that struggled to communicate around Covid-19, particularly the United Kingdom and the United States"* [82]. Journalists commenting on this noted that whereas some governments have been praised for the science-driven way that they have communicated about the pandemic, others *"most notably the U.S. and the U.K., have been hit with criticism for public health messages that are confusing or not based in science"* [83].

Challenges in sustaining the research system

Reductions in the funds of medical research charities [84] created major challenges for many researchers, as did the tight central controls, and delays, even on many COVID-19 projects other than those badged as Urgent Public Health studies [4]. Gradually more trials could be set up on COVID-19 in the NIHR CRN, and continued, or set up, on other topics, with about 80% of previously paused studies reopened by March 2021 [2].

A December 2021 report noted continued challenges but more progress in managed recovery of the wider clinical research portfolio. It described moves, partly informed by pandemic lessons, to *"drive value creation for industry and patients, delivered through faster, cheaper, better-quality... research embedded across the NHS as a core part of effective patient care"* [85].

Summary

Strengths of the UK's HRS, including the research network embedded into the healthcare system, in line with the 2006 comprehensive NIHR strategy, resulted in major advances in knowledge production and the Oxford/AstraZeneca vaccine. Leaders of the UK's HRS ensured a coordinated approach by applying a strict prioritisation process in which initially only a small number of key trials were allowed to use the resources of the NIHR in the public healthcare system. Priorities included RECOVERY and REMAP-CAP, the major adaptive platform trials. The strengths of the CRN capacity in

the unified health care system were further developed with measures including accelerated ethical approval and enhanced data access. This enabled accelerated research progress with RECOVERY producing a series of rapidly published papers with key findings, including dexamethasone as the first therapy proven to reduce COVID-19 deaths. The UK system also resulted in much less waste than was reported elsewhere. The long-term vaccine development capacity at Oxford University led by Sarah Gilbert and colleagues provided a platform that facilitated unprecedentedly rapid vaccine development, and again highly cited publications. Similarly, existing capacity in the academic system was developed to create a world-leading genomic surveillance system. While political leaders did not openly dismiss the science in the way that occurred in Brazil and the US, at key times they appeared to fail to use and communicate the evidence effectively. Therefore, policies from PM Johnson and his Government that disregarded the scientific evidence are held to be responsible, at least in part, for a much higher death rate than in comparable countries such as Germany.

References

1. National Institute for Health Research (NIHR). NIHR and UKRI launch £20 million funding call for novel coronavirus research. NIHR, 4 Feb 2020. https://www.nihr.ac.uk/news/nihr-and-ukri-launch-20-million-funding-call-for-novel-coronavirus-research/23942. Accessed 17 Jan 2022.
2. NIHR. A record high: participation in NIHR clinical research network studies doubles in pandemic year. NIHR, 6 July 2021. News: A record high: Participation in NIHR Clinical Research Network studies doubles in pandemic year | NIHR. Accessed 17 Jan 2022.
3. Roope LSJ, Candio P, Kiparoglou V, McShane H, Duch R, Clarke PM. Lessons from the pandemic on the value of research infrastructure. Health Res Policy Sys. 2021; doi: https://doi.org/10.1186/s12961-021-00704-2.
4. Wyatt D, Faulkner-Gurstein R, Cowan H, Wolfe CDA. Impacts of COVID-19 on clinical research in the UK: a multi-method qualitative case study. PLoS ONE. 2021; doi: https://doi.org/10.1371/journal.pone.0256871.
5. Wise J, Coombes R. Covid-19: The inside story of the RECOVERY trial. BMJ. 2020; doi: https://doi.org/10.1136/bmj.m2670.
6. NIHR. News: DHSC issues guidance on the impact of COVID-19 on research funded or supported by NIHR. NIHR, 16 March 2020. News: DHSC issues guidance on the impact of COVID-19 on research funded or supported by NIHR | NIHR. Accessed 17 Jan 2022.
7. NIHR. COVID-19 vaccine and therapies research boosted by six new projects in £10.5 million rapid funding round. NIHR, 23 March 2020. News: COVID-19 vaccine and therapies research boosted by six new projects in £10.5 million rapid funding round | NIHR. Accessed 17 Jan 2022.
8. NIHR. Case study: REMAP-CAP tests treatments for COVID-19. NIHR, Original version: 18 Sept 2020, updated regularly. https://www.nihr.ac.uk/case-studies/remap-cap-tests-treatments-for-covid-19/25044. Accessed 17 Jan 2022.
9. Horby P, Mullard A. RECOVERY 1 year on: a rare success in the COVID-19 clinical trial landscape. Nature Reviews Drug Discovery. 2021; doi: https://doi.org/10.1038/d41573-021-00068-w.
10. Pessoa-Amorim G, Campbell M, Fletcher L, Horby P, Landray M, Mafham M, et al. Making trials part of good clinical care: lessons from the RECOVERY trial. Future Healthc J. 2021; doi: https://doi.org/10.7861/fhj.2021-0083
11. Goossens H, Derde l, Horby P, Bonten M. The European clinical research response to optimise treatment of patients with COVID-19: lessons learned, future perspective, and recommendations. Lancet Infect Dis. 2021; doi: https://doi.org/10.1016/S1473-3099(21)00705-2.
12. Farrar J, Ahuja A. Spike - The virus vs the people. The inside story. London: Profile Books; 2021.

13. Kupferschmidt K. One U.K. trial is transforming COVID-19 treatment. Why haven't others delivered more results? Science. 2020; doi: 10.1126/science.abd6417.
14. Mather N. How we accelerated clinical trials in the age of COVID-19. Nature. 2020;584:326.
15. RECOVERY. Low-cost dexamethasone reduces death by up to one third in hospitalised patients with severe respiratory complications of COVID-19. Oxford University News Release; University of Oxford; 16 June 2020. recovery_dexamethasone_statement_160620_v2final.pdf (recoverytrial.net). Accessed 12 Jan 2022.
16. RECOVERY Collaborative Group. Dexamethasone in hospitalized patients with Covid-19. NEJM. 2021; doi: 10.1056/NEJMoa2021436.
17. The WHO Rapid Evidence Appraisal for COVID-19 Therapies (REACT) Working Group. Association between administration of systemic corticosteroids and mortality among critically ill patients with COVID-19. A meta-analysis. JAMA. 2020; doi: https://dx.doi.org/10.1001%2Fjama.2020.17023
18. Lamontagne F, Agoritsas T, Macdonald H, Leo YS, Diaz J, Agarwal A, et al. A living WHO guideline on drugs for covid-19. BMJ. 2020; doi: http://dx.doi.org/10.1136/bmj.m3379.
19. Águas R, Mahdi A, Shretta R, Horby P, Landray M, White L, et al. Potential health and economic impacts of dexamethasone treatment for patients with COVID-19. Nat Commun. 2021; doi: https://doi.org/10.1038/s41467-021-21134-2.
20. RECOVERY Collaborative Group. Effect of hydroxychloroquine in hospitalized patients with Covid-19. N. Engl. J. Med. 2020; doi: https://doi.org/10.1056/NEJMoa2022926.
21. Axfors C, Schmitt AM, Janiaud P, van't Hooft J, Abd-E-Salam S, Abdo EF, et al. Mortality outcomes with hydroxychloroquine and chloroquine in COVID-19 from an international collaborative meta-analysis of randomized trials. Nat Commun. 2021; doi: https://doi.org/10.1038/s41467-021-22446-z.
22. RECOVERY Collaborative Group. Tocilizumab in patients admitted to hospital with COVID-19 (RECOVERY): a randomised, controlled, open-label, platform trial. Lancet. 2021; doi: 10.1016/S0140-6736(21)00676-0.
23. The REMAP-CAP Investigators. Interleukin-6 receptor antagonists in critically ill patients with Covid-19. N. Engl. J. Med. 2021;384:1491-1502.
24. Agarwal A, Rochwerg B, Lamontagne F, Siemieniuk RAC, Agoritsas T, Askie L, et al. A living WHO guideline on drugs for covid-19. BMJ.2020; doi: http://dx.doi.org/10.1136/bmj.m3379.
25. Lane HC, Fauci AS. Research in the context of a pandemic. NEJM. 2020; doi: 10.1056/NEJMe2024638.
26. Couzin-Frankel J. 'We've got to be able to move more quickly.' The pandemic reality of COVID-19 clinical trials. Science. June 16, 2020. https://www.sciencemag.org/news/2020/06/we-ve-got-be-able-move-more-quickly-pandemic-reality-covid-19-clinical-trials. Accessed 10 Jan 2022.
27. Bowen AC, Tong SYC, Davis JS. Australia needs a prioritised national research strategy for clinical trials in a pandemic: lessons learned from COVID-19. Med J Aust. 2021; doi: https://doi.org/10.5694/mja2.51143.
28. Lamontagne F, Rowan KM, Guyatt G. Integrating research into clinical practice: challenges and solutions for Canada. CMAJ 2021; doi: https://doi.org/10.1503/cmaj.202397.
29. Emanuel EJ, Zhang C, Diana A. Where Is America's groundbreaking Covid-19 research? The U.S. could learn a lot from Britain. New York Times, 1 Sept, 2020. https://www.nytimes.com/2020/09/01/opinion/coronavirus-clinical-research.html?smid=em-share. Accessed 7 Sept 2020.
30. Davies S. Ten years of the NIHR: achievements and challenges for the next decade (23rd Annual Lecture, Royal College of Physicians). London: Office of Health Economics; 2017. https://www.ohe.org/sites/default/files/10%20years%20of%20NIHR%20V8.pdf. Accessed 28 Nov 2021.
31. Hanney S, Kanya L, Pokhrel S, Jones T, Boaz A. What is the evidence on policies, interventions and tools for establishing and/or strengthening national health research systems and

their effectiveness? Health Evidence Network (HEN) synthesis report 69. Copenhagen: WHO Regional Office for Europe; 2020. https://apps.who.int/iris/bitstream/handle/10665/331703/9789289054942-eng.pdf.
32. Pearson H. How COVID broke the evidence pipeline. The pandemic stress-tested the way the world produces evidence — and revealed all the flaws. Nature. 2021; doi: https://doi.org/10.1038/d41586-021-01246-x.
33. Seidler AL, Aberoumand M, Williams JG, Tan A, Hunter KE, Webster A. The landscape of COVID-19 trials in Australia. The research response in Australia has been rapid, but better coordination is imperative. Med J Aust . 2021; doi: https://doi.org/10.5694/mja2.51148
34. NIHR. NIHR's response to COVID-19. NIHR, ongoing. NIHR's response to COVID-19 | NIHR. Accessed 10 Nov 2021.
35. NIHR. Researching the long-term impact. Researching the long-term impact | NIHR. Accessed 17 Jan 2022.
36. Lazarus R, Baos S, Cappel-Porter H, Carson-Stevens A, Clout M, Culliford L, et al. Safety and immunogenicity of concomitant administration of COVID-19 vaccines (ChAdOx1 or BNT162b2) with seasonal influenza vaccines in adults in the UK (ComFluCOV): a multicentre, randomised, controlled, phase 4 trial. Lancet. 2021; doi: 10.1016/S0140-6736(21)02329-1.
37. Atkinson P, Gobat N, Lant S. Understanding the policy dynamics of COVID-19 in the UK: early findings from interviews with policy makers and health care professionals. Soc Sci Med. 2020; doi: https://doi.org/10.1016/j.socscimed.2020.113423.
38. Gilbert S, Green C. Vaxxers. The inside story of the Oxford AstraZeneca vaccine and the race against the virus. London: Hodder and Stoughton; 2021.
39. Lane R. Sarah Gilbert: carving a path towards a COVID-19 vaccine. Lancet. 2020; doi: https://doi.org/10.1016/S0140-6736(20)30796-0
40. Gallagher J. Oxford vaccine: How did they make it so quickly? BBC, 23 Nov 2020. Oxford vaccine: How did they make it so quickly? - BBC News. Accessed 23 Nov 2020.
41. Voysey M, Clements SAC, Madhi SA, Weckx LY, Folegatti PM, Aley PK, et al. Safety and efficacy of the ChAdOx1 nCoV-19 vaccine (AZD1222) against SARS-CoV-2: an interim analysis of four randomised controlled trials in Brazil, South Africa, and the UK. Lancet. 2021; doi: 10.1016/S0140-6736(20)32661-1.
42. Prime Minister's Office. PM announces record funding to find a coronavirus vaccine. 26 March 2020. PM announces record funding to find a coronavirus vaccine - GOV.UK (www.gov.uk) Accessed 4 May 2022.
43. Hanney SR, Wooding S, Sussex J, Grant J. From COVID-19 research to vaccine application: why might it take 17 months not 17 years and what are the wider lessons? Health Res Policy Sys. 2020; doi: https://doi.org/10.1186/s12961-020-00571-3.
44. van Doremalen N, Lambe T, Spencer A, Belij-Rammerstorfer S, Purushotham JN, Port, JR, et al. ChAdOx1 nCoV-19 vaccine prevents SARS-CoV-2 pneumonia in rhesus macaques. Nature. 2020; doi: https://doi.org/10.1038/s41586-020-2608-y.
45. Ramasamy M, Minassian AM, Ewer KJ, Flaxman AL, Folegatti PM, Owens DR, et al. Safety and immunogenicity of ChAdOx1 nCoV-19 (AZD1222) vaccine administered in a primeboost regimen in older adults (COV002): a phase 2/3 single blind, randomised controlled trial. Lancet. 2020; doi: https://doi.org/10·1016/S0140–6736(20)32466–1.
46. Hanney SR, Castle-Clarke S, Grant J, Guthrie S, Henshall C, Mestre-Ferrandiz J, et al. How long does biomedical research take? Studying the time taken between biomedical and health research and its translation into products, policy, and practice. Health Res Policy Sys. 2015; doi: https://doi.org/10.1186/1478-4505-13-1.
47. Winch GM, Cao D, Maytorena-Sanchez E, Pinto J, Sergeeva N, Zhang, S. Operation Warp Speed: Projects responding to the COVID-19 pandemic. Project Leadership and Society. 2021; doi: https://doi.org/10.1016/j.plas.2021.100019.

48. Lurie N, Saville M, Hatchett R, Halton J. Developing covid-19 vaccines at pandemic speed. NEJM. 2020; doi: https://doi.org/10.1056/NEJMp2005630.
49. COVID-19 Genomics UK Consortium. History of COG-UK. History of COG-UK | COVID-19 Genomics UK Consortium (cogconsortium.uk). Accessed 30 Dec 2021.
50. Francis RV, Billam H, Clarke M, Yates C, Tsoleridis T, Berry L, et al. The impact of real-time whole-genome sequencing in controlling healthcare-associated SARS-CoV-2 outbreaks. J Infectious Diseases. 2022;225:10-8. (Published online: Sept 2021). https://doi.org/10.1093/infdis/jiab483
51. Robishaw JD, Alter SM, Solano JJ, Shih RD, DeMets DL, Maki DG, et al. Genomic surveillance to combat COVID-19: challenges and opportunities. Lancet Microbe. 2021 Sep;2(9):e481-e484. doi: 10.1016/S2666-5247(21)00121-X
52. UK Health Security Agency. COVID-19 surveillance and immunity studies. UK Health Security Agency. 13 Oct 2021 (Updated 31 Jan 2022). COVID-19 surveillance and immunity studies - GOV.UK (www.gov.uk) Accessed 20 May 2022.
53. Elliott J, Whitaker M, Bodinier B, Eales O, Riley S, Ward H, et al. Predictive symptoms for COVID-19 in the community: REACT-1 study of over 1 million people. PLoS Med, 2021;8(9): e1003777. https://doi.org/10.1371/journal.pmed.1003777
54. Menni C, Valdes AM, Freidin MB, Sudre CH, Nguyen LH, Drew DA, *et al.* Real-time tracking of self-reported symptoms to predict potential COVID-19. Nat Med, 2020;26:1037–40. https://doi.org/10.1038/s41591-020-0916-2
55. Mathur R, Rentsch CT, Morton CE, Hulme WJ, Schultze A, MacKenna B, et al. Ethnic differences in SARS-CoV-2 infection and COVID-19-related hospitalisation, intensive care unit admission, and death in 17 million adults in England: an observational cohort study using the OpenSAFELY platform. Lancet 2021;397:1711–24. https://doi.org/10.1016/S0140-6736(21)00634-6
56. Health and Social Care, and Science and Technology Committees. Coronavirus: lessons learned to date. House of Commons. HC 92. 21 Sept 2021. Coronavirus: lessons learnt (parliament.uk). Accessed 18 Jan 2022.
57. Mathieu, E., Ritchie, H., Ortiz-Ospina, E. et al. A global database of COVID-19 vaccinations. Nat Hum Behav. 2021;5:947–953. Web update for 31 Dec 2021: Coronavirus (COVID-19) Vaccinations - Our World in Data Accessed 10 Aug 2022.
58. National Institute for Health and Care Excellence (NICE). COVID-19. All NICE products on COVID-19. Includes any guidance and advice. COVID-19 | Topic | NICE. Accessed 10 Nov 2021.
59. NICE. COVID-19 rapid guideline: vitamin D. NG187. 17 Dec 2020. Overview | COVID-19 rapid guideline: vitamin D | Guidance | NICE. Accessed 29 Dec 2021.
60. Burki TK. Completion of clinical trials in light of COVID-19. Lancet Respir Med. 2020; doi: https://doi.org/10.1016/S2213-2600(20)30460-4.
61. Morgan Jones M, Kamenetzky A, Manville C, Ghiga I, MacLure C, Harte E, et al. The National Institute for Health at 10 years: an impact synthesis. Summary report. Santa Monica: RAND Corporation: 2016. http://www.rand.org/content/dam/rand/pubs/research_reports/RR1500/RR1574/RAND_RR1574.summary.pdf. Accessed 19 Dec 2021.
62. Boaz A, Hanney S, Jones T, Soper B. Does the engagement of clinicians and organisations in research improve healthcare performance: a three-stage review. BMJ Open 2015; doi:10.1136/bmjopen-2015-009415.
63. Ozdemir BA, Karthikesalingam A, Sinha S, Poloniecki JD, Hinchcliffe RJ, Thompson MM, et al. Research activity and the association with mortality. PLoS ONE 2015; doi: https://doi.org/10.1371/journal.pone.0118253.
64. Lancaster K, Rhodes T, Rosengarten M. Making evidence and policy in public health emergencies: lessons from COVID-19 for adaptive evidence-making and intervention. Evidence & Policy. 2020; doi: https://doi.org/10.1332/174426420X15913559981103.

65. NHS England. COVID treatment developed in the NHS saves a million lives. News, 23 March 2021. NHS England » COVID treatment developed in the NHS saves a million lives. Accessed 3 Jan 2022.
66. Public Health England (PHE). Direct and indirect impact of the vaccination programme on COVID-19 infections and mortality. PHE, 28 June 2021. COVID-19 vaccine surveillance report - week 25 (publishing.service.gov.uk). Accessed 18 Jan 2022.
67. Meslé MMI, Brown J, Mook P, Hagen J, Pastore R, Bundle N, et al. Estimated number of deaths directly averted in people 60 years and older as a result of COVID-19 vaccination in the WHO European Region, December 2020 to November 2021. Euro Surveill. 2021; doi: https://doi.org/10.2807/1560-7917.ES.2021.26.47.2101021
68. Henderson LR, McShane H, Kiparoglou V. Rapid research response to the COVID-19 pandemic: perspectives from a National Institute for Health Biomedical Research Centre. Health Res Policy Sys. 2022;20:24. https://doi.org/10.1186/s12961-022-00827-0
69. Faulkner-Gurstein R, Wyatt D, Cowan H, Hare N, Harris C, Wolfe C. The organization and impacts of clinical research delivery workforce redeployment during the COVID-19 pandemic: a qualitative case study of one research-intensive acute hospital trust. Health Res Policy Sys. 2022;20:68. https://doi.org/10.1186/s12961-022-00876-5
70. White NJ, Watson JA, Hoglund RM, Chan XHS, Cheah PY, Tarning J. COVID-19 prevention and treatment: a critical analysis of chloroquine and hydroxychloroquine clinical pharmacology. PLoS Med. 2020; doi: https://doi.org/10.1371/journal.pmed.1003252
71. Darzi A, Goddard A, Henderson K. Increasing recruitment into covid-19 trials. BMJ 2021; doi: https://doi.org/10.1136/bmj.n235.
72. Health Research Agency (HRA). Public involvement in a pandemic: lessons from the UK COVID-19 public involvement matching service. NHS HRA, last updated 27 Nov 2021. Public involvement in a pandemic: lessons from the UK COVID-19 public involvement matching service - Health Research Authority (hra.nhs.uk). Accessed 18 Jan 2021.
73. Van Schalkwyk MCI, McKee M. Research into policy: lessons from the COVID-19 pandemic. Eur J Public Health. 2021; doi: https://doi.org/10.1093/eurpub/ckab155.
74. Ballo R, Pearce W, Stilgoe J, Wilsdon J. United Kingdom. In: Jasanoff S, Hilgartner SJ, Hurlbut JB, Ozgode O, Rayzberg M. Comparative Covid response: crisis, knowledge, politics. Interim report. 12 Jan 2021. https://compcore.cornell.edu/wp-content/uploads/2021/03/Comparative-Covid-Response_Crisis-Knowledge-Politics_Interim-Report.pdf. Accessed 12 Jan 2022.
75. Ball P. Should scientists run the country? Guardian, 27 Sept 2021 Should scientists run the country? (msn.com) Accessed 18 July 2022.
76. Grey S, MacAskill A. Special Report: Johnson listened to his scientists about coronavirus - but they were slow to sound the alarm. Reuters, 7 April 2020. Special Report: Johnson listened to his scientists about coronavirus - but they were slow to sound the alarm | Reuters. Accessed 30 Dec 2021.
77. Mazey S, Richardson J. Lesson-Drawing from New Zealand and Covid-19: the need for anticipatory policy making. The Political Quarterly. 2020; doi: http://dx.doi.org/10.1111/1467-923X.12893.
78. Greenhalgh T, Schmid MB, Czypionka T, Bassler D, Gruer L. Face masks for the public during the covid-19 crisis. BMJ. 2020 Apr 9;369:m1435. doi: 10.1136/bmj.m1435
79. Global Health Security Index (2019). Nuclear Threat Initiative (NTI) and John Hopkins. Oct 2019. https://www.ghsindex.org/wp-content/uploads/2021/11/2019-Global-Health-Security-Index.pdf. Accessed 8 Dec 2021.
80. York C. The UK And US Were Ranked Top For Pandemic Preparedness. What Went Wrong? HuffPost UK. 27 Jan 2021 (updated). The UK And US Were Ranked Top For Pandemic Preparedness. What Went Wrong? | HuffPost UK News (huffingtonpost.co.uk) Accessed 4 May 2022.
81. Global Health Security Index (On-going). About. About - GHS Index. Accessed 6 Nov 2021.

82. Tworek H, Beacock I, Ojo E. Democratic Health Communications during Covid-19: A RAPID Response. Vancouver: UBC Centre for the Study of Democratic Institutions, September 2020. Democratic-Health-Communication-during-Covid_FINAL.pdf (ubc.ca) Accessed 4 May 2022.
83. Robbins R, Garde D, Feuerstein A. An expert's take on what the U.S., U.K did wrong in Covid-19 communications — and what others did right. STAT. 18 Sept 2020. What the U.S. did wrong on Covid-19 — and what others did right (statnews.com) Accessed 4 May 2020.
84. Griffiths C, Mitchell M, Burnard A. More support needed for UK charity-funded medical research. The Lancet, 2020; doi https://doi.org/10.1016/S0140-6736(20)32397-7.
85. Department of Health and Social Care (DHSC). Update from the UK Clinical Research Recovery, Resilience & Growth Programme. DHSC; 2021. Update from the RRG Programme - Final.pdf - Google Drive. Accessed 19 Jan 2022.

United States

Section 1: *Examples of achievements in production of primary and secondary research to inform development of COVID therapies, vaccines, national/local organisational responses; examples (and lessons) about links to existing strengths in the health research system*

The US had the highest funding levels for COVID-19 research, and the most projects and publications [1,2].

<u>*Vaccines*</u>

Parts of the health research system had long researched the basic science around the potential of using mRNA. The many researchers included: Katalin Karikó and Drew Weissman at the University of Pennsylvania whose work showed the importance of modifying mRNA; researchers linked to the NIH; and those around the biotech company Moderna whose name came from combining modified and RNA [3-5]. As noted in the section on Germany above, some scientists pursued this research because they believed it would prove valuable even when, especially Karikó, they received little encouragement as academic researchers. (Following the success of the mRNA vaccines, Karikó, who later also joined BioNTech, and Weissman began winning an increasing number of major scientific awards [3].)

Researchers at Moderna immediately started developing their COVID-19 vaccine once the sequence of the genome was released in mid-January 2020 and they intensified their work with Anthony Fauci's National Institute of Allergy and Infectious Diseases (NIAID). Moderna was first to start human trials of a COVID-19 vaccine [3-6]. It continued working closely with NIAID through the phases of vaccine development with a series of animal and human trials reported in the *NEJM* [7-10]. The NIH/NIAID was the major funder along with others especially Biomedical Advanced Research and Development Authority (BARDA), and the wording about the early human trial emphasised that while employees of Moderna collaborated on protocol development and participated in weekly protocol team calls, NIAID *"ultimately made all decisions regarding trial design and implementation"* [8]. Other vaccine developers working with NIH included the University of Oxford team for one early animal trial [11,12]. (See UK section above.)

Operation Warp Speed (OWS) was created by the US government in May 2020 to coordinate and work through the efforts of various organisations including the work of the Health and Human Science Department's NIH and BARDA and the Department of Defense with its Defense Health Agency and the Defense Advanced Research Projects Agency (DARPA) programme [13]. With an initial budget of almost USD10 billion, Operation Warp Speed (OWS) was created as *"a public-private partnership to facilitate, at an unprecedented pace, the development, manufacturing, and distribution of COVID-19 countermeasures"* [13]. The joint leadership reflected the enormous range of the considerable resources deployed to undertake the various tasks. The Chief Advisor of OWS, Moncef Slaoui, was a venture capitalist and former senior executive at GlaxoSmithkline where he had led development of five major novel vaccines. The chief operating officer, General Gustave Perna, was in charge of the US Army Materiel Command [13].

OWS aimed to accelerate vaccine progress by laying down a prioritisation plan for contributing to late stage development of at least four COVID-19 vaccines and early stage manufacture of six through agreement to purchase millions of doses [13,14]. Its eventual spend of about USD18 billion contributed to the development of vaccines such as Moderna and Johnson and Johnson, and while Pfizer/ BioNTech worked with it on advance purchases, they did not accept money to develop their mRNA vaccine [14,15].

Of the sites in Pfizer's successful Phase 2/3 trial 85% were in the US [16]. Every opportunity was taken to accelerate processes and undertake various activities in parallel, but generally, considerable emphasis was given to ensuring the usual phases of vaccine development were followed [6,14,17].

Vaccine hesitancy was recognised as a problem, and despite its success, OWS's very name exacerbated hesitancy in some people [15,18]. There are powerful reasons, including *"prior harms and ongoing inequalities"*, why vaccine hesitancy is mediated by experiences of group membership and values in communities such as Black Americans [19]. As one of many examples to better understand vaccine motivations in an attempt to inform vaccine promotion, early research by anthropologists was conducted on volunteers who had participated in vaccine trials [19].

Some large integrated health systems in the US have conducted their own studies, including vaccine-related ones, sometimes using their own data. In addition to a study by Kaiser Permanente Southern California [20] (see next section), these included a study published in the *NEJM* in which the Department of Veterans Affairs used its own data to compare the effectiveness of Moderna and Pfizer/BioNTech vaccines. It found them both to be highly effective, but Moderna slightly more so [21].

Diagnostics

The enormous range of COVID research in the US covered many topics – a large range of projects were funded under the NIH's Rapid Acceleration of Diagnostics (RADx) initiative to speed innovation in development, commercialization and implementation of technologies for COVID-19 testing [22]. This programme was introduced after the disastrous failures of the initial plans for testing in the US in the early months when technical problems were compounded because *"The President spent nearly two months issuing confusing and contradictory signals"* [23]. In late April 2020 the US Congress approved a USD1.5 billion allocation to the NIH to improve testing. The NIH launched the RADx initiative just five days later on 29 April 2020. An NIH team described it in a special report in the *NEJM: "this program represents a dramatic extension of the usual NIH mode of supporting research...it covers the entire life cycle of the target technologies; it is tightly focused on timelines and outcomes"* [24]. It worked with companies and partnered with other government agencies including BARDA and the Department of Defense.

The RADx programme had short and longer term aims and had four components. The RADx Tech component aimed to identify and accelerate the development, scale up and deployment of innovative point of care and home based tests. It leveraged the NIH's long-existing Point-of-Care Technology Research Network [24]. By November 2021 this initiative had a budget of USD666m [25]. The RADx Advanced Technology Platforms element aimed to support the scale up of more advanced technologies intended to rapidly and substantially increase capacity, where a further

element, the RADx Radical initiative, had a longer horizon and was aimed at new, non-traditional approaches [24].

Finally, the fourth component, RADx Underserved Population (RADx-UP) initiative, announced its first funding support in September 2020 for 32 institutions that were awarded USD234m in total. The programme focused on groups disproportionally affected by the pandemic which included African Americans, American Indians/Alaskan Natives, older adults, pregnant women and the homeless and imprisoned. The programme aimed to *"understand COVID-19 testing patterns better among underserved and vulnerable populations; strengthen the data on disparities in infection rates, disease progression and outcomes; and develop strategies to reduce these disparities in COVID-19 testing"* [26]. By November 2021 the budget for RADx-UP was USD512m [25].

Therapies

The clinical research picture was particularly complicated. As early as February 2020 a trial began of Gilead's antiviral treatment remdesivir, which had been developed to treat other viruses and used in trials against Ebola [27]. The NIH's NIAID was primary funder of the trial along with the Department of Defense's Defense Health Program, but it also had some international funding and a small proportion of sites in a few Asian and European countries. Its preliminary findings were reported in the *NEJM* in May 2020, and the final version in October [28]. It suggested remdesivir shortened the time to recovery in patients who were hospitalised with COVID-19, but a reduction in mortality was not statistically significant [28].

Accompanying NIH-funded studies were already underway to evaluate remdesivir's effectiveness in combination with other therapies, including baricitinib, a Janus Kinase inhibitor. This study team was similar to the first. Enrolment started in May 2020. Findings published online in the *NEJM* in December 2020 showed the combined treatment was superior to remdesivir alone, and with fewer serious adverse effects [29].

Meanwhile, in November 2020 WHO's living guideline was updated in the light of the WHO Solidarity platform trial [30-32] and, unlike in the US, made a conditional recommendation against the use of remdesivir because *"there is no evidence based on currently available data that it does improve patient-important outcomes. The panel placed low value on small and uncertain benefits in the presence of the remaining possibility of important harms"* [32].

Progress with clinical research faced challenges. Derek Angus from the University of Pittsburgh had helped establish the original REMAP-CAP platform [33], and led on that platform's paper on its trial of corticosteroids as a therapy for COVID [34]. But there were only a few US institutions represented on the writing committee and, as noted above, the UK provided an increasingly high proportion of the patients for REMAP-CAP trials [35].

In this general context (and see section 3 for more details), the Director of the NIH, Francis Collins, wrote in May 2020 that *"it soon became apparent that much-needed coordination among important constituencies was lacking"* [36]. He described how in April 2020 the NIH had therefore launched the Accelerating COVID-19 Therapeutic Interventions and Vaccines (ACTIV) public-private partnership platform. Its main goals were *"to establish a collaborative framework for prioritizing vaccine and therapeutic candidates, to streamline clinical trials and tap into existing clinical trial networks"* [36].

In one arm of the international project in which the NIH-funded ACTIV-4a combined with two other platform trials (the Canadian-funded ATTACC, and the widely-funded REMAP-CAP) the highest number of patients were enrolled in the US. The trials worked together to show that in noncritically ill patients with COVID-19, an initial strategy of therapeutic-dose anticoagulation with heparin increased the probability of survival to hospital discharge [37]. In the three platforms' parallel trial of the treatment with critically ill patients, REMAP-CAP took the lead and most patients were enrolled in the UK, but the treatment was not successful [38].

Slaoui and colleagues later described how OWS's role in accelerating therapeutics and diagnostics included working selectively with ACTIV in a series of areas [39]. To assess treatments such as mAbs two master protocols were established - ACTIV-2 for out-patient trials and ACTIV-3 for in-patient trials [39] (see below).

Increasingly the results were announced of trials from the private sector, sometimes with NIH/OWS support. Three companies successfully conducted trials identifying the benefits of early mAb treatments. As noted the Canadian company based in Vancouver, AbCellera, had developed an advanced technology. As a result of that, since 2018 it had been funded by the Pandemic Prevention Platform of the US's DARPA *"to establish a robust technology platform for pandemic response capable of developing field-ready medical countermeasures within 60 days of isolation of an unknown viral pathogen"* [40]. This meant it was ready to go in January 2020. The US company Eli Lilly took on development of bamlanivimab after its rapid discovery by AbCellara who worked in conjunction with NIAID's Vaccine Research Centre [41,42]. Ely Lilly conducted their successful trial of bamlanivimab plus etesevimab for nonhospitalised patients entirely in the US. It was published in the *NEJM* [42]. There were also trials of bamlanivimab in ACTIV-2 and ACTIV-3 [39]. The *NEJM* published a report of an OWS/NIH-funded ACTIV-3/TICO (Therapeutics for Inpatients with COVID-19) platform trial of the mAb in hospitalised patients that was halted when futility analysis showed lack of benefit [43].

A further study published in *JAMA* in June 2021 showed that bamlanivimab as a monotherapy reduced the incidence of infection among residents and staff of skilled nursing facilities. It was sponsored by Eli Lilly and conducted in partnership with the NIAID and the COVID-19 Prevention Network [44]. An editorial on the paper highlighted both the success of the trial in mobilising a federally funded clinical trials network to support the company trial, but also the challenges around using bamlanivimab as a monotherapy after the FDA had withdrawn its original Emergency Use Authorisation for its use as a monotherapy because the mAb was thought to be less effective against some new variants [45].

In July 2020 OWS had announced REGN-COV2 (later REGEN-COV), a neutralizing antibody cocktail of casirivimab plus imdevimab developed by Regeneron, was intended to be the first candidate therapeutic it would take through to commercial manufacturing with a USD450m investment [39]. In December 2020 a report of the earlier phase of the trial supported by BARDA showed success in reducing viral load in outpatients [46]. Its effectiveness in reducing the risk of COVID-19 related hospitalisation, or death from any cause, was reported online in the *NEJM* in September 2021 [47], and in August it had been shown to reduce the risk of COVID-19 in contacts of infected persons [48]. The successful trial of sotrovimab, the third mAb, developed by a biotech company and the US arm

of GKS, had a first author from a Canadian hospital, other trial sites in Brazil and Spain, but most sites in the US [49].

The FDA permitted use of all three mAbs (in the case of bamlanivimab, only in combination with etesevimab) as COVID-19 treatments for at risk nonhospitalised patients and they were included in the relevant NIH guideline [50]. (See next section for details and updates.)

Following further complicated developments described below and in the next section, AbCellera discovered a new mAb, bebtelovimab, that was effective against Omicron and that again Eli Lilly developed [51].

In 2021, two US companies first, Merck Sharp and Dohme, second Pfizer, announced they had successfully developed and trialled COVID-19 specific antiviral pills for non-hospitalised adults within five days of the onset of symptoms [52-54]. Through early stage research funded prior to the pandemic by the NIAID and others, a team at Emory University, Georgia, US, had invented the antiviral molnupiravir that was then developed and tested by Ridgeback Biotherapeutics and Merck Sharp and Dohme [55,56]. The Merck trial of molnupiravir was conducted in many countries, but the lead author was from a Columbian institution, the second from Brazil, and the corresponding author from the company's US base [52]. The Pfizer trial was mostly in the US, but also included sites in various countries including almost 10% in Brazil [53,54].

Section 2: *Examples of achievements in promoting evidence use in products, policymaking and practice; examples (and lessons) about links to existing strengths*

A series of vaccines starting with Pfizer/ BioNTech and Moderna, and then Johnson and Johnson, were successfully brought through to regulatory approval and application even more rapidly than research leaders had predicted in Spring 2020 [36]. As noted, the OWS funding for advanced purchases enabled the early manufacturing to run in parallel with late stage development. In the case of the relatively new biotech company Moderna, for example, it helped build up manufacturing capacity [6,15,57]. The vaccine rollout rapidly expanded in 2021 [58] and built on the development success so that it was estimated the vaccines had saved 140,000 lives in the US in the first four months [59] and over one million by December 2021 [60].

By directly conducting important studies drawing on their own databases some large healthcare organisations such as Kaiser Permanante Southern California could inform their policy, e.g., on the nature of the waning effectiveness of COVID-19 vaccines and the need for booster doses [20].

Many other studies provided valuable findings that informed policy and practice. The RADx initiative attempted to address the initial problems related to testing, including a lack of coordination. The initiative's various successes were reported to include helping to contain outbreaks in schools by providing test kits across the country [61].

The NIH introduced COVID-19 treatment guidelines, updated frequently as published data and other authoritative information became available. One for Anti-SARS-CoV-2 Antibody Products had a specific guideline for mAbs that focused on the three mAbs for nonhospitalised patients described in section 1, but again including bamlanivimab plus etesevimab, rather than bamlanivimab as a monotherapy [50]. In line with the various complexities listed in section 1, the guideline was

updated on several occasions. This included on 16 December 2021 following an FDA expansion of Emergency Use Authorisation which had originally been given to all three *"for the treatment of mild to moderate COVID-19 in nonhospitalised patients with laboratory confirmed SARS-CoV-2 infection who are at high risk for progressing to severe disease and/or hospitalization"* [50]. The categories of patients to whom bamlanivimab plus etesevimab could be given were expanded to include children.

The guideline also referred to the ACTIV-3 study in hospitalised patients [43] as justification for not extending the recommendation for use to such patients. This was despite acknowledging that a study by RECOVERY showed there were benefits from REGEN-COV for hospitalised patients who were seronegative [50]. One major factor in not including an extension to such patients was that rapid serology testing that could identify seronegative individuals in real time was not widely available in the US, unlike the UK. The guideline recognised that the FDA had expanded its authorisation to cover the use of bamlanivimab plus etesevimab, and casirivimab plus imdevimab as post-exposure prophylaxis for individuals at high risk of both acquiring the infection, and then progressing to serious illness. The guideline did not itself include a recommendation for such use, but gave a link to a separate NIH guideline covering prevention of SARS-CoV-2 infection [50].

In December 2021 the FDA permitted use of both antivirals described above [56,62]. However, the approval for Merck's molnupiravir was rather limited in just being for patients with mild-to-moderate COVID-19 at high risk of progression to severe COVID-19, including hospitalisation or death, for whom alternative COVID-19 treatment options were not clinically appropriate, or accessible [56].

(While strictly outside our period, in January 2022 the FDA removed its authorisation for the mAbs apart from sotrovimab because they were found to be not effective against Omicron [63], but then in February 2022 gave limited authorisation to AbCellera's new mAb, bebtelovimab, that was effective against Omicron and again had been developed by Eli Lilly [51]. In April 2022 the FDA removed its authorisation for sotrovimab because of the increase in the proportion of COVID-19 cases caused by the Omicron BA.2 sub-variant against which it was unlikely to be effective [64]).

Apart from within bodies such as the NIH, how far the science community has been successfully able to inform policy varied greatly between (and even within) different US states (see also next section), but in some states policymakers have been much more willing than others to listen to the scientific evidence [65].

At the national level, four public health experts were asked towards the end of 2021 how far they thought President Biden had been able to keep to his promises such as listening to the science [58]. As noted above, they said *"the president's vaccine rollout, overall, was excellent"* [58]. They felt the messaging had been off at times, and some setbacks *"both within and beyond his control"* had limited the progress. But overall *"Biden followed the science, said the experts. Plus, implementing regular covid media briefings with scientist and public health leaders, and allowing Dr. Anthony Fauci, his chief medical adviser, to be front and center and not contradicting his advice, represented a meaningful change from the previous administration"* [58].

Section 3: *Examples of challenges in: 1. production of evidence; 2. use of evidence; and 3. sustaining the research system; examples of lessons to address challenges*

Challenges in production of evidence

Publication of the RECOVERY trial in the UK led commentators to analyse why the US was not making the same rapid progress in identifying re-purposed drugs that were, or were not, effective against COVID-19. (See UK section.) Factors noted included that the trial was being conducted across UK hospitals - *"incorporated as part of everyday clinical care"* [66] - keeping things simple, and preventing duplication of many small studies [67].

In a *NEJM* editorial on 17 July 2020 accompanying its publication of the RECOVERY trial's dexamethasone findings [68], Lane and Fauci contrasted the US's many small, non-conclusive therapy trials with RECOVERY's rapid, but conclusive, findings [69]. The way forward was with approaches such as the ACTIV programme [69]. Other new platform trials also attempted to address the challenge of accelerating /coordinating the research of US teams [70].

In March 2021 Angus, together with Anthony Gordon, principal investigator of the UK arm of the REMAP-CAP, and Howard Bauchner, Editor in Chief of *JAMA*, noted that only a few thousand out of the 30 million US citizens who had developed COVID-19 had been entered into RCTs [71]. They acknowledged some progress, but said: *"The problem is not lack of intent, effort or resources...The main challenges appear to be (1) the structure of the clinical research enterprise and (2) the interface between clinical research and clinical care"* [71]. The clinical research enterprise in the US was set up like a marketplace where *"There is no oversight to ensure research questions are prioritized",* and while individual funders may have priorities they were largely autonomous [71].

The problems were apparent despite the good work to create the ACTIV program: *"The problems of poor coordination, limited incentives for collaboration and lack of priorization...were immediately evident....However, the structure of the US clinical research enterprise is vast and has never functioned as a single national coordinated system"* [71]. Having described the second challenge as the lack of any agency that *"oversees ensuring integrated coordination between research and care"* they joined others who looked to learn lessons from the UK's response and described how the CRN had been build up over many years [71].

Lessons proposed for clinical research included more effective planning, payment of clinicians through an independent national system for participating in trials on national priorities, simplifying trial protocols, and embracing data flows across clinical care and research [71]. The first priority area for them related to implementing *"A US version of the NIHR CRN hospital payment system for trial participation"* [71]. Under this heading they stated: *"It is crucial that the health care delivery system (and clinical care) adopts the contribution to research as a core value and is rewarded via a mechanism that is independent of any research project or investigator"* [71].

There was considerable racial disproportionality in COVID-19 clinical trials in the US with underrepresentation of minority groups, despite the existence of many strategies to increase enrolment of diverse population [72,73]. In October 2021, Janet Woodcock, FDA acting Commissioner, and colleagues said problems facing US trials included insufficient diversity and slow enrolment. Both could be addressed by integrating research into community practice, the source of healthcare for many Black and other Americans underrepresented in trials. Increasing the pool of clinical researchers would also *"make it easier to translate from research results to clinical care"* [72].

Various complex Intellectual Property issues and disputes were highlighted during the pandemic, especially around mRNA vaccines [3-5].

<u>Challenges in use of evidence</u>

Despite government support and promotion of research through the OWS, evidence use was highly variable - the lack of coordination and multiple policymakers at many levels, "*combined with the intense polarization of US politics and confusing messages from President Trump, produced wide variation in state-level policies"* [65].

The cross-country analysis published in September 2020 by Tworek et al., of the communications used by authorities in nine countries with at least a reasonably effective response to COVID-19, contrasted them *"with democracies that struggled to communicate around Covid-19, particularly the United Kingdom and the United States"* [74]. Journalists commenting on this noted that whereas some governments have been praised for the science-driven way that they have communicated about the pandemic, others *"most notably the U.S. and the U.K., have been hit with criticism for public health messages that are confusing or not based in science"* [75].

In late 2020 a *NEJM* editorial criticised how experts were ignored or denigrated: *"Our current leaders have undercut trust in science and in government, causing damage that will certainly outlast them. Instead of relying on expertise, the administration has turned to uninformed "opinion leaders" and charlatans who obscure the truth and facilitate the promulgation of outright lies"* [76].

As noted above in the Brazilian section, comparisons were made by various authors between Presidents Trump and Bolsonaro, and another analysis suggested *"Both leaders repeatedly resisted recommendations made by scientific experts"* [77]. That created intense problems despite the federal nature of both countries because while in both countries individual states developed many of their own pandemic policies, *"the approaches of both nation's leaders have put their large countries at risk and have made it more difficult for local leaders to develop effective policy"* [77].

Conversely, in 2021, some of the states opposed certain measures of the Biden Administration, such as mask mandates [58], which the evidence described in the Germany section had shown to be effective [78]. This is consistent with the *NEJM* editorial about the damage done by the Trump administration in undercutting trust in science and in government.

Table 1 in our main paper showed the excess death figures were very high in the US, and an analysis of data from March to July 2020 showed them to be much worse than in Europe [79]. An analysis of excess years of life lost in 2020 across 37 countries, found comparatively the US did even worse than it did on the measure of deaths because of the younger average age of US COVID-19 victims [80].

Reflecting on the US's top score on the 2019 GHS Index, it was noted in April 2020 the Index *"did not anticipate the poor response to the pandemic by high-scoring countries such as the US where major gaps in federal leadership resulted in a failure to mobilize the country's substantial capacity"* [81]. However, it is a composite index with many elements. For example, the US, despite being ranked highest overall, was 175th out of 195 countries on the item for healthcare access, and had the lowest possible score of zero – it failed to reach the 25% threshold - for public confidence in the government, which is very serious because lack of public trust is likely to undermine disease-control

and public health messages [81]. It is likely, as noted by the *NEMJ* above, that the approach of President Trump further undermined trust during the pandemic.

According to one researcher looking at the high scores but poor performance by the UK and US: *"we had everything – except leadership"* [82]. One of the GHS Index team was quoted as saying: *"Even though the US and UK had the best environments in terms of plans in place and thinking about what they would need in terms of capacity...when it came to the moment that everyone had been preparing for, the decision-making really hampered the actual ability of the country to respond"* [82].

Challenges in sustaining the research system

Fifty-five percent of NIH researchers responding to its survey in 2020 said the pandemic would have a negative impact on their career trajectory; 78% reported lower productivity levels [83].

Summary

The US went into the pandemic with the world's most extensive health research capacity and first place in the 2019 GHS Index. Biomedical scientists across the US had worked for years exploring the possibility of using mRNA, but a key role was played by Katalin Karikó and Drew Weissman at the University of Pennsylvania who showed the importance of modifying mRNA. This encouraged the creation of the biotech company Moderna, which immediately started developing their COVID-19 vaccine once the sequence of the genome was released. They intensified their work with Anthony Fauci's NIAID on a series of animal and human trials reported in the *NEJM*. More generally, however, there was a lack of coordination in the clinical research response, and considerable waste reported. In April 2020, the NIH created the ACTIV public/private partnership to coordinate and accelerate trials. In May 2020, the Government took this further with the creation of OWS which aimed to facilitate, at an unprecedented pace, the development, manufacturing, and distribution of COVID-19 countermeasures, especially vaccines and therapies. It prioritised its support but contributed to the development and early manufacture of Moderna's vaccine, and, with advanced purchase agreements, the early manufacture of Pfizer's vaccine. Various public/private collaborations also helped develop new therapies such as mAbs, and also diagnostics. However, the US had a very high death rate. This was widely blamed on President Trump because of his frequently dismissive attitude towards the scientific evidence in both his policymaking and his communications, and was exacerbated by the inequalities which resulted in particularly high levels of cases and deaths among minority communities.

References

1. Norton A, Bucher A, Antonio E, Advani N, Grund H, Mburu S, et al. A living mapping review for COVID-19 funded research projects: nine-month update [version 4; peer review: 2 approved]. Wellcome Open Research 2021; doi: https://doi.org/10.12688/wellcomeopenres.16259.4.
2. Kim K, Cho KT. A review of global collaboration on COVID-19 research during the pandemic in 2020. Sustainability, 2021; doi: https://doi.org/10.3390/su13147618.
3. Dolgin E. The tangled history of mRNA vaccines. Nature. 2021; doi: https://doi.org/10.1038/d41586-021-02483-w.
4. Allen A. For billion-dollar COVID vaccines, basic government-funded science laid the groundwork. Kaiser Health Newsletter, 18 Nov 2020. For Billion-Dollar COVID Vaccines, Basic Government-Funded Science Laid the Groundwork - Scientific American. Accessed 8 Jan 2022.

5. Gaviria M, Kilic B. A network analysis of COVID-19 mRNA vaccine patents. Nature Biotechnology. 2021; doi: https://doi.org/10.1038/s41587-021-00912-9.
6. Hanney SR, Wooding S, Sussex J, Grant J. From COVID-19 research to vaccine application: why might it take 17 months not 17 years and what are the wider lessons? Health Res Policy Sys. 2020; doi: https://doi.org/10.1186/s12961-020-00571-3.
7. Baden LR, El Sahly HM, Essink B, Kotloff K, Frey S, Novak R, et al. Efficacy and safety of the mRNA-1273 SARS-CoV-2 vaccine. NEJM. 2021; doi: 10.1056/NEJMoa2035389.
8. Jackson LA, Anderson EJ, Rouphael NG, Roberts PC, Makhene M, Coler RN, et al. An mRNA vaccine against SARS-CoV-2 — preliminary report. NEJM. 2020;383:1920-31.
9. Corbett KS, Flynn B, Foulds KE, Boyoglu-Barnum S, Werner AP, et al. Evaluation of the mRNA-1273 vaccine against SARS-CoV-2 in nonhuman primates. NEJM. 2020; 383:1544-55.
10. Anderson EJ, Rouphael NG, Widge AT, Jackson LA, Roberts PC, Makhene M, et al. Safety and immunogenicity of SARS-CoV-2 mRNA-1273 vaccine in older adults. NEJM 2020; 383:2427-38.
11. Gilbert S, Green C. Vaxxers. The inside story of the Oxford AstraZeneca vaccine and the race against the virus. London: Hodder and Stoughton; 2021.
12. van Doremalen N, Lambe T, Spencer A, Belij-Rammerstorfer S, Purushotham JN, Port, JR, et al. ChAdOx1 nCoV-19 vaccine prevents SARS-CoV-2 pneumonia in rhesus macaques. Nature. 2020; doi: https://doi.org/10.1038/s41586-020-2608-y.
13. US Department of Health and Human Services. Trump administration announces framework and leadership for 'Operation Warp Speed'. HHS press office, 15 May 2020. 2021-01-20 12:29 | Archive of HHS – About News (pagefreezer.com). Accessed 10 Jan 2022.
14. Winch GM, Cao D, Maytorena-Sanchez E, Pinto J, Sergeeva N, Zhang, S. Operation Warp Speed: Projects responding to the COVID-19 pandemic. Project Leadership and Society. 2021; doi: https://doi.org/10.1016/j.plas.2021.100019.
15. Lancet Commission on COVID-19 Vaccines and Therapeutics Task Force Members. Operation Warp Speed: implications for global vaccine security. Lancet Glob Health. 2021; doi: https://doi.org/10.1016/S2214-109X(21)00140-6
16. Polark FP, Thomas SJ, Kitchin N, Absalon J, Gurtman A, Lockhart S, et al. Safety and efficacy of the BNT162b2 mRNA Covid-19 vaccine. NEJM. 2020; doi: 10.1056/NEJMoa2034577 https://www.nejm.org/doi/full/10.1056/NEJMoa203457.
17. Lurie N, Saville M, Hatchett R, Halton J. Developing covid-19 vaccines at pandemic speed. NEJM. 2020; doi: https://doi.org/10.1056/NEJMp2005630.
18. Hackethal V. How we got a COVID vaccine so quickly. Sharecare, 19/1/21 (updated). How We Got a COVID Vaccine So Quickly - Sharecare. Accessed 19 Jan 2022.
19. Wentzell E, Racila A-M. The social experience of participation in a COVID-19 vaccine trial: subjects' motivations, others' concerns, and insights for vaccine promotion. Vaccine. 2021; doi: 10.1016/j.vaccine.2021.03.036.
20. Tartof SY, Slezak JM, Fischer H, Hong V, Ackerson BK, Ranasinghe ON, et al. Effectiveness of mRNA BNT162b2 COVID-19 vaccine up to 6 months in a large integrated health system in the US: a retrospective cohort study. Lancet. 2021; doi:10.1016/S0140-6736(21)02183-8.
21. Dickerman BA, Gerlovin H, Madenci AL, Kurgansky KE, Ferolito BR, Figeuroa Muniz MJ, et al. Comparative effectiveness of BNT162b2 and mRNA-1273 vaccines in U.S. veterans. NEJM. 2022: doi: 10.1056/NEJMoa2115463.
22. NIH. Rapid Acceleration of Diagnostics (RADx®): Overview. NIH. RADx | National Institutes of Health (NIH). Accessed 19 Jan 2022. NIH.
23. Kelly M, Cahlan S, Samuels E. What went wrong with coronavirus testing in the U.S. The Washington Post. 30 March 2020. What went wrong with coronavirus testing in the U.S. - The Washington Post Accessed 4 May 2022.
24. Tromberg BJ, Schwetz TA, Pérez-Stable EJ, Hodes RJ, Woychik RP, Bright RA et al. Rapid scaling up of COVID-19 diagnostic testing in the United-States: the NIH's RADx Initiative. NEJM. 2020;383:1071–7. DOI: 10.1056/NEJMsr2022263(link is external).

25. NIH. RADx Programs. NIH. Updated 16 November 2021. RADx Programs | National Institutes of Health (NIH) Accessed 13 May 2022.
26. NIH. NIH to assess and expand COVID-19 testing for underserved communities [RADx-UP]. NIH. 30 September 2020. NIH to assess and expand COVID-19 testing for underserved communities | National Institutes of Health (NIH) Accessed 12 May 2022.
27. NIH. Final report confirms remdesivir benefits for COVID-19. NIH Research matters, 20 Oct 2020. Final report confirms remdesivir benefits for COVID-19 | National Institutes of Health (NIH) Accessed 4 Jan 2022.
28. Beigel JH, Tomashek KM, Dodd LE, Mehta, AK, Zingman BS, Kalil AC, et al. Remdesivir for the treatment of Covid-19 — final report. NEJM. 2020; doi: 10.1056/NEJMoa2007764.
29. Kalil AC, Patterson TF, Mehta AK, Tomashek KM, Wolfe CR, Ghazaryan V, et al. Baricitinib plus remdesivir for hospitalized adults with Covid-19. NEJM. 2021; doi: 10.1056/NEJMoa2031994.
30. WHO Solidarity Trial Consortium. Repurposed antiviral drugs for COVID-19; interim WHO Solidarity trial results. Preprint. 2020; doi: https://doi.org/10.1101/2020.10.15.20209817.
31. WHO Solidarity Trial Consortium. Repurposed antiviral drugs for Covid-19 — interim WHO Solidarity trial results. NEJM. 2021; doi: https://doi.org/10.1056/NEJMoa2023184.
32. Rochwerg B, Agoritsas T, Lamontagne F, Yee-Sin L, Macdonald H, Agarwal A, et al. A living WHO guideline on drugs for covid-19. BMJ.2020; doi: http://dx.doi.org/10.1136/bmj.m3379.
33. REMAP-CAP. REMAP-CAP: A randomised, embedded, multi-factorial, adaptive platform trial for community-acquired pneumonia. REMAP-CAP Trial (remapcap.org). Accessed 7 Jan 2022.
34. The Writing Committee for the REMAP-CAP Investigators. Effect of hydrocortisone on mortality and organ support in patients with severe COVID-19: The REMAP-CAP COVID-19 corticosteroid domain randomized clinical trial. JAMA. 2020; doi:10.1001/jama.2020.17022.
35. NIHR. Case study: REMAP-CAP tests treatments for COVID-19. NIHR, Original version: 18 Sept 2020, updated regularly. https://www.nihr.ac.uk/case-studies/remap-cap-tests-treatments-for-covid-19/25044. Accessed 17 Jan 2022.
36. Collins FS, Stoffels P. Accelerating COVID-19 therapeutic interventions and vaccines (ACTIV): an unprecedented partnership for unprecedented times. JAMA. 2020; doi:10.1001/jama.2020.8920.
37. The ATTACC, ACTIV-4a, and REMAP-CAP Investigators. Therapeutic anticoagulation with heparin in noncritically ill patients with Covid-19. NEJM. 2021; doi: 10.1056/NEJMoa2105911.
38. The REMAP-CAP, ACTIV-4a, and ATTACC Investigators. Therapeutic anticoagulation with Heparin in critically ill patients with Covid-19. NEJM. 2021; doi: 10.1056/NEJMoa2103417.
39. Slaoui M, Greene SE, Woodcock J. Bridging the gap at warp speed — delivering options for preventing and treating Covid-19. NEJM. 2020; doi: 10.1056/NEJMp2028535.
40. AbCellera. AbCellera is mobilizing a response to the novel coronavirus outbreak as part of its DARPA P3 program. AbCellera press release, 28 Jan 2020. AbCellera is mobilizing a response to the novel coronavirus outbreak as part of its DARPA P3 program. Accessed 16 Jan 2022.
41. AbCellera. Emergency use authorization of AbCellera-ciscovered Bamlanivimab administered with Etesevimab expanded by the FDA to include post-exposure prophylaxis for the prevention of COVID-19. AbCellera press release, 16 Sept 2021. Emergency Use Authorization of AbCellera-Discovered Bamlanivimab Administered with Etesevimab Expanded by the FDA to Include Post-Exposure Prophylaxis for the Prevention of COVID-19. Accessed 16 Jan 2022.
42. Dougan M, Nirula A, Azizad M, Mocherla B, Gottleib RL, Chen P, et al. Bamlanivimab plus etesevimab in mild or moderate Covid-19. NEJM 2021; doi: 10.1056/NEJMoa2102685.
43. ACTIV-3/TICO LY-CoV555 Study Group. A neutralizing monoclonal antibody for hospitalized patients with COVID-19. NEJM. 2021; doi: 10.1056/NEJMoa2033130.
44. Cohen MS, Nirula A, Mulligan MJ, Novak RM, Marovich M, Yen C, et al. Effect of Bamlanivimab vs placebo on incidence of COVID-19 among residents and staff of skilled nursing and assisted living facilities: a randomized clinical trial. JAMA. 2021; doi: 10.1001/jama.2021.8828.
45. Kuritzkes DR. Bamlanivimab for prevention of COVID-19. JAMA. 2021; doi:10.1001/jama.2021.7515.

46. Weinreich DB, Sivapalasingam S, Norton T, Ali S, Gao H, Bhore R, et al. REGN-COV2, a neutralising antibody cocktail, in outpatients with Covid-19. NEJM 2021; doi: 10.1056/NEJMoa2035002.
47. Weinreich DB, Sivapalasingam S, Norton T, Ali S, Gao H, Bhore R, et al. REGEN-COV antibody combination and outcomes in oupatients with Covid-19. NEJM. 2021; doi: 10.1056/NEJMoa2108163.
48. O'Brien MP, Forleo-Neto E, Musser BJ, Isa F, Chan K-C, Sarkar N, et al. Subcutaneous REGEN-COV antibody combination to prevent COVID-19. NEJM. 2021;385(13):1184-95.
49. Gupta A, Gonzalez-Rojas Y, Juarez E, Casal, MC, Moya, J, Falci, DR, et al. Early treatment for Covid-19 with SARS-CoV-2 neutralizing antibody sotrovimab. NEJM 2021; doi: 10.1056/NEJMoa2107934.
50. NIH. Anti-SARS-CoV-2 Monoclonal Antibodies | COVID-19 Treatment Guidelines. NIH, 16 Dec 2021. Anti-SARS-CoV-2 Monoclonal Antibodies | COVID-19 Treatment Guidelines (nih.gov). Accessed 20 Jan 2022.
51. AbCellera. AbCellera-Discovered Antibody, Bebtelovimab, Receives U.S. FDA Emergency Use Authorization for the Treatment of Mild-to-Moderate COVID-19. AbCellera. 11 Feb 2022. AbCellera-Discovered Antibody, Bebtelovimab, Receives U.S. FDA Emergency Use Authorization for the Treatment of Mild-to-Moderate COVID-19 Accessed 13 Feb 2022.
52. Jayk Bernal A, Gomes da Silva MM, Musungaie DB, Kovalchuk E, Gonzalez A, Delos Reyes D, et al. Molnupiravir for oral treatment of Covid-19 in nonhospitalized patients. NEJM. 2021; doi: 10.1056/NEJMoa2116044.
53. Pfizer. Pfizer announces additional phase 2/3 study results confirming robust efficacy of novel COVID-19 oral antiviral treatment candidate in reducing risk of hospitalization or death. Pfizer, 14 Dec 2021. Pfizer Announces Additional Phase 2/3 Study Results Confirming Robust Efficacy of Novel COVID-19 Oral Antiviral Treatment Candidate in Reducing Risk of Hospitalization or Death | Pfizer. Accessed 24 Dec 2021.
54. NIH/US National Library of Medicine/ClinicalTrials.gov. Evaluation of protease inhibition for COVID-19 in standard-risk patients (EPIC-SR). Identifier: NCT05011513. Evaluation of Protease Inhibition for COVID-19 in Standard-Risk Patients (EPIC-SR). - Full Text View - ClinicalTrials.gov. Accessed 24 Dec 2021.
55. Painter GR, Natchus MG, Cohen O, Holman W, Painter WP. Developing a direct acting, orally available antiviral agent in a pandemic: the evolution of molnupiravir as a potential treatment for COVID-19. Curr Opin Virol. 2021 Oct;50:17-22. doi: 10.1016/j.coviro.2021.06.003
56. Emory University. Antiviral drug discovered at Emory receives authorization from FDA. Emory News Centre, 23 Dec 2021. Antiviral drug discovered at Emory receives authorization from FDA | Emory University | Atlanta GA. Accessed 7 Jan 2022.
57. Moderna. Moderna and Lonza announce worldwide strategic collaboration to manufacture moderna's vaccine (mRNA-1273) against novel coronavirus. 2020. https://investors.modernatx.com/news-releases/news-release-details/moderna-and-lonza-announce-worldwide-strategic-collaboration. Accessed 20 Jan 2022.
58. Knight V. A look at what happened in 2021 and whether Biden's efforts have made a difference. Kaiser Health News, 20 Dec 2021. The Vaccine Rollout Was a Success. But Events Within and Beyond Biden's Control Stymied Progress. | Kaiser Health News (khn.org). Accessed 3 Jan 2022.
59. Gupta S, Cantor J, Simon KI, Bento AI, Wing C, Whaley CM. Vaccinations against COVID-19 may have averted up to 140,000 deaths in the United States. Health Aff (Millwood). 2021; doi: 10.1377/hlthaff.2021.00619.
60. Schneider EC, Shah A, Sah P, Moghadas SM, Vilches T, Galvani A. The U.S. COVID-19 vaccination program at one year: how many deaths and hospitalizations were averted? Commonwealth Fund, Dec. 2021. https://doi.org/10.26099/3542-5n54. Accessed 3 Jan 2022.
61. Oren E. Medical technologies have been central to US pandemic response – but social behaviors matter just as much. The Conversation. 22 Dec 2021. Medical technologies have been

central to US pandemic response – but social behaviors matter just as much (theconversation.com). Accessed 11Aug 2022.
62. US Food and Drug Administration. Coronavirus (COVID-19) Update: FDA authorizes first oral antiviral for treatment of COVID-19. FDA News Release, 22 Dec 2021. Coronavirus (COVID-19) Update: FDA Authorizes First Oral Antiviral for Treatment of COVID-19 | FDA. Accessed 7 Jan 2021.
63. FDA. FDA Revokes EUAs for Two Monoclonal Antibodies Shown to be Ineffective Against Omicron.FDA.26 Jan 2022. FDA Revokes EUAs for Two Monoclonal Antibodies Shown to be Ineffective Against Omicron | 2022-01-25 | FDAnews Accessed 31 Jan 2022.
64. FDA. FDA updates Sotrovimab emergency use authorization. FDA. 5 April 2022. FDA updates Sotrovimab emergency use authorization | FDA Accessed 13 May 2022.
65. Hurlbutt JB, Hilgartner S, Jasanoff S, Ozgode O, Rayzberg M. United States. In: Jasanoff S, Hilgartner SJ, Hurlbut JB, Ozgode O, Rayzberg M. Comparative Covid response: crisis, knowledge, politics. Interim report. 12 Jan 2021. https://compcore.cornell.edu/wp-content/uploads/2021/03/Comparative-Covid-Response_Crisis-Knowledge-Politics_Interim-Report.pdf Accessed 12 Jan 2022.
66. Emanuel EJ, Zhang C, Diana A. Where Is America's groundbreaking Covid-19 research? The U.S. could learn a lot from Britain. New York Times, 1 Sept, 2020. https://www.nytimes.com/2020/09/01/opinion/coronavirus-clinical-research.html?smid=em-share. Accessed 7 Sept 2020.
67. Couzin-Frankel J. 'We've got to be able to move more quickly.' The pandemic reality of COVID-19 clinical trials. Science. June 16, 2020. https://www.sciencemag.org/news/2020/06/we-ve-got-be-able-move-more-quickly-pandemic-reality-covid-19-clinical-trials. Accessed 10 Jan 2022.
68. RECOVERY Collaborative Group. Dexamethasone in hospitalized patients with Covid-19. NEJM. 2021; doi: 10.1056/NEJMoa2021436.
69. Lane HC, Fauci AS. Research in the context of a pandemic. NEJM. 2020; doi: 10.1056/NEJMe2024638. Lane HC, Fauci AS. Research in the context of a pandemic. NEJM. 2020; doi: 10.1056/NEJMe2024638.
70. Hedlin H, Garcia A, Weng Y, He Z, Sundaram V, Bunning B, et al. Clinical trials in a COVID-19 pandemic: shared infrastructure for continuous learning in a rapidly changing landscape. Clinical Trials. 2021; doi: 10.1177/1740774520988298.
71. Angus DC, Gordon AC, Bauchner H. Emerging lessons from COVID-19 for the US clinical research enterprise. JAMA. 2021; doi:10.1001/jama.2021.3284.
72. Woodcock J, Araojo R, Thompson T, Puckrein GA. Integrating research into community practice — toward increased diversity in clinical trials. NEJM. 2020; doi: 10.1056/NEJMp2107331.
73. Chastain DB, Osae SP, Henao-Martínez AF, Franco-Paredes C, Chastain JS, Young HN. Racial disproportionality in Covid clinical trials. NEJM 2020;383(9):e59-e59. DOI: 10.1056/NEJMp2021971
74. Tworek H, Beacock I, Ojo E. Democratic Health Communications during Covid-19: A RAPID Response. Vancouver: UBC Centre for the Study of Democratic Institutions, September 2020. Democratic-Health-Communication-during-Covid_FINAL.pdf (ubc.ca) Accessed 4 May 2022.
75. Robbins R, Garde D, Feuerstein A. An expert's take on what the U.S., U.K did wrong in Covid-19 communications — and what others did right. STAT. 18 Sept 2020. What the U.S. did wrong on Covid-19 — and what others did right (statnews.com) Accessed 4 May 2020.
76. New England Journal of Medicine, (editorial). Dying in a leadership vacuum. NEJM. 2020; doi 10.1056/NEJMe2029812.
77. Riley C, Xie B, Khurshid A. Challenges encountered in comparing international policy responses to COVID-19 and their effects. Health Res Policy Sys. 2021; doi: https://doi.org/10.1186/s12961-021-00783-1
78. Mitze T, Kosfeld R, Rode J, Wälde K. Face masks considerably reduce COVID-19 cases in Germany. Proc. Natl Acad. Sci. 2020; doi: https://doi.org/10.1073/pnas.2015954117.
79. Aron J, Muellbauer J. The US excess mortality rate from COVID-19 is substantially worse than Europe's | VOX, CEPR Policy Portal (voxeu.org); 29 Sept 2020. Accessed 20 Jan 2022.

80. Islam N, Jdanov DA, Shkolnikov VM, Khunti K, Kawachi I, White M, et al. Effects of covid-19 pandemic on life expectancy and premature mortality in 2020: time series analysis in 37 countries. BMJ. 2021; doi: https://doi.org/10.1136/bmj-2021-066768.
81. GHS Index. The U.S. and COVID-19: Leading the World by GHS Index Score, not by Response. GHS Index. The U.S. and COVID-19: Leading the World by GHS Index Score, not by Response - GHS Index. Accessed 5 Feb 2022.
82. York C. The UK And US Were Ranked Top For Pandemic Preparedness. What Went Wrong? HuffPost UK. 27 Jan 2021 (updated). The UK And US Were Ranked Top For Pandemic Preparedness. What Went Wrong? | HuffPost UK News (huffingtonpost.co.uk) Accessed 4 May 2022.
83. NIH Grants and Funding. The Impact of COVID-19 on the Research Community | grants.nih.gov NIH. Accessed 6 Sept 2021.

Abbreviations

AAMRI: Association of Australian Medical Research Institutes; ACTIV: Accelerating COVID-19 Therapeutic Interventions and Vaccines; APPRISE: Australian Partnership for Preparedness Research on Infectious Disease Emergencies; ASCOT: AustralaSian COVID-19 Trial; ATTACC: Antithrombotic Therapy to Ameliorate Complications of COVID-19; AWMF: Association of the Scientific Medical Societies in Germany; BARDA: Biomedical Advanced Research and Development Authority (US); BC: British Columbia; BMBF: Federal Ministry of Education and Research (Germany): CEOsys: The COVID-19 Evidence Ecosystem (Germany); CIHR: Canadian Institutes of Health Research; CIU: Critical Intelligence Unit (NSW, Australia); COG-UK: COVID-19 Genomics UK Consortium; CRN: Clinical Research Network (England); DARPA: Defense Advanced Research Projects Agency (US); EU: European Union; FDA: Food and Drug Administration (US); Fiocruz: Oswaldo Cruz Foundation (Brazil); GHS: Global Health Security; HCQ: hydroxychloroquine; HRC: Health Research Council (New Zealand); HRS: health research system; JAMA: Journal of the American Medical Association; lmic: low- and middle-income country; mAb: monoclonal antibody; MHRA: Medicines and Healthcare products Regulatory Agency UK); MRC: Medical Research Council (UK); mRNA: messenger ribonucleic acid; NHMRC: National Health and Medical Research Council (Australia); NHS: National Health Service (UK); NIAID: National Institute of Allergy and Infectious Diseases (US); NEJM: the New England Journal of Medicine; NIH: National Institutes of Health (US); NIHR: National Institute for Health Research (England); NPI: non-pharmaceutical interventions; NSW: New South Wales; OWS: Operation Warp Speed (US); PAHO: Pan-American Health Organization; PCR polymerase chain reaction; PM: Prime Minister; PPI: patient and public involvement; RADx: Rapid Acceleration of Diagnostics (US); RADx-UP: Rapid Acceleration of Diagnostics-Underserved Populations (US); RCT: randomised controlled trial; RECOVERY: Randomised Evaluation of COVID-19 Therapy; REMAP-CAP: Randomised, Embedded, Multifactorial, Adaptive Platform trial for Community-Acquired Pneumonia; RKI: Robert Koch Institute (Germany); SAGE: Scientific Advisory Group for Emergencies (UK); SPOR: Strategy for Patient-Oriented Research (Canada); SUS: Unified Health System (Brazil); UBC: University of British Columbia; UK: United Kingdom; US: United States; WHO: World Health Organization.

Acknowledgements

We are most grateful to the reviewer for the various perceptive comments that have considerably improved the manuscript, and colleagues who most helpfully suggested further sources and ideas and also kindly commented on parts of the drafts, in particular: Sarah Thackway and Tari Turner from Australia; Mário Fabrício Fleury Rosa from Brazil; Martin Schechter, Victoria Schuckel, Cara Tannenbaum, and a CIHR staff member from Canada; Lisa Pfadenhauer and Brigitte Strahwald from Germany; Alexandra Boyle, Jessica Glen and Tania Pocock from New Zealand; Bill Maton-Howarth and Bryony Soper from the UK; Tracey Koehlmoos from the US. As always, responsibility for the final version remains with the authors.
